# 4-Hydroxypiperidines and Their Flexible 3-(Amino)propyloxy Analogues as Non-Imidazole Histamine H_3_ Receptor Antagonist: Further Structure–Activity Relationship Exploration and In Vitro and In Vivo Pharmacological Evaluation

**DOI:** 10.3390/ijms19041243

**Published:** 2018-04-19

**Authors:** Beata Olszewska, Anna Stasiak, Daniel McNaught Flores, Wiesława Agnieszka Fogel, Rob Leurs, Krzysztof Walczyński

**Affiliations:** 1 Department of Synthesis and Technology of Drugs, Medical University of Lodz, Muszyńskiego Street 1, 90-145 Łódź, Poland; 2 Department of Hormone Biochemistry, Medical University of Lodz, Żeligowskiego Street 7/9, 90-752 Łódź, Poland; anna.stasiak@umed.lodz.pl (A.S.); wieslawa.agnieszka.fogel@umed.lodz.pl (W.A.F.); 3 Amsterdam Institute of Molecules, Medicines & Systems, Division of Medicinal Chemistry, Vrije Universiteit Amsterdam, De Boelelaan 1108, 1081 HZ Amsterdam, The Netherlands; d.mcnaughtflores@vu.nl (D.M.F.); r.leurs@vu.nl (R.L.)

**Keywords:** histamine H_3_ receptor non-imidazole antagonists, *N*-methyl-5-{[1-(ω-substitutedalkyl)piperidin-4-yl]oxy}-*N*-propylpentan-1-amines, 5-{3-[ω-substitutedalkyl](methyl)aminopropoxy}-*N*-methyl-*N*-propylpentan-1-amines

## Abstract

Presynaptic histamine H_3_ receptors (H_3_R) act as auto- or heteroreceptors controlling, respectively, the release of histamine and of other neurotransmitters in the central nervous system (CNS). The extracellular levels of several neurotransmitters are enhanced by H_3_R antagonists, and there is a great interest for potent, brain-penetrating H_3_ receptor antagonists/inverse agonists to compensate for the neurotransmitter deficits present in various neurological disorders. We have shown that 1-[(benzylfuran-2-yl)methyl]piperidinyl-4-oxyl- and benzyl- derivatives of *N*-propylpentan-1-amines exhibit high in vitro potencies toward the guinea pig H_3_ receptor (jejunum), with pA_2_ = 8.47 and 7.79, respectively (the reference compound used was thioperamide with pA_2_ = 8.67). Furthermore, following the replacement of 4-hydroxypiperidine with a 3-(methylamino)propyloxy chain, the pA_2_ value for the first group decreased, whereas it increased for the second group. Here, we present data on the impact of elongating the aliphatic chain between the nitrogen of 4-hydroxypiperidine or 3-(methylamino)propan-1-ol and the lipophilic residue. Additionally, the most active compound in this series of non-imidazole H_3_ receptor antagonists/inverse agonists, i.e., **ADS-003**, was evaluated for its affinity to the recombinant rat and human histamine H_3_ receptors transiently expressed in HEK-293T cells. It was shown that **ADS-003**, given parenterally for 5 days, reduced the food intake of rats, as well as changed histamine and noradrenaline concentrations in the rats’ brain in a manner and degree similar to the reference H_3_ antagonist Ciproxifan.

## 1. Introduction

The histamine H_3_ receptors were first identified in 1983 in the rat brain by J.-Ch. Schwartz and coworkers [1]. The presence of H_3_ receptors was confirmed in the human brain a few years later [2]. The cDNA encoding the human H_3_ receptor was successfully cloned and functionally expressed by Lovenberg et al. [3]. Histamine H_3_ receptors predominantly have a presynaptic localization in histaminergic or other neurons and they modulate, through a negative feedback mechanism, the biosynthesis and release of histamine as autoreceptors [4] or the release of various other neurotransmitters, including norepinephrine [5], dopamine [6], serotonin [7], gamma-aminobutyric acid (GABA) [8], glutamate [9], and acetylcholine [10], acting as heteroreceptors. Consequently, it has been observed that administration of H_3_-antagonists to the CNS enhances neurotransmission and improves cognition and attention in relevant animal models of CNS diseases [11]. Therefore, H_3_-antagonists/inverse agonists have been proposed for the treatment of cognitive disorders, such as attention-deficit hyperactivity disorder (ADHD) [12] and Alzheimer’s disease [13] as well as of memory and learning deficits [10]. They may also be useful in epilepsy [14], schizophrenia [15], and obesity [16].

The discovery of thioperamide [17], the prototype of the H_3_ antagonist, led to the development of a wide range of imidazole-containing ligands [18]. Although many of them have found utility as pharmacological tools, the presence of an imidazole ring greatly limited brain penetration [19] and also introduced the potential for cytochrome P450 interactions [20]. For these reasons, efforts have been directed toward the design and synthesis of non-imidazole H_3_ antagonists with a good binding affinity, CNS penetration ability, and reduced/no potential for cytochrome P_450_ inhibition. A number of such antagonists with high selectivity and specificity have since been reported [21].

**Figure 1:**
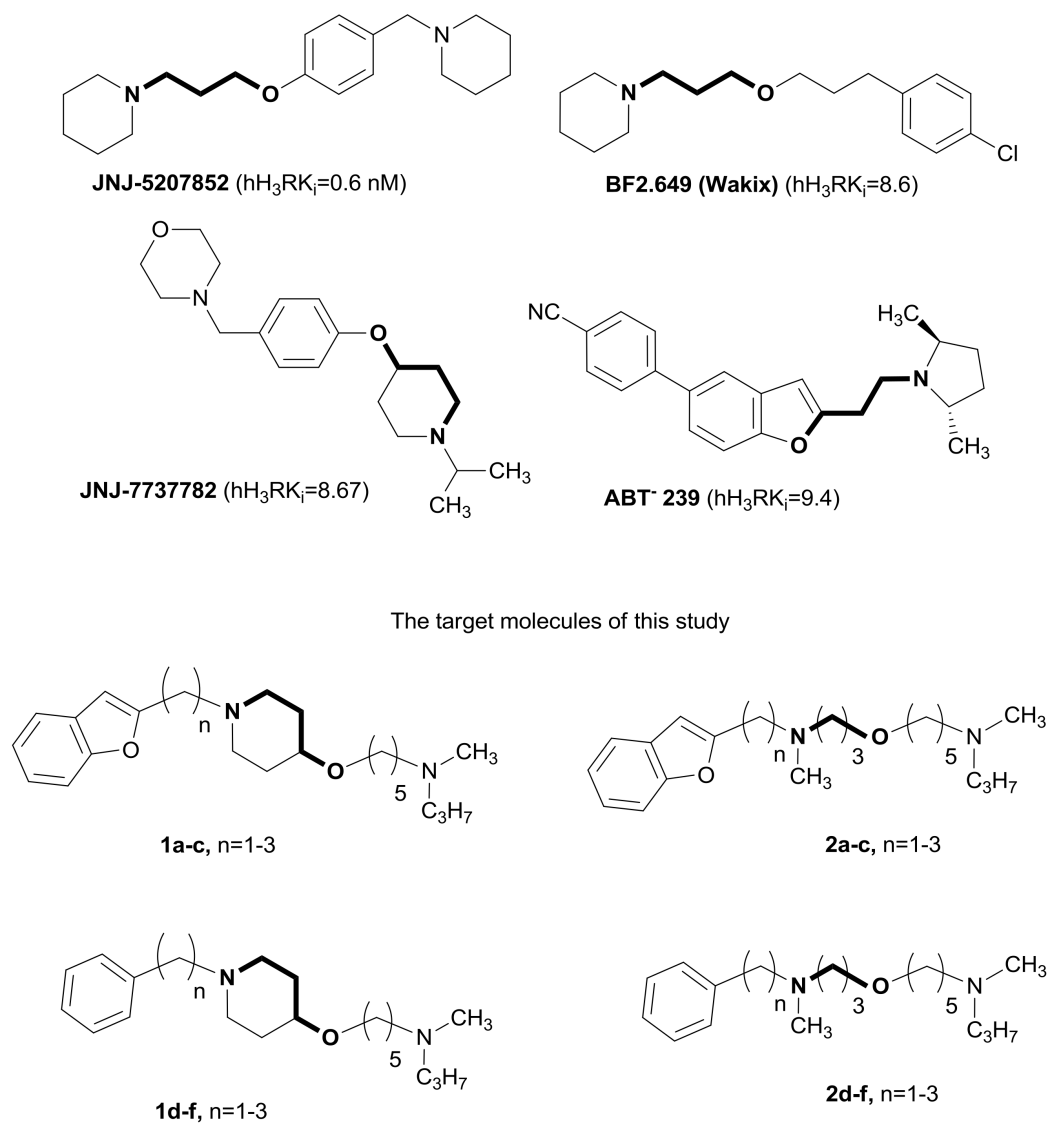
Representative non-imidazole H_3_-histamine receptor antagonists containing the characteristic 3-aminopropan-1-ol functionality, its rigid analogues, and the target molecules of this study.

The marine natural product aplysamine-1 patented by the Harbor Branch Oceanographic Institution as a weak histamine H_3_ receptor antagonist [22], possesses the characteristic 3-aminopropan-1-ol functionality in its structure [23]. This moiety has successfully been used by several laboratories for the development of non-imidazole histamine H_3_ receptor antagonists and resulted in a number of highly potent and selective compounds, for example, JNJ-5207852 [24] and Pitolisant BF2.649 (Wakix) [25], the potent and selective H_3_R antagonist, which was approved by the European Medicine Agency (EMA) in March 2016 for the treatment of the orphan disease narcolepsy with and without cataplexy (Figure 1). Later on, the successful replacement of the highly flexible 3-aminopropyloxy link with the 4-phenoxypiperidine moiety JNJ-7737782 [26] (Figure 1) or the partially rigid 2-aminoethylbenzofuran substructure ABT-239 [27] (Figure 1) was demonstrated.

**Scheme 1:**
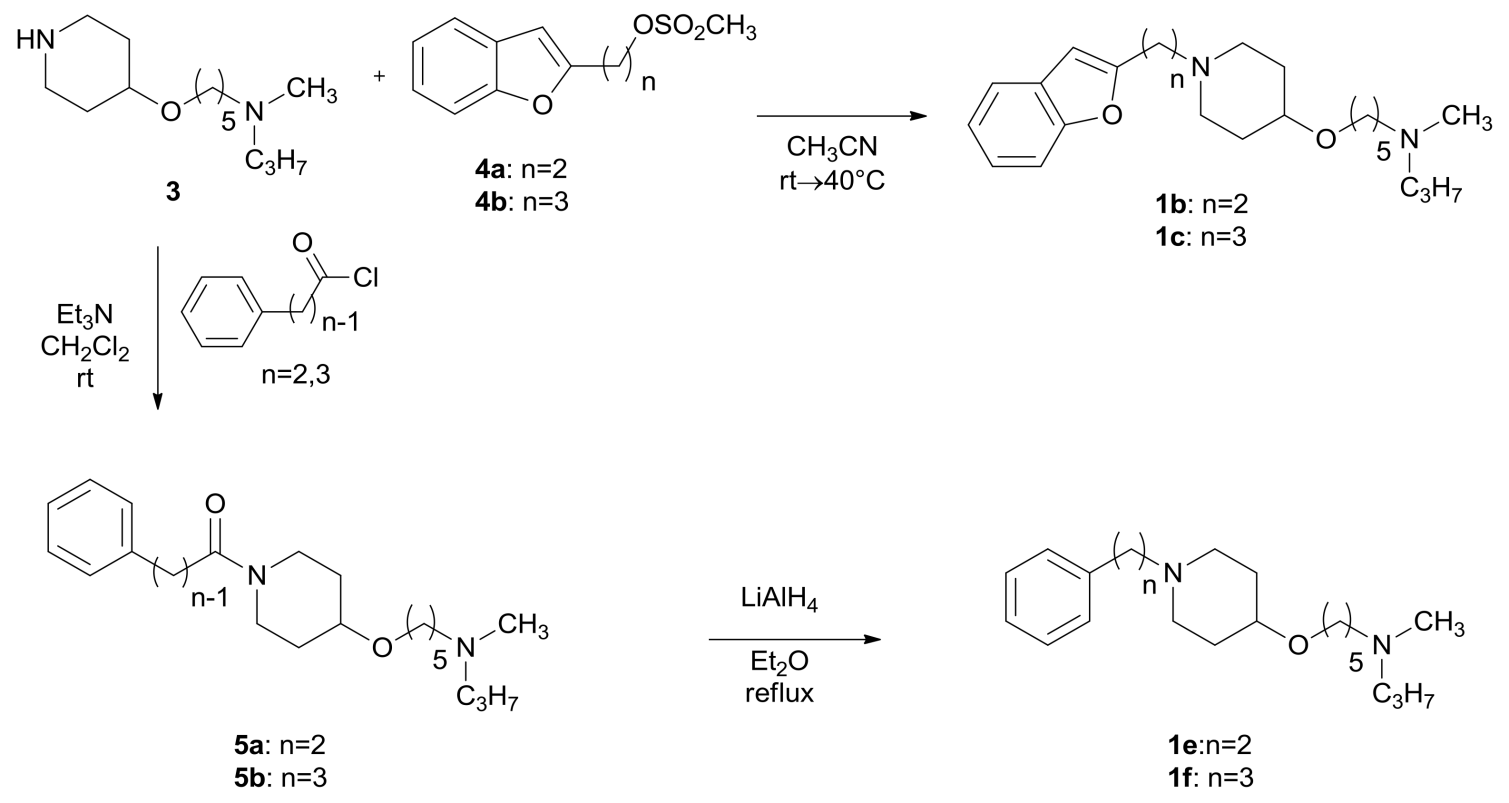
Synthetic routes to compounds **1b**,**c**,**e**,**f**.

**Scheme 2:**
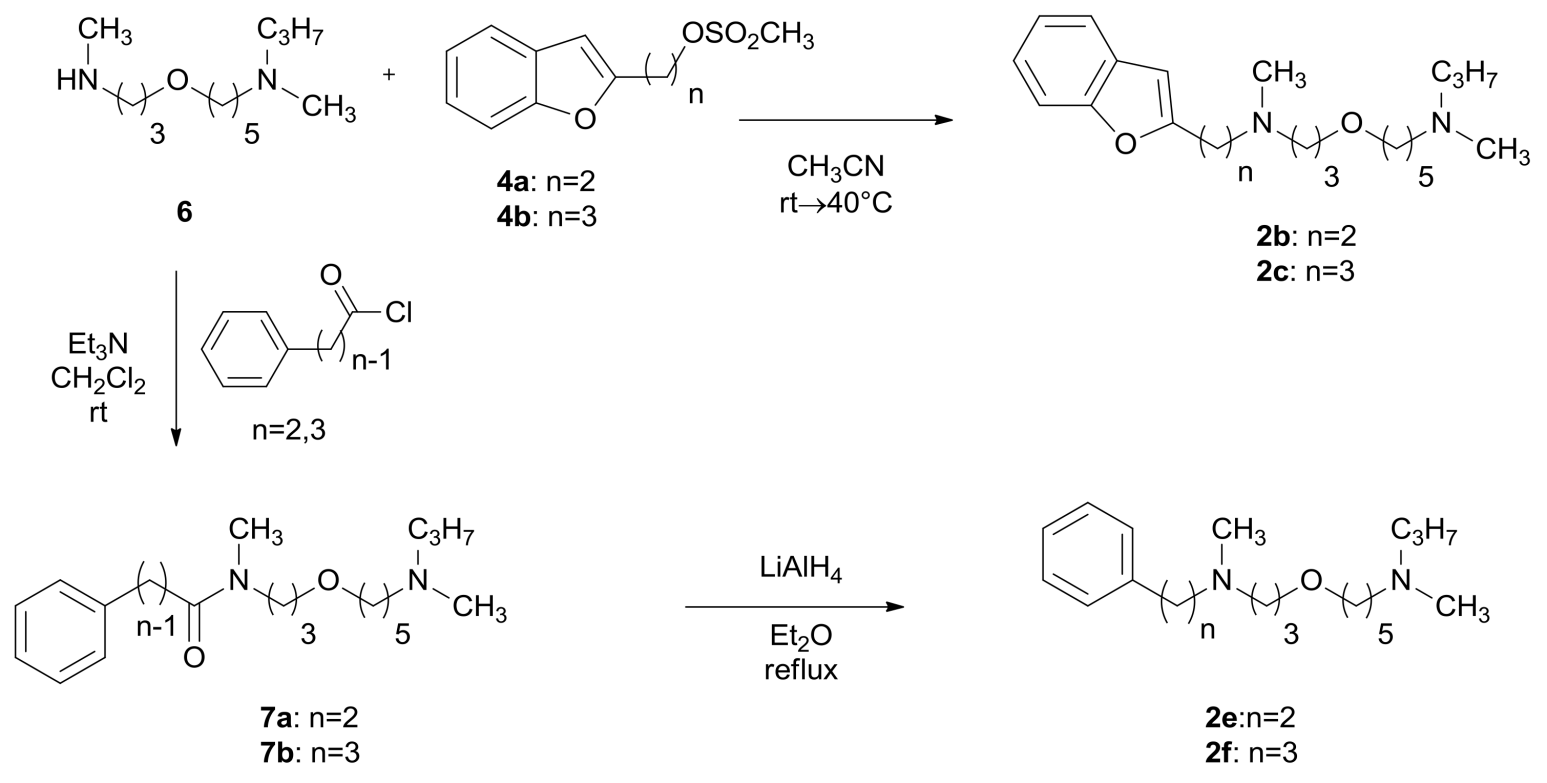
Synthetic routes to compounds **2b**,**c**,**e**,**f**.

Previously, our laboratory has described several non-imidazole piperazine- [28,29] and 4-hydroxypiperidine-based histamine H_3_ antagonists with a moderate to pronounced affinity for the receptor [30,31]. The structure–activity relationship (SAR) of 4-hydroxypiperidines series showed that the most potent compounds, under in vitro screening conditions, were the benzofuranylpiperidinyloxy 1a (**ADS-003**) (pA_2_ = 8.47; Figure 1) [31] (for reference, thioperamide pA_2_ = 8.67) and benzyl **1d** (pA_2_ = 7.79; Figure 1) derivatives [30]. None of the compounds showed any activity following binding to the histamine H_1_ receptor. Two analogues of the compounds **1a** and **1d**, i.e., **2a** and **2d** (Figure 1), in which the 4-hydroxypiperidine ring was replaced by a flexible 3-(methylamino)propyloxy chain, were synthesized and pharmacologically evaluated in vitro. In the case of derivatives carrying a (benzofuran-2-yl)methyl substituent (**1a**), a drastic reduction in potency was observed (**2a**, pA_2_ = 6.23; Figure 1) [31]. For compounds bearing a benzyl substituent, an inverse relationship was observed—5-{3-[benzyl(methyl)amino]propoxy}-*N*-methyl-*N*-propylpentane-1-amine **2d** (pA_2_ = 8.06; Scheme 2) expressed higher potency than its 4-hydroxypiperidine analogue **2a** (pA_2_ = 7.79; Scheme 1) [31].

In continuation of our search for new highly active and selective non-imidazole histamine H_3_ receptor antagonists, the first step of this study aimed to clarify the significant difference between the potencies of the 4-hydroxypiperidine derivatives and their 3-(methylamino)propyloxy analogues, bearing ω-(benzofuran-2-yl)alkyl and ω-phenylakyl moieties, respectively. We synthesized and in vitro pharmacologically [32] evaluated a series of derivatives in which the aliphatic chain between the nitrogen of the 4-hydroxypiperidine or the 3-(methylamino)propan-1-ol and the lipophilic residue was elongated by two to three methylene groups (compounds **1b**,**c**,**e**,**f**, and **2b**,**c**,**e**,**f**; Figure 1, respectively). This was to check whether the earlier observed tendency would still be kept, or if it was an exceptional case only for short methyl chain. We also wanted to study how the elongation of the alkyl chain would affect the potency for the H_3_ receptor. Additionally, the affinity of **ADS-003**—the most active compound that we have so far synthesized in this series—was estimated for the recombinant rH_3_R and hH_3_R (respectively), transiently expressed in HEK-293T cells. This derivative has also been proven to cross the blood–brain barrier; given parenterally for 5 days, **ADS-003** reduced the food intake by rats as well as changed the cerebral histamine and noradrenaline concentrations in these animals in a manner and degree similar to the reference H_3_ antagonist—Ciproxifan.

## 2. Results and Discussion

### 2.1. Chemistry

**Scheme 3:**
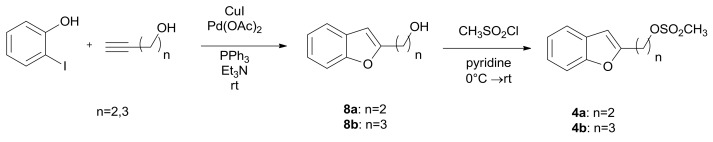
Synthesis of ω-(1-benzofuran-2-yl)alkyl methanesulfonates **4a**,**b**.

The general synthetic procedures used in this study are illustrated in Scheme 1 and 2. The key intermediates for all novel synthesized 4-hydroxypiperidines (**1b**,**c**,**e**,**f**; Scheme 1) and 3-(methylamino)propan-1-ols (**2b**,**c**,**e**,**f**; Scheme 2) were *N*-methyl-5-[(piperidin-4-yl)oxy]-*N*-propylpentan-1-amine (**3**) and *N*-methyl-5-[3-(methylamino)propoxy-*N*-propylpentane-1-amine (**6**), respectively. Both mentioned intermediates were prepared by hydrogenation of their corresponding benzyl derivatives with a catalytic amount of palladium in charcoal in ethanol [31].

The ω-(benzofuran-2-yl)alkyl derivatives of 4-hydoxypiperidine (**1b**,**c**; Scheme 1) and 3-(methyl)aminopropan-1-ol (**2b**,**c**; Scheme 2) were obtained from compounds **3** or **6** by alkylation with the corresponding ω-(benzofuran-2-yl)alkyl methanesulfonate in acetonitrile followed by purification by column chromatography.

The ω-phenylalkyl derivatives of 4-hydoxypiperidine (**1e**,**f**; Scheme 1) and 3-(methyl)aminopropan-1-ol (**2e**,**f**; Scheme 2) were obtained from compound **3** and **6** by a two-step synthesis: (1) acylation with the corresponding ω-phenylalkyl acid chloride in dry dichloromethane in the presence of triethylamine (TEA) to amides **5a**,**b** (Scheme 1) and **7a**,**b** (Scheme 2), (2) reduction of amides with LiAlH_4_ in dry ethyl ether to compounds **1e**,**f** (Scheme 1) and compounds **2e**,**f** (Scheme 2), respectively. Each step was followed by purification by column chromatography. 

The synthesis of the required intermediates **4a**,**b** (Scheme 3) was carried out by first treating the 2-iodophenol with the appropriate ω-alkyn-1-ol in the presence of palladium acetate, copper(I) iodide, and triphenylphosphine in dry triethylamine [33], followed by mesylation of the hydroxyl group by methanesulfonyl chloride in pyridine to yield the corresponding methanesulfonate with a 2 and 3 methylene linker. The detailed synthetic procedure and analytical data for compounds **8a**,**b** and **4a**,**b** are shown in Supplementary Material A (Section 1).

### 2.2. Pharmacology

#### 2.2.1. In Vitro Pharmacological Studies

##### H_3_ Antagonistic Activity for Compounds **1a**–**f**, and **2a**–**f**

All newly synthesized compounds were converted into their dihydrogenoxolates salts and in vitro evaluated as H_3_ receptor antagonists against H_3_ agonist-induced inhibition of the electrically evoked contraction of the guinea pig jejunum [32].

The potencies of compounds **1b**,**c**, **1e**,**f**, **2b**,**c**, and **2e**,**f**, are reported in Table 1, as well as the previously described data for compounds **1a**, [31] **1d [32]**, **2a [32]**, and **2d [32]**. Derivatives **1b**,**c**, **1e**,**f**, **2b**,**c**, and **2e**,**f**, showed moderate to weak antagonist activity at H_3_-receptor.

With the aim of clarifying the significant difference between the change in the potency of derivatives **1a**/**2a** (pA_2_ = 8.27/6.23) carrying a 2-methylbenzofuranyl substituent versus their benzyl-analogues **1d**/**2d** (pA_2_ = 7.79/8.06), we compared the homologues pairs of compounds **1b**/**2b** versus **1e**/**2e**, and **1c**/**2c** versus **1f**/**2f** (Table 1). It was found that **1b** (pA_2_ = 7.26) had a higher activity than its 3-(methylamino)propyloxy analogue **2b** (pA_2_ = 6.32), as also observed for derivatives **1a**/**2a**. In contrast, in the homologues pair **1e**/**2e**, the 3-(methylamine)propan-1-ol derivative **2e** (pA_2_ = 6.72) with the phenylethyl moiety showed antagonistic activity at the same level as its 4-hydroxy piperidine analogue **1e** (pA_2_ = 6.67). In contrast to the above results, further elongation of the alkyl chain to three methylene groups led to a decrease of potency for both pairs **1c**/**2c** versus **1f**/**2f.** Derivative **1c** (pA_2_ = 7.11), again, showed a higher potency than its 3-(methylamino)propyloxy analogue **2c** (pA_2_ = 6.37), but the phenylpropyl derivative of 4-hydroxypiperidine **1f** (pA_2_ = 7.51) had a higher potency than its analogue **2f** (pA_2_ = 6.79), which is in opposition to the previously obtained results for derivatives **1d**/**2d.**

The differences were observed within the **1a**–**c** and **1d**–**f** series. While the previously reported 4-hydroxypiperidine derivative bearing a 2-benzofuranylmethyl moiety **1a** showed a high potency, the newly synthesized compounds **1b**,**c**, where the aliphatic chain between the piperidine nitrogen and the benzofuranyl residue is elongated from 2 to 3 methylene groups, resulted in a decrease of potency (pA_2_ = 7.26 and 7.11, respectively). A similar effect was observed when the 2-benzofuranylmethyl substituent was replaced by a ω-phenylakyl one (compounds **1d**–**f**). In this series, derivative **1d** (pA_2_ = 7.79) showed a high potency, but an increase in the alkyl chain length to 2 methylene groups resulted in a decrease of antagonist activity for compound **1e** (pA_2_ = 6.67), and the activity increased again on further lengthening to 3 methylene groups (**1f**; pA_2_ = 7.51). 

In the series of derivatives **2a**–**c**, bearing a ω-(benzofuran-2-yl)alkyl substituent—analogues of compounds **1a**–**c**—only a weak activity (pA_2_ ranging from 6.23 to 6.37), independent of the alkyl chain length, was observed. Elongation of the alkyl chain from 2 to 3 methylene groups in the analogue series of compounds **1e**,**f** i.e., **2e**,**f** resulted in a drastic reduction of potency (pA_2_ = 6.72 and 6.79, respectively) in comparison to the parent compound **2d** (pA_2_ = 8.06).

Representative graphs of the antagonism by **ADS-003** (**1a**) and thioperamide of the inhibitory effect of *R*-(−)-α-methylhistamine (R-α-MH) on the electrically induced contraction of guinea pig ileum strips are shown in Supplementary Material B (Section 2.2.1; Figure S2).

##### H_1_ Antagonistic Activity for Compounds **1b** and **1f**

The compounds **1b** and **1f**, possessing the highest potency for the H_3_ receptors, were also tested for H_1_ antagonistic effects in vitro, using standard methods [34]. Derivatives **1b** and **1f** did not show any antagonistic activity for the H_1_-receptor (pA_2_ < 4; for pyrilamine pA_2_ = 9.37).

#### 2.2.2. Histamine H_3_ Receptor Affinity

The affinity, based on the SARs obtained for both series (compounds: **1a**–**f** and **2a**–**f**), of the most active compound **1a** (**ADS-003**) was evaluated by measuring the displacement curve of [^3^H]-*N*^α^-methylhistamine from the rat (rH_3_R) and human histamine H_3_ receptor (hH_3_R) in HEK-293Tcell membranes, as described by Bonger [35].

##### Saturation of Rat and Human H_3_ Receptors

The saturation of rat and human H_3_ receptors were carried out as described previously [36]. 

A representative graph of saturation of rat and human H_3_R can be found in Supplementary Material B (Section 2.2.2; Figure S3).

The analysis of the [^3^H]-*N*^α^-MH saturation binding yielded, at rH_3_R, a *K*_D_ value of 2.72 ± 0.34 nM and a *B*_max_ value of 2715 ± 445 fmol/mg protein and, at hH_3_R, a *K*_D_ value of 0.9 ± 0.08 nM and a *B*_max_ value of 632 ± 52 fmol/mg protein.

##### Competition Binding of H_3_ Receptor Ligands

The affinity of **ADS-003** (**1a**), histamine, and thioperamide was determined—the reference compounds were evaluated by measuring the displacement curves of [^3^H]-*N*^α^-methylhistamine binding to rat and human histamine H_3_ receptor expressed in HEK-293T membranes. Derivative **ADS-003** (**1a**) possessed a high nanomolar affinity for the rat H_3_R, with a pK_i_ value of 7.9 ± 0.1, similar to thioperamide (pK_i_ value 7.9 ± 0.1) and slightly higher than histamine (pK_i_ = 7.3 ± 0.1). A significantly lower affinity was observed for **ADS-003** (**1a**) for the human H_3_R, with pK_i_ 6.6 ± 0.1, in comparison with the pK_i_ of thioperamide (7.2 ± 0.1) and the pK_i_ of histamine (7.7 ± 0.1). Representative graphs of competition binding of H_3_R ligands to rat and human H_3_ receptor are shown in Supplementary Material B (Section 2.2.2; Figures S4 and S5, respectively). 

#### 2.2.3. Verification of In Vivo Activity for Compound **ADS-003** (**1a**)

Finally, compound **ADS-003** (**1a**) was subjected to an in vivo evaluation of its impact on brain neurotransmitter systems. This assessment concerned:

- The effects of the compound on the feeding behavior of rats after its repeated peripheral administration. Given that the compound enters the CNS and blocks the H_3_R, it should release histamine. Histamine, in turn, acting via H_1_R, would induce loss of appetite, i.e., the food intake of rats would decrease,
- The influence on the cerebral amine neurotransmitter concentrations, as well as the activity of the monoamine oxidases A and B and histamine *N*-methyltransferase. The latter was accomplished by postmortem analyses of the brain tissues of the treated rats.

**Figure 2:**
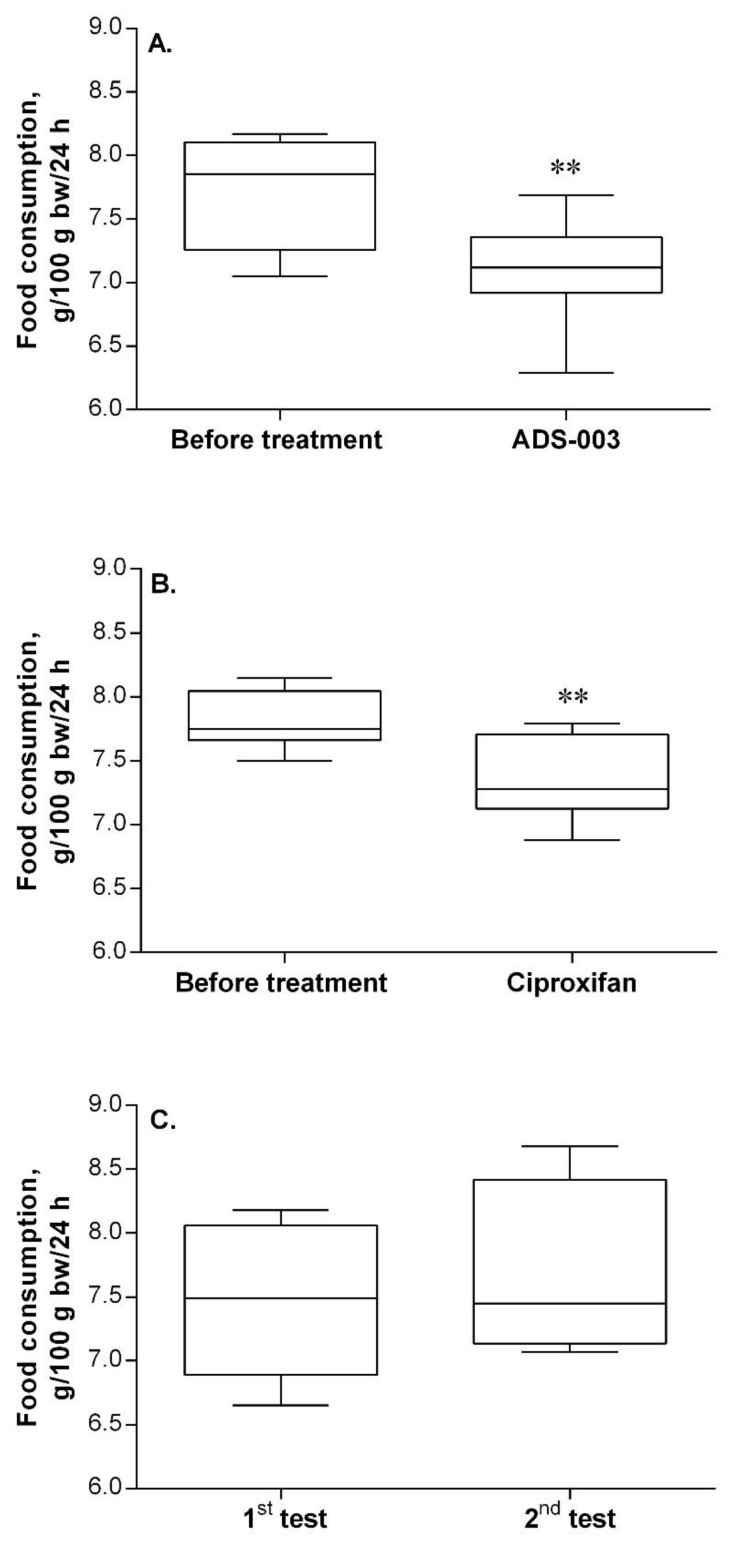
The effect of **ADS-003** (s.c. 3 mg/kg/daily for 5 days), the newly synthesized histamine H_3_ receptor antagonist (**A**), and of the reference compound Ciproxifan (s.c. 3 mg/kg/daily for 5 days; **B**) on food intake. Consumption by untreated rats (**C**). The median (the line in the middle of the box) and the range of values (whiskers) are given for eight rats. Paired *t*-test: ** *p* < 0.01 versus before treatment.

To evaluate the central effects of peripheral administration of **ADS-003** (**1a**) to rats, the feeding behavior of rats was monitored after drug administration. The neuronal histaminergic system is known to be one of the regulatory systems in food intake. Studies in various animal models have convincingly shown that histamine H_1_R and H_3_R receptors play an important role in this respect. Activation of both histamine receptors is a critical part of the diurnal rhythm of the food consumption regulatory mechanism, as well as in energy intake and expenditure [37,38,39,40,41]. In a comprehensive study done in rats, it was demonstrated that centrally infused histamine or H_1_ receptor agonists invariably decreased food intake, as did the strategies leading either to an enhanced release of hypothalamic histamine by the blocking the H_3_ receptor or to an increase of available histamine by inhibiting its degradation. The opposite, i.e., hyperphagia, was seen for H_1_ antagonists [38]. Compatible with the earlier reported experimental data, it was assumed that, if an H_3_ receptor antagonist subcutaneously injected into rats crossed the blood–brain barrier (BBB), it would affect the animal food consumption [37]. As presented in Figure 2, the treatment of rats with either of the two H_3_R antagonists **ADS-003** (3 mg/kg s.c.) and **Ciproxifan** (3 mg/kg s.c.) over a period of 5 days evoked a statistically significant reduction in the amount of consumed food by rats compared to the pre-treatment period. 

In in vivo studies, **Ciproxifan** was used as a reference instead of thioperamide, because the latter demonstrated lower bioavailability due to restricted brain penetration [42].

There was no difference in the efficacy of **ADS-003** (**1a**) and **Ciproxifan** (the reference compound). These results suggest that **ADS-003** crosses the BBB, and its potency at H_3_R is similar to that of **Ciproxifan**. 

##### Post-Mortem Biochemical Analysis of the Brain Tissues of **ADS-003** (**1a**)-Treated Rats

**Figure 3:**
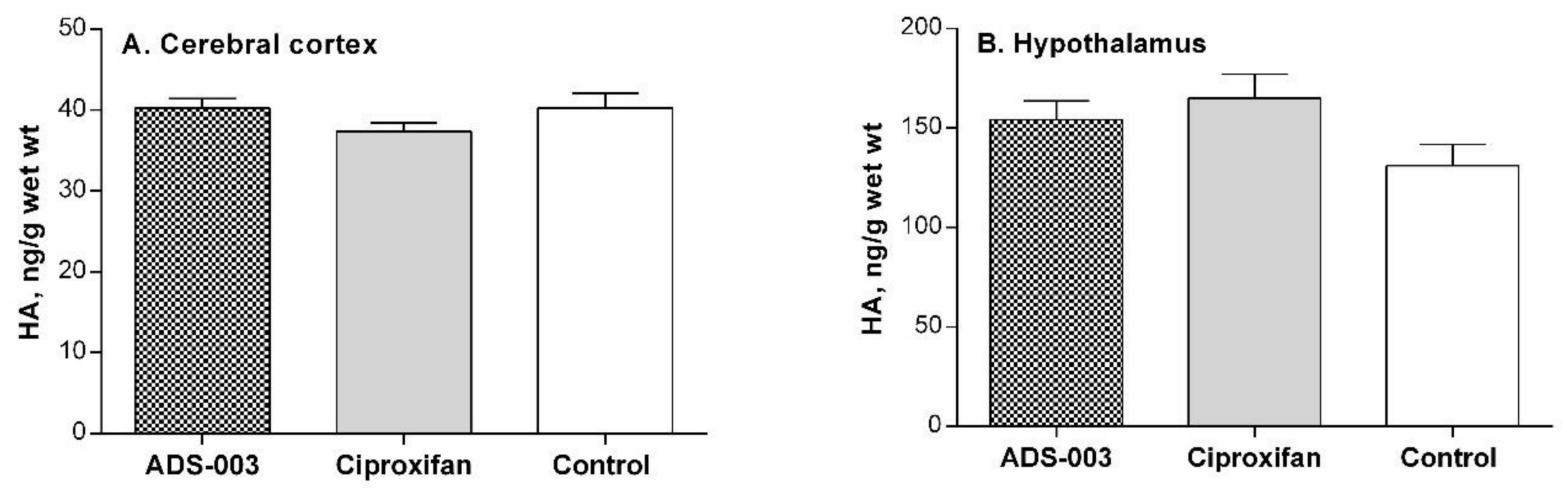
Cerebral histamine concentration in rats subchronically treated with Ciproxifan and the newly synthesized **ADS-003** histamine H_3_ receptor antagonist. The median (the line in the middle of the box) and the range of values (whiskers) are given for eight rats. Paired *t*-test: *p* < 0.05 versus “before treatment” for eight rats. Paired *t*-test, *p* < 0.05, *p* < 0.01 versus “before treatment”.

Postmortem biochemical analysis of the brain tissues of **ADS-003**-treated rats quantified the brain concentration of histamine, serotonin, dopamine, noradrenaline, and the activities of monoamine oxidase (MAO)-A, MAO-B, and HNMT. As shown in Figure 3, the histamine concentration in the hypothalamus, where histaminergic cell bodies are located, showed a tendency to increase—which could be explained by the stimulation of the amine synthesis following its release by H_3_R blockade with **1a** (**ADS-003**) or **Ciproxifan** to replenish vesicular stores. Yet, one-way ANOVA and Tukey’s multiple comparisons test showed no statistically significant differences. Similarly, no changes were found in the histamine levels in the cerebral cortex of the treated rats (Figure 3).

**Figure 4:**
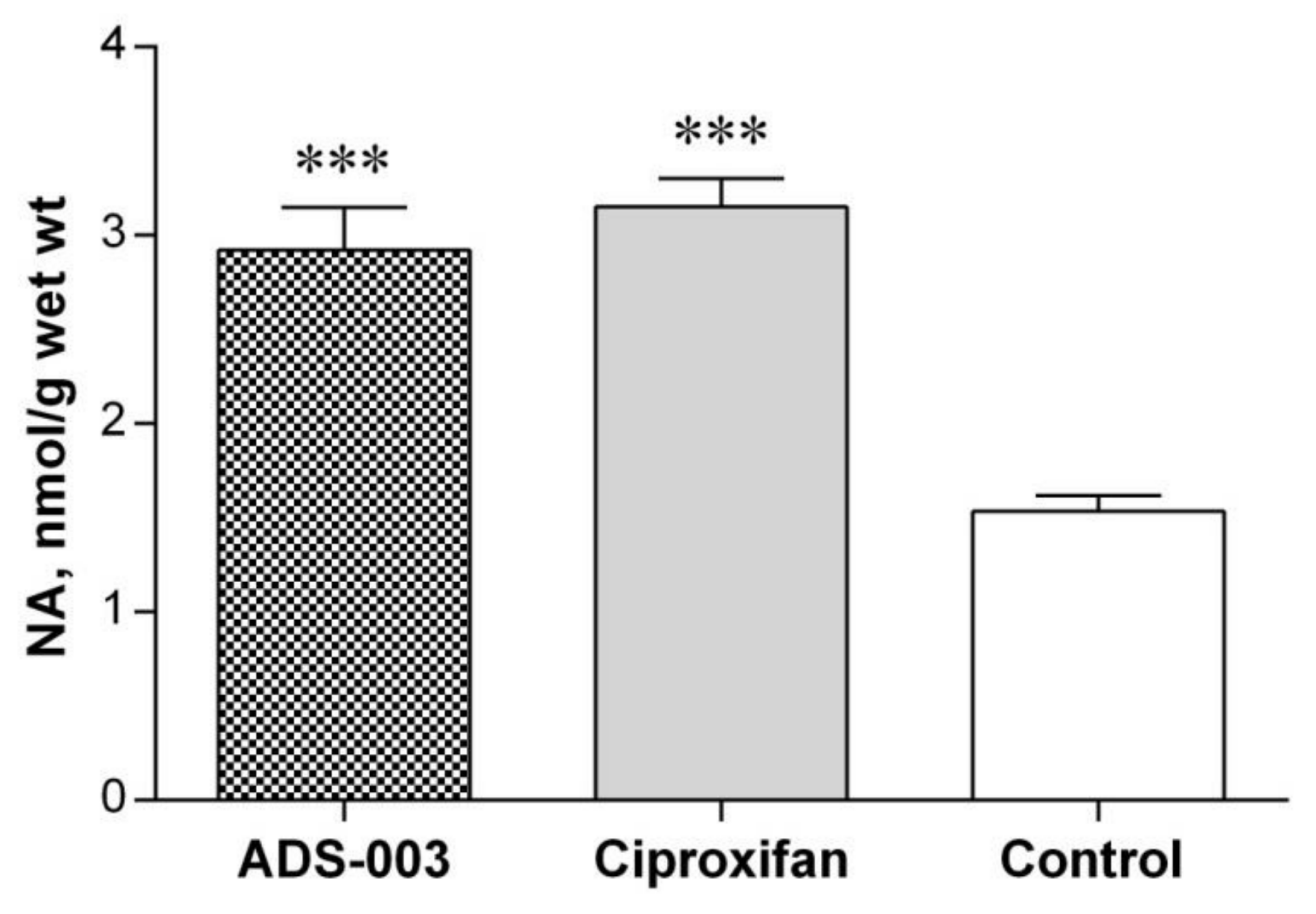
The concentration of noradrenaline (NA) in the cerebral cortex of rats subchronically treated with the newly synthesized **ADS-003** histamine H_3_ receptor antagonist or with Ciproxifan. The values are means ± SEM for four–nine rats. One-way ANOVA and Tukey’s multiple: *** *p* < 0.05, three symbols: *p* < 0.001.

On the other hand, both H_3_R antagonists caused a significant increase in noradrenaline levels in the cerebral cortex (Figure 4). 

There were no changes in serotonin and dopamine concentration.

The increase in tissue NA is compatible with previous data reporting an inhibitory control exerted by H_3_ histamine receptors on NA neuronal function in the cortex [43,44]. The fact that both histamine H_3_ receptor antagonists, Ciproxifan and **ADS-003**, enhanced the tissue levels of NA in a similar manner strengthens this idea.

Using sensitive isotopic assays, neither changes in monoamine oxidase A and B nor in histamine *N*-methyltransferase in the brain tissues of rats were observed (Table 2).

## 3. Materials and Methods

### 3.1. Chemistry

#### 3.1.1. General Methods

All melting points (mp) were measured in open capillaries in an electrothermal apparatus and are uncorrected. The ^1^H NMR spectra were recorded in CDCl_3_ as a solvent in a 600 MHz spectrometer, a Bruker Avance III spectrometer at ambient temperature. The chemical shifts are reported in ppm on scale downfield from tetramethylsilane (TMS ) as internal standard, and the signal patterns are indicated as follows: s = singlet, d = doublet, t = triplet, m = multiplet, br = broad; * exchangeable by D_2_O; a number of protons, and J approximate coupling constant in Hertz. The ^13^C NMR spectra were recorded in a 600 MHz spectrometer, a Bruker Avance III (150 MHz). Elemental analyses (C, H, N) for all compounds were measured in Perkin Elmer Series II CHNS/O Analyzer 2400 and agreed with the theoretical values within ±0.4%. TLC data were obtained with Merck silica gel 60F_254_ aluminum sheets. For flash column chromatography using silica gel, 60 Å, 50 μm (J. T. Baker B. V.), the same solvent system as for TLC, was used. All obtained final free bases were treated with methanolic oxalic acid, and the dihydrogenoxolates were precipitated with dry diethyl ether and crystallized twice from ethanol. All dihydrogenoxolates were obtained as white crystalline solids. 

##### Chemicals

The reagents 2-iodophenol, palladium acetate, copper(I) iodide, triphenylphosphine, 3-butyn-1-ol, 4-pentyn-1-ol, methanesulfonyl chloride, lithium aluminum hydride, and all solvents were purchased from Sigma-Aldrich (Saint Louis, MO, USA) and Alfa Aesar (Haverhill, MA, USA) and were used without any purification.

#### 3.1.2. General Procedure for the Preparation of Compounds **1b**,**c** and **2b**,**c**

To a solution of the corresponding amines **3** or **6** (1.26 mmol) in acetonitrile (5 mL), the appropriate methanesulfonate **4a** or **4b** (1.05 mmol) was added. The reaction mixture was stirred at 40 °C for 24 h. After completion of the reaction, water was added. The mixture was extracted with dichloromethane (3 × 20 mL), and the organic layer was dried over MgSO_4_ and filtered. The solvent was evaporated, and the residue was purified by silica gel-flash column chromatography to yield a sticky oil.

*5-{{1-[2-(1-Benzofuran-2-yl)ethyl]piperidin-4-yl}oxy}-N-methyl-N-propylpentan-1-amine* (**1b**): (119 mg, 29.0%): R_f_ = 0.49 (CH_2_Cl_2_/MeOH/NH_3(aq)_ 8:1:1%); ^1^H NMR (600 MHz, CDCl_3_): δ = 0.90 (t, *J* = 7.4 Hz, 3H, NCH_2_CH_2_CH_3_), 1.34–1.38 (m, 2H, H-3), 1.49–1.65 (m, 10H, H-2, H-4, NCH_2_CH_2_CH_3_, 2xCH_2_^pip^), 1.87–1.92 (m, 2H, CH_2_^pip^), 2.28 (s, 3H, CH_3_), 2.36 (t, *J* = 7.8 Hz, 2H, H-5), 2.41 (t, *J* = 7.7 Hz, 2H, NCH_2_CH_2_CH_3_), 2.76 (t, *J* = 7.4 Hz, 2H, C_8_H_5_OCH_2_CH_2_N), 2.81–2.83 (m, 2H, CH_2_^pip^), 2.97 (t, *J* = 7.4 Hz, 2H, C_8_H_5_OCH_2_CH_2_N), 3.27–3.31 (m, 1H, CH^pip^), 3.42 (t, *J* = 6.5 Hz, 2H, H-1), 6.42 (s, 1H, CH^furan^), 7.15–7.21 (m, 2H, C_6_H_4_), 7.39 (d, *J* = 7.6 Hz, 1H, C_6_H_4_), 7.46 ppm (d, *J* = 7.2 Hz, 1H, C_6_H_4_); ^13^C NMR (150 MHz, CDCl_3_): δ = 12.04 (NCH_2_CH_2_CH_3_), 20.08 (NCH_2_CH_2_CH_3_), 24.41 (C-3), 26.73 (C_8_H_5_OCH_2_CH_2_N), 29.90 (CH_2_^pip^), 30.19 (C-4), 31.08 (C-2), 42.05 (CH_3_), 51.28 (CH_2_^pip^), 56.59 (C_8_H_5_OCH_2_CH_2_N), 57.59 (C-5), 59.61 (NCH_2_CH_2_CH_3_), 67.93 (C-1), 75.06 (CH^pip^), 102.66 (C^furan^), 110.94, 120.47, 122.66, 123.43,129.12, 154.85 (C_6_H_4_), 157.83 ppm (C^furan^).

Anal. calcd for dihydrogenoxolate (C_24_H_38_N_2_O_2_ 2C_2_H_2_O_4_)_:_ C 59.35, H 7.47, N 4.94; found: C 59.08, H 7.83, N 5.07; mp_dihydrogenoxolate_ = 133.2–134.9 °C.

*5-{{1-[3-(1-Benzofuran-2-yl)propyl]piperidin-4-yl}oxy}-N-methyl-N-propylpentan-1-amine* (**1c**): (226 mg, 54.0%): R_f_ = 0.38 (CH_2_Cl_2_/MeOH/NH_3(aq)_ 8:1:1%); ^1^H NMR (600 MHz, CDCl_3_): δ = 0.88 (t, *J* = 7.4 Hz, 3H, NCH_2_CH_2_CH_3_), 1.31–1.36 (m, 2H, H-3), 1.45–1.51 (m, 4H, NCH_2_CH_2_CH_3_, CH_2_^pip^), 1.55–1.62 (m, 6H, H-2, H-4, CH_2_^pip^), 1.87–1.89 (m, 2H, CH_2_^pip^), 1.91–1.94 (m, 2H, C_8_H_5_OCH_2_CH_2_CH_2_N), 2.20 (s, 3H, CH_3_), 2.28 (t, *J* = 7.7 Hz, 2H, H-5), 2.32 (t, *J* = 7.8 Hz, 2H, NCH_2_CH_2_CH_3_), 2.39 (t, *J* = 7.6 Hz, 2H, C_8_H_5_OCH_2_CH_2_CH_2_N), 2.74–2.80 (m, 4H, CH_2_^pip^, C_8_H_5_OCH_2_CH_2_CH_2_N) 3.24–3.27 (m, 1H, CH^pip^), 3.42 (t, *J* = 6.6 Hz, 2H, H-1), 6.37 (s, 1H, CH^furan^), 7.14–7.20 (m, 2H, C_6_H_4_), 7.38 (d, *J* = 7.8 Hz, 1H, C_6_H_4_), 7.46 ppm (d, *J* = 7.2 Hz, 1H, C_6_H_4_); ^13^C NMR (150 MHz, CDCl_3_): δ = 12.15 (NCH_2_CH_2_CH_3_), 20.60 (NCH_2_CH_2_CH_3_), 24.46 (C_8_H_5_OCH_2_CH_2_CH_2_N), 25.49 (C-3), 26.64 (C_8_H_5_OCH_2_CH_2_CH_2_N), 27.32 (C-4), 30.29 (CH_2_^pip^), 31.65 (C-2), 42.49 (CH_3_), 51.56 (CH_2_^pip^), 57.95 (C_8_H_5_OCH_2_CH_2_CH_2_N and C-5), 60.06 (NCH_2_CH_2_CH_3_), 67.98 (C-1), 74.06 (CH^pip^), 102.66 (C^furan^), 110.94, 120.47, 122.66, 123.43, 129.12, 154.85 (C_6_H_4_), 157.83 ppm (C^furan^).

Anal. calcd for dihydrogenoxolate (C_25_H_40_N_2_O_2_ 2C_2_H_2_O_4_ 0.5 H_2_O): C 59.07, H 7.69, N 4.75; found: C 59.05, H 7.75, N 4.80; mp_dihydrogenoxolate_ = 157–159 °C.

*5-{3-{[2-(Benzofuran-2-yl)ethyl](methyl)amino}propoxy}-N-methyl-N-propylpentan-1-amine* (**2b**): (109 mg, 28.0%): R_f_ = 0.51 (CH_2_Cl_2_/MeOH/NH_3(aq)_ 8:1:1%);^1^H NMR (600 MHz, CDCl_3_): δ = 0.88 (t, *J* = 7.4 Hz, 3H, NCH_2_CH_2_CH_3_), 1.31–1.36 (m, 2H, H-6), 1.43–1.50 (m, 4H, H-7, NCH_2_CH_2_CH_3_), 1.54–1.59 (m, 2H, H-5), 1.72–1.77 (m, 2H, H-2), 2.19 (s, 3H, CH_3_), 2.27 (t, *J* = 7.6 Hz, 2H, NCH_2_CH_2_CH_3_), 2.29–2.32 (m, 5H, H-8, CH_3_), 2.49 (t, *J* = 7.2 Hz, 2H, H-1), 2.76–2.79 (m, 2H, C_8_H_5_OCH_2_CH_2_N), 2.91–2.95 (m, 2H, C_8_H_5_OCH_2_CH_2_N), 3.36 (t, *J* = 6.7 Hz, 2H, H-4), 3.42 (t, *J* = 6.5 Hz, 2H, H-3), 6.41 (s, 1H, CH^furan^), 7.13–7.20 (m, 2H, C_6_H_4_), 7.38–7.40 (m, 1H, C_6_H_4_), 7.45–7.47 ppm (m, 1H, C_6_H_4_); ^13^C NMR (150 MHz, CDCl_3_): δ = 12.15 (NCH_2_CH_2_CH_3_), 20.65 (C-7), 24.44 (C-6), 26.92 (NCH_2_CH_2_CH_3_), 27.37 (C-2), 27.92 (C_8_H_5_OCH_2_CH_2_N), 29.94 (C-5), 42.33, 42.49 (2× CH3), 54.58 (C-1), 55.83 (C_8_H_5_OCH_2_CH_2_N), 58.00 (NCH_2_CH_2_CH_3_), 60.10 (C-8), 69.23 (C-4), 71.13 (C-3), 102.64 (C^furan^), 110.91, 120.43, 122.62, 123.36, 129.17, 154.87 (C_6_H_4_), 158.00 ppm (C^furan^).

Anal. calcd for dihydrogenoxolate (C_23_H_38_N_2_O_2_ 2C_2_H_2_O_4_·0.5 H_2_O): C 57.54, H 7.69, N 4.97; found: 5 C 7.37, 7.95 C H, N 5.02; mp_dihydrogenoxolate_= 144.5–145.7 °C.

*5-{3-{[3-(Benzofuran-2-yl)propyl](methyl)amino}propoxy}-N-methyl-N-propylpentan-1-amine* (**2c**): (134 mg, 34.0%): R_f_ = 0.45; (CH_2_Cl_2_/MeOH/NH_3(aq)_ 8:1:1%); ^1^H NMR (600 MHz, CDCl_3_): δ = 0.88 (t, *J* = 7.4 Hz, 3H, NCH_2_CH_2_CH_3_), 1.30–1.35 (m, 2H, H-6), 1.44–1.50 (m, 4H, H-7, NCH_2_CH_2_CH_3_), 1.55–1.59 (m, 2H, H-5), 1.71–1.76 (m, 2H, H-2), 1.88–1.93 (m, 2H, C_8_H_5_OCH_2_CH_2_CH_2_N), 2.19 (s, 3H, CH_3_), 2.23 (s, 3H, CH_3_), 2.28 (t, *J* = 7.6 Hz, 2H, NCH_2_CH_2_CH_3_), 2.31 (t, *J* = 7.6 Hz, 2H, H-8), 2.41–2.43 (m, 4H, H-3, C_8_H_5_OCH_2_CH_2_CH_2_N), 2.78 (t, *J* = 7.4 Hz, 2H, C_8_H_5_OCH_2_CH_2_CH_2_N), 3.38 (t, *J* = 6.7 Hz, 2H, H-4), 3.41–3.44 (m, 2H, H-1), 6.37 (s, 1H, CH^furan^), 7.14–7.20 (m, 2H, C_6_H_4_), 7.39 (d, *J* = 8.1 Hz, 1H, C_6_H_4_), 7.46 ppm (d, *J* = 8.1 Hz, 1H, C_6_H_4_); ^13^C NMR (150 MHz, CDCl_3_): δ = 12.09 (C-11), 20.39 (C-7), 24.39 (C-6), 25.67 (C-10), 26.50 (C-2), 27.83 (C_8_H_5_OCH_2_CH_2_CH_2_N), 29.90 (C_8_H_5_OCH_2_CH_2_CH_2_N and C-5), 42.30, 42.40 (2× CH3), 54.80 (C_8_H_5_OCH_2_CH_2_CH_2_N), 57.19 (C-1), 57.83 (C-9), 59.89 (C-8), 69.33 (C-3), 71.08 (C-4), 102.16 (C^furan^), 110.92, 120.39, 112.60, 123.30, 129.20, 154.89 (C_6_H_4_), 159.51 ppm (C^furan^).

Anal. calcd for dihydrogenoxolate (C_24_H_40_N_2_O_2_·2C_2_H_2_O_4_ 0.5 H_2_O): C 58.22, H 7.85, N 4.85; found: C 58.61, H 8.30, N 4.97; mp_dihydrogenoxolate_ = 106.0–108.0 °C.

#### 3.1.3. General Procedure for the Preparation of Compounds **5a**,**b** and **7a**,**b**

The corresponding amines **3** or **6** (2.6 mmol) and Et_3_N (3.12 mmol) were dissolved in dichloromethane and cooled to 0°C. After flushing with argon, 2-phenylacetyl chloride (3.12 mmol) or 3-phenylpropanoyl chloride (3.12 mmol) was slowly added to the mixture, and the reaction was stirred at room temperature for 12 h. After completion of the reaction, an aqueous solution of K_2_CO_3_ was added. The reaction mixture was extracted with dichloromethane (3 × 20 mL), and the organic layer was dried over MgSO_4_ and filtered. The solvent was evaporated, and the residue was purified by silica gel-flash column chromatography to yield a sticky oil.

*1-{4-{{5-[Methyl(propyl)amino]pentyl}oxy}piperidin-1-yl}-2-phenylethanone* (**5a**): (818 mg, 87.0%): R_f_ = 0.42 (CH_2_Cl_2_/MeOH/NH_3(aq)_ 8:1:1%); ^1^H NMR (600 MHz, CDCl_3_): δ = 0.89 (t, *J* = 7.3 Hz, 2H, NCH_2_CH_2_CH_3_), 1.30–1.35 (m, 3H, H-3, CH^pip^), 1.46–1.49 (m, 4H, NCH_2_CH_2_CH_3_, H-4), 1.53–1.57 (m, 4H, H-2, CH_2_^pip^), 1.74–1.80 (m, 1H, CH^pip^), 2.21 (s, 3H, CH_3_), 2.28 (t, *J* = 7.6 Hz, 2H, H-5), 2.32 (t, *J* = 7.6 Hz, 2H, NCH_2_CH_2_CH_3_), 3.17–3.21 (m, 1H, CH^pip^), 3.31–3.43 (m, 4H, H-1, CH^pip^, CH^pip^), 3.60–3.65 (m, 1H, CH^pip^), 3.73 (s, 2H, C_6_H_5_CH_2_C(O)), 3.88–3.93 (m, 1H, CH^pip^), 7.21–7.24 (m, 3H, C_6_H_5_), 7.29–7.32 ppm (m, 2H, C_6_H_5_); ^13^C NMR (150 MHz, CDCl_3_): δ = 12.09 (NCH_2_CH_2_CH_3_), 20.40 (NCH_2_CH_2_CH_3_), 24.40 (C-3), 30.17 (C-4), 30.84 (C-2), 31.65 (CH_2_^pip^), 39.32 (CH_2_^pip^), 41.39 (C_6_H_5_CH_2_C(O)), 43.65 (CH_3_), 57.83 (C-5), 59.92 (NCH_2_CH_2_CH_3_), 68.25 (C-1), 73.98 (CH^pip^), 126.93, 128.75, 128.92, 135.52 (C_6_H_5_), 169.50 ppm (C=O).

*1-{4-{{5-[Methyl(propyl)amino]pentyl}oxy}piperidin-1-yl}-3-phenylpropan-1-one* (**5b**): (815 mg, 84.0%): R_f_ = 0.45 (CH_2_Cl_2_/MeOH/NH_3(aq_ 8:1:1%_)_); ^1^H NMR (600 MHz, CDCl_3_): δ = 0.88 (t, *J* = 7.4 Hz, 3H, NCH_2_CH_2_CH_3_), 1.33–1.37 (m, 2H, H-3), 1.45–1.53 (m, 6H, H-4, NCH_2_CH_2_CH_3_, CH_2_^pip^), 1.54–1.60 (m, 2H, H-2), 1.69–1.72 (m, 1H, CH^pip^), 1.76–1.80 (m, 1H, CH^pip^), 2.21 (s, 3H, CH_3_), 2.27–2.34 (m, 4H, H-5, NCH_2_CH_2_CH_3_), 2.62 (t, *J* = 8.2 Hz, 2H, C_6_H_5_CH_2_CH_2_C(O)), 2.96 (t, *J* = 7.7 Hz, 2H, C_6_H_5_CH_2_CH_2_C(O)), 3.15–3.18 (s, 1H, CH^pip^), 3.30–3.34 (s, 1H, CH^pip^), 3.40–3.48 (m, 3H, H-1, CH^pip^), 3.57–3.61 (m, 1H, CH^pip^), 3.88–3.92 (m, 1H, CH^pip^), 7.18–7.22 (m, 3H, C_6_H_5_), 7.26–7.29 ppm (m, 2H, C_6_H_5_); ^13^C NMR (150 MHz, CDCl_3_): δ = 12.15 (NCH_2_CH_2_CH_3_), 20.61 (NCH_2_CH_2_CH_3_), 24.46 (C-3), 30.25 (C-2 and C-4), 30.91 (CH_2_^pip^), 31.88 (C_6_H_5_CH_2_CH_2_C(O)), 35.33 (C_6_H_5_CH_2_CH_2_C(O)), 29.19 (CH_2_^pip^), 43.03 (CH_3_), 57.98 (C-5), 60.09 (NCH_2_CH_2_CH_3_), 68.28 (C-1), 74.03 (CH^pip^), 126.36, 128.65, 128.72 (C_6_H_5_), 141.66 (C_6_H_5_), 170.72 ppm (C=O).

*N-Methyl-N-{3-{{5-[methyl(propyl)amino]pentyl}oxy}propyl}-2-phenylacetamide* (**7a**): (382 mg, 42.0%): R_f_ = 0.48 (CH_2_Cl_2_/MeOH/NH_3(aq)_ 8:1:1%); ^1^H NMR (600 MHz, CDCl_3_): δ = 0.86 (t, *J* = 7.4 Hz, 3H, NCH_2_CH_2_CH_3_), 1.29–1.38 (m, 2H, H-6), 1.47–1.52 (m, 4H, H-5, NCH_2_CH_2_CH_3_), 1.55–1.62 (m, 2H, H-7), 1.72–1.76 (m, 1H, H-2), 1.77–1.82 (m, 1H, H-2), 2.22 (s, 1.5H, CH_3_), 2.23 (s, 1.5H, CH_3_), 2.29–2.37 (m, 4H, H-8, NCH_2_CH_2_CH_3_), 2.92 (s, 1.5H, CH_3_), 2.97 (s, 1.5H, CH_3_), 3.34–3.46 (m, 6H, H-1, H-3, H-4), 3.69 (s, 1H, C_6_H_5_CH_2_C(O)), 3.74 (s, 1H, C_6_H_5_CH_2_C(O)), 7.20–7.32 ppm (m, 5H, C_6_H_5_); ^13^C NMR (150 MHz, CDCl_3_): δ = 11.74 and 11.78 (NCH_2_CH_2_CH_3_), 19.39 and 19.58 (NCH_2_CH_2_CH_3_), 24.07 and 24.13 (C-6), 25.90 and 26.25 (C-7), 27.67 and 28.56 (C-2), 29.49 and 29.63 (C-5), 33.35 and 36.18 (CH_3_), 40.48 and 41.37 (CH_3_), 41.43 and 41.61 (C_6_H_5_CH_2_C(O)), 45.59 and 47.21 (C-1), 57.09 and 57.23 (C-8), 59.02 and 59.23 (NCH_2_CH_2_CH_3_), 67.17 and 68.35 (C-3), 70.73 and 70.93 (C-4), 126.71, 126.74, 128.63, 128.67, 128.82, 128.89, 129.01, 129.29, 135.19, 135.63 (C_6_H_5_), 170. 91 and 171.20 ppm (C=O).

*N-Methyl-N-{3-{{5-[methyl(propyl)amino]pentyl}oxy}propyl}-3-phenylpropanamide* (**7b**): (705 mg, 75.0%): R_f_ = 0.70 (CH_2_Cl_2_/MeOH/NH_3(aq)_ 8:1:1%); ^1^H NMR (600 MHz, CDCl_3_): δ = 0.88 (t, *J* = 7.3 Hz, 3H, NCH_2_CH_2_CH_3_), 1.26–1.36 (m, 2H, H-6), 1.42–1.59 (m, 6H, H-5, H-7, NCH_2_CH_2_CH_3_), 1.72–1.80 (m, 2H, H-2), 2.19 (s, 3H, CH_3_), 2.25–2.33 (m, 4H, H-8, NCH_2_CH_2_CH_3_), 2.59 (t, *J* = 7.7 Hz, 1H, C_6_H_5_CH_2_CH_2_C(O)), 2.65 (t, *J* = 7.7 Hz, 1H, C_6_H_5_CH_2_CH_2_C(O)), 2.91 (s, 3H, CH_3_), 2.97 (q, *J* = 6.7 Hz, 2H, C_6_H_5_CH_2_CH_2_C(O)), 3.32–3.36 (m, 3H, H-1, H-3), 3.37–3.41 (m, 2H, H-4), 3.43 (t, *J* = 6.9 Hz, 1H, H-3), 7.17–7.28 ppm (m, 5H, C_6_H_5_); ^13^C NMR (150 MHz, CDCl_3_): δ = 12.14 (NCH_2_CH_2_CH_3_), 20.61 (NCH_2_CH_2_CH_3_), 24.37 and 24.42 (C-6), 27.34 (C-7), 27.98 and 28.82 (C-2), 29.87 and 29.92 (C-5), 31.56 and 31.83 (C_6_H_5_CH_2_CH_2_C(O)), 34.94 (CH_3_), 35.70 and 35.89 (C_6_H_5_CH_2_CH_2_C(O)), 42.47 (CH_3_), 45.69 and 46.88 (C-1), 57.94 and 57.96 (C-8), 60.07 and 60.09 (NCH_2_CH_2_CH_3_), 67.26 and 68.59 (C-3), 71.17 and 71.29 (C-4), 126.25, 126.28, 128.62, 128.65, 141,76, 141.80 (C_6_H_5_), 172.19 and 172.50 ppm (C=O).

#### 3.1.4. General Procedure for the Preparation of Compounds **1e**,**f** and **2e**,**f**

To a vigorous stirred solution of the corresponding amides **5a**,**b** or **7a**,**b** (1.0 mmol) in 250 mL of anhydrous diethyl ether, LiAlH_4_ (2.5 mmol) was added portionwise. The mixture was stirred at reflux for 2 h, cooled to room temperature, and quenched by a dropwise addition of water (0.5 mL). The suspension was stirred for 30 min and filtered. The filter cake was washed with diethyl ether. The solvent was evaporated, and the residue was purified by silica gel-flash column chromatography (eluent: CH_2_Cl_2_/MeOH/NH_3(aq)_ 8:1:1%) to yield a sticky oil.

*N-Methyl-5-[(1-phenethylpiperidin-4-yl)oxy]-N-propylpentan-1-amine* (**1e**): (252 mg, 72.0%): R_f_ = 0.32 (CH_2_Cl_2_/MeOH/NH_3(aq)_ 8:1:1%) = 0.32; ^1^H NMR (600 MHz, CDCl_3_): δ = 0.89 (t, *J* = 7.3 Hz, 3H, NCH_2_CH_2_CH_3_), 1.32–1.37 (m, 2H, H-3), 1.45–1.51 (m, 4H, H-4, NCH_2_CH_2_CH_3_), 1.56–1.65 (m, 4H, H-2, CH_2_^pip^), 1.87–1.92 (m, 2H, CH_2_^pip^), 2.18–2.22 (m, 5H, CH_3_, CH_2_^pip^), 2.28 (t, *J* = 7.4 Hz, 2H, NCH_2_CH_2_CH_3_), 2.32 (t, *J* = 7.3 Hz, 2H, H-5), 2.57 (t, *J* = 8.6 Hz, 2H, C_6_H_5_CH_2_CH_2_), 2.78–2.84 (m, 4H, C_6_H_5_CH_2_CH_2_, CH_2_^pip^), 3.26–3.31 (m, 1H, CH^pip^), 3.43 (t, *J* = 6.6 Hz, 2H, H-1), 7.17–7.19 (m, 3H, C_6_H_5_), 7.25–7.28 ppm (m, 2H, C_6_H_5_); ^13^C NMR (150 MHz, CDCl_3_): δ = 12.17 (NCH_2_CH_2_CH_3_), 20.65 (NCH_2_CH_2_CH_3_), 24.49 (C-3), 27.37 (C-4). 30.32 (C-2), 31.67 (CH_2_^pip^), 34.16 (C_6_H_5_CH_2_CH_2_), 42.54 (CH_3_), 51.53 (CH_2_^pip^), 58.03 (C-5), 60.12 (C_6_H_5_CH_2_CH_2_), 60.77 (NCH_2_CH_2_CH_3_), 68.02 (C-1), 75.01 (CH^pip^), 126.19, 128.57, 128.92, 140.80 ppm (C_6_H_5_).

Anal. calcd for dihydrogenoxolate (C_22_H_38_N_2_O 2C_2_H_2_O_4_): C 59.30, H 8.04, N 5.32; found: C 58.86, H 8.44, N 5.38; mp_dihydrogenoxolate_= 131.0–133.0 °C.

*N-Methyl-5-{[1-(3-phenylpropyl)piperidin-4-yl]oxy}-N-propylpentan-1-amine* (**1f**): (342 mg, 95.0%): R_f_ = 0.45 (CH_2_Cl_2_/MeOH/NH_3(aq)_ 8:1:1% CH_2_Cl_2_/MeOH/NH_3(aq)_); ^1^H NMR (600 MHz, CDCl_3_): δ = 0.88 (t, *J* = 7.3 Hz, 2H, NCH_2_CH_2_CH_3_), 1.31–1.36 (m, 2H, H-3), 1.44–1.50 (m, 4H, H-4, NCH_2_CH_2_CH_3_), 1.55–1.61 (m, 4H, H-2, CH_2_^pip^), 1.81 (t, *J* = 7.6 Hz, 2H, C_6_H_5_CH_2_CH_2_CH_2_), 1.85–1.88 (m, 2H, CH_2_^pip^), 2.07–2.11 (m, 2H, CH_2_^pip^), 2.20 (s, 3H, CH_3_), 2.27 (t, *J* = 7.6 Hz, 2H, H-5), 2.30–2.35 (m, 4H, NCH_2_CH_2_CH_3_, C_6_H_5_CH_2_CH_2_CH_2_), 2.62 (t, *J* = 7.6 Hz, 2H, C_6_H_5_CH_2_CH_2_CH_2_), 2.72–2.76 (m, 2H, CH_2_^pip^), 3.23–3.28 (m, 1H, CH^pip^), 3.41 (t, *J* = 6.6 Hz, 2H, H-1), 7.15–7.18 (m, 3H, C_6_H_5_), 7.25–7.28 ppm (m, 2H, C_6_H_5_); ^13^C NMR (150 MHz, CDCl_3_): δ = 12.16 (NCH_2_CH_2_CH_3_), 20.66 (NCH_2_CH_2_CH_3_), 24.47 (C-3), 27.37 (C-4), 29.11 (C_6_H_5_CH_2_CH_2_CH_2_), 30.31 (C-2), 31.68 (CH_2_^pip^), 34.02 (C_6_H_5_CH_2_CH_2_CH_2_), 42.53 (CH_3_), 51.57 (CH_2_^pip^), 58.03 (C_6_H_5_CH_2_CH_2_CH_2_), 58.21 (NCH_2_CH_2_CH_3_), 60.12 (C-5), 67.98 (C-1), 75.28 (CH^pip^), 125.91, 128.48, 128.61, 142.49 ppm (C_6_H_5_).

Anal. calcd for dihydrogenoxolate (C_23_H_40_N_2_O 2C_2_H_2_O_4_ 0.5 H_2_O): C 59.00, H 8.25, N 5.10; found: C 58.90, H 8.42, N 5.20; mp_dihydrogenoxolate_ = 134.5–137.8 °C. 

*N-Methyl-5-{3-[methyl(phenethyl)amino]propoxy}-N-propylpentan-1-amine* (**2e**): (264 mg, 80.0%): R_f_ = 0.48 (CH_2_Cl_2_/MeOH/NH_3(aq)_ 8:1:1%); ^1^H NMR (600 MHz, CDCl_3_): δ = 0.88 (t, *J* = 7.4 Hz, 3H, NCH_2_CH_2_CH_3_), 1.31–1.36 (m, 2H, H-6), 1.45–1.49 (m, 4H, H-7, NCH_2_CH_2_CH_3_), 1.55–1.59 (m, 2H, H-5), 1.72–1.77 (m, 2H, H-2), 2.19 (s, 3H, CH_3_), 2.58–2.32 (m, 7H, H-8, NCH_2_CH_2_CH_3_, CH_3_), 2.48 (t, *J* = 7.3 Hz, 2H, H-1), 2.59–2.62 (m, 2H, C_6_H_5_CH_2_CH_2_), 2.75–2.78 (m, 2H, C_6_H_5_CH_2_CH_2_), 3.39 (t, *J* = 6.7 Hz, 2H, H-4), 3.42 (t, *J* = 6.5 Hz, 2H, H-3), 7.18–7.28 ppm (m, 5H, C_6_H_5_); ^13^C NMR (150 MHz, CDCl_3_): δ = 12.18 (NCH_2_CH_2_CH_3_), 20.67 (NCH_2_CH_2_CH_3_), 24.46 (C-6), 27.40 (C-2), 2.89 (C-7), 29.96 (C-5), 34.08 (C_6_H_5_CH_2_CH_2_), 42.47 and 42.53 (2× CH_3_), 54.66 (C-1), 58.03 (C_6_H_5_CH_2_CH_2_), 59.86 (C-8), 60.13 (NCH_2_CH_2_CH_3_), 69.36 (C-3), 71.14 (C-4), 126.14, 128.55, 128.92, 140.88 ppm (C_6_H_5_).

Anal. calcd for dihydrogenoxolate (C_21_H_38_N_2_O 2C_2_H_2_O_4_ 0.5 H_2_O): C 57.34, H 8.28, N 5.35; found: C 57.32, H 8.53, N 5.40; mp_dihydrogenoxolate_ = 110.0–112.0 °C.

*N-Methyl-5-(3-(methyl(3-phenylpropyl)amino)propoxy)-N-propylpentan-1-amine* (**2f**): (175 mg, 50.0%): R_f_ = 0.63 (CH_2_Cl_2_/MeOH/NH_3(aq)_ 8:1:1%) = 0.63. ^1^H NMR (600 MHz, CDCl_3_): δ = 0.88 (t, *J* = 7.4 Hz, 3H, NCH_2_CH_2_CH_3_), 1.30–1.36 (m, 2H, H-6), 1.43–1.49 (m, 4H, H-7, NCH_2_CH_2_CH_3_), 1.55–1.60 (m, 2H, H-5), 1.69–1.72 (m, 2H, H-2), 1.76–1.80 (m, 2H, C_6_H_5_CH_2_CH_2_CH_2_), 2.19 (s, 3H, CH_3_), 2.20 (s, 3H, CH_3_), 2.26 (t, *J* = 7.6 Hz, 2H, NCH_2_CH_2_CH_3_), 2.30 (t, *J* = 7.5 Hz, 2H, H-8), 2.36 (t, *J* = 7.3 Hz, 2H, H-1), 2.39 (t, *J* = 7.1 Hz, 2H, C_6_H_5_CH_2_CH_2_CH_2_), 2.62 (t, *J* = 7.7 Hz, 2H, C_6_H_5_CH_2_CH_2_CH_2_), 3.39 (t, *J* = 6.7 Hz, 2H, H-3), 3.43 (t, *J* = 6.5 Hz, 2H, H-4), 7.15–7.18 (m, 2H, C_6_H_5_), 7.25–7.27 ppm (m, 3H, C_6_H_5_); ^13^C NMR (150 MHz, CDCl_3_): δ = 12.17 (NCH_2_CH_2_CH_3_), 20.71 (NCH_2_CH_2_CH_3_), 24.44 (C-6), 27.44 (C-7), 27.88 (C-2), 29.31 (C_6_H_5_CH_2_CH_2_CH_2_), 29.96 (C-5), 33.94 (C_6_H_5_CH_2_CH_2_CH_2_), 42.44 and 42.55 (2× CH3), 54.80 (C_6_H_5_CH_2_CH_2_CH_2_), 57.53 (C-1), 58.05 (C-8), 60.16 (NCH_2_CH_2_CH_3_), 69.44 (C-4), 71.14 (C-3), 125.89, 128.49, 128.61, 142.64 ppm (C_6_H_5_).

Anal. calcd for dihydrogenoxolate (C_22_H_40_N_2_O 2C_2_H_2_O_4_): C 59.07, H 8.39, N 5.30; found: C 58.84, H 8.80, N 5.39; mp_dihydrogenoxolate_ = 104.5–107.0 °C.

^1^H and ^13^C NMR spectral data of final compounds **1b**,**c**,**e**,**f** and **2b**,**c**,**e**,**f** can be found in Supplementary Material A (Section 3).

### 3.2. In Vitro Pharmacology

#### 3.2.1. H_3_ Antagonistic Activity for Compounds **1a**–**f** and **2a**–**f**

In the first step, all the obtained compounds were tested for their H_3_ antagonistic effects in vitro, following standard methods, using the electrically contracting guinea pig jejunum [32].

Male guinea pigs weighing 300–400 g were sacrificed, and a portion of the small intestine, 20–50 cm proximal to the ileocaecal valve (jejunum), was removed and placed in Krebs buffer (composition (mM) NaCl 118; KCl 5.6; MgSO_4_ 1.18; CaCl_2_ 2.5; NaH_2_PO_4_ 1.28; NaHCO_3_ 25; glucose 5.5; indomethacin (1 × 10^ℒ6^mol/L)). Whole jejunum segments (2 cm) were prepared and mounted between two platinum electrodes (4 mm apart) in 20 mL Krebs buffer, continuously gassed with 95% O_2_:5% CO_2_, and maintained at 37 °C. the contractions were recorded isotonically under 1.0 g tension with a Hugo Sachs Hebel–Messvorsatz (Tl-2)/HF-modem (Hugo Sachs Elektronik, Hugstetten, Germany) connected to a pen recorder. The equilibration lasted for one hour with washings every 10 min. The muscle segments were then stimulated at a maximum between 15 and 20 Volts, continuously at a frequency of 0.1 Hz for a duration of 0.5 msec, with rectangular wave electrical pulses, delivered by a Grass Stimulator S-88 (Grass Instruments Co., Quincy, MA, USA). After 30 min of stimulation and 5 minutes before adding (*R*)-*α*-methylhistamine, pyrilamine (1 × 10^ℒ5^mol/L concentration in organ bath) was added, and then cumulative concentration–response curves (half-log increments) of (*R*)-*α*-methylhistamine, an H_3_-agonist, were recorded until no further change in the responses was found. Five minutes before adding the tested compounds, the pyrilamine (1 × 10^ℒ5^mol/L concentration in an organ bath) was added. The antagonists were preincubated for 20 min during the stimulation, before the preparation were challenged with (*R*)-*α*-methylhistamine. Antagonist potency was determined by the construction of a Schild plot [34], using three different concentrations of the antagonist. The potency of an antagonist is expressed by its pA_2_ value. The pA_2_ values were compared with those indicating the potency of thioperamide.

#### 3.2.2. H_1_ Antagonistic Activity for Compounds **1b** and **1f**

In addition, the compounds **1b** and **1f** with the highest potency at the H_3_ receptors were also tested for H_1_ antagonistic effects in vitro, using standard methods [34]. The potencies of the aforementioned ligands were determined for the guinea pig ileum histamine H_3_ receptors as described previously [36], using histamine as a competing agonist.

The pA_2_ values were calculated according to Arunlakshana and Schild [34]. The pA_2_ values were compared with those of pyrilamine.

##### Chemicals

Thioperamide maleate, (*R*)-*α*-methylhistamine dihydrochloride, indomethacin, pyrilamine maleate, and histamine dihydrochloride were purchased from Sigma Aldrich (Saint Louis, MO, USA).

#### 3.2.3. Antagonist Binding to Rat rH_3_R and Human hH_3_R

##### Cell Culture and Transfection

Human Embryonic Kidney cells (HEK293T) were cultured in DMEM supplemented with 10% Fetal Bovine Serum, 100 IU·mL^−1^ penicillin, and 100 μg·mL^−1^ streptomycin at 37 °C and 5% CO_2_. The day prior to transfection, 2 million cells were seeded in 10 cm dishes. Approximately 4 million cells were transfected by the polyethyleneimine (PEI) method with 5 μg of cDNA in a ratio of 1:4 (DNA/PEI). Briefly, 0.5 μg of pcDNA3-rH_3_R or pcDNA3.1-hH_3_R and 4.5 μg of empty plasmid (pcDNA3.1) were mixed with 20 μg of 25 kDa linear PEI in 500 μL of 150 mM NaCl and incubated for 30 min at 22 °C. Meanwhile, the medium in the 10 cm dishes was replaced with fresh culture medium, and the transfection mix was subsequently added dropwise to the cells, which were incubated for 48 h at 37 °C and 5% CO_2_.

##### Crude Membrane Extracts

Forty-eight hours after transfection, the cells were washed with ice-cold phosphate buffered saline (PBS) and scrapped, and the homogenate was centrifuged for 10 min at ~2000× *g*, 4 °C. The supernatant was aspirated, and the cell pellets were resuspended in 1 ml ice-cold PBS and centrifuged again under the same conditions, aspirating the supernatant. The membranes were stored at −20 °C until further use. 

##### [^3^H]-*N*^α^-Methylhistamine Binding

The affinities of the derivative **ADS-003** were determined for rat and human histamine H_3_ receptors (445 isoforms) as described previously [36], using [^3^H]-NαMH as a competing radioligand.

##### Chemicals

Dubelcco’s Modified Eagles Medium (DMEM), Phosphate Buffered Saline (PBS), Trizma Base, polyethyleneimine solution (50%, PEI) were purchased from Sigma Aldrich (Saint Louis, MO, USA). Fetal Bovine Serum (FBS, Bodinco BV, Alkmaar, The Netherlands), Penicillin/Streptomycin (streptomycin 10,000 IU·mL^−1^; penicillin 10,000 μg·mL^−1^, Thermo Fischer Scientific, (p/a Perbio Science BVBA, Etten-Leur, The Netherlands) , linear 25 kDa polyethyleneimine (PEI, Polysciences, Warrington, PA, USA), [^3^H]-*N*-α-methylhistamine (specific activity 79.7 Ci/mmol, Perkin Elmer, (Waltham, MA, USA), thioperamide (Abcam, Cambridge, UK) histamine (TCI, Nihonbashi-honcho, Chuo-ku, Tokyo, Japan).

### 3.3. Verification of In Vivo Activity of Compound **ADS-003** (**1a**)

All animal experimental procedures were in accordance with EU directives and local ethical regulations. Male Wistar rats (260–300 g) were used. The animals were maintained under standard laboratory conditions (liquid and food available ad libitum, 12 h light–dark cycle). For feeding behavior examination, the rats were placed individually in metabolic cages (TecniplastGazzada, Buguggiate, Italy) and kept there throughout the entire test. Pharmacotherapy was preceded by a control period of 4 days aimed to determine the basal feed and water consumptions as well as urine excretion. The rats (*n* = 8 per group) were randomly given the H_3_R antagonists **ADS-003** or Ciproxifan, the latter serving as a reference compound [40]. Control rats were treated with an equivalent volume of physiological saline. The compounds, dissolved in distilled water with DMSO (7%, *v*/*v*), were administered subcutaneously at a dose of 3 mg/kg of body mass for 5 consecutive days, always during the morning hours, following the record of feeding parameters. The dose of **ADS-003** had been determined on the basis of a thorough characterization in vitro. The recorded volumes of consumed food are expressed in g or ml per 100 g of body weight or in ml per 24 h, respectively. The final results are given as means, with SEM calculated for each 24 h period, computed from a four-day (before treatment) or a five-day (treatment) monitoring 

#### 3.3.1. Post-Mortem Biochemical Analyses

Following the behavioral study, the rats were sacrificed, and their brains were collected. From each brain, the cerebral cortex and hypothalamus were quickly dissected according to the Glowinski and Iversen method [37]. The samples were immediately frozen in liquid nitrogen and kept at −70 °C until assayed. 

The tissue concentration of histamine (HA) was measured by radioisotopic assay according to Taylor and Snyder [45], while catecholamine (dopamine, DA; noradrenaline, NA) and serotonin (5-HT) concentrations were measured by radioimmunoassays using RIA kits (DIAsourceImmunoAssays S.A., Nivelles, Belgium). The amine concentrations were expressed as ng/g wet weight (HA) or nmol/g wet weight (DA, NA, 5-HT).

Monoamine oxidase A and B (EC 1.4.3.4; MAO A and B) activities were estimated in cerebral homogenates with radioassays, using serotonin (fine conc. 200 μM) and β-phenylethylamine (fine conc. 20 μM), as well as specific inhibitors—clorgyline and deprenyl (0.3 μM each), respectively [46]. Histamine *N*-methyltransferase (EC 2.1.1.8; HMNT) activity was determined radioenzymatically, as described by Taylor and Snyder [45], by measurements of radioactive *N^τ^*-methylhistamine formed in a transmethylation reaction catalyzed by the enzyme, as previously described; *S*-Adenosyl-l-(methyl-^14^C)-Methionine was used as a donor of the methyl group. 

The enzyme activities are expressed as pmol/min/mg protein. Protein concentration was analyzed according to Lowry’s method [47].

##### Chemicals

Adenosyl-l-methionine, S [methyl-^14^C] (specific activity 50 μCi) and β-phenylethylamine hydrochloride [ethyl-1-^14^C] (specific activity 250 μCi) were purchased from ARC.

## 4. Conclusions

The highest in vitro potency as an H_3_ receptor antagonist for both series was observed for the (benzylfuran-2-yl)methyl derivative of 4-hydroxypiperidine **1a** (**ADS-003**) and the benzyl derivative of 3-(methylamino)propan-1-ol **2d**. Compared to **1a** and **2d**, the in vitro potency was found to decrease with increasing chain length (**1a**–**f**; **2d**–**f**), with the exception of the series **2a**–**c**, where only weak potency was observed, independent of the alkyl chain length. 

In competition radioligand binding studies to the rat histamine H_3_ receptor, compound **ADS-003** (pK_i_ = 7.9 ± 0.1) showed nanomolar affinity at the same level as the reference compound thioperamide (pK_i_ = 7.9 ± 0.1) and a slightly higher affinity than histamine (pK_i_ = 7.3 ± 0.1). A significantly lower affinity was observed for **ADS-003** for the human H_3_R, with pK_i_ 6.6 ± 0.1, in comparison with the pK_i_ of thioperamide (7.2 ± 0.1) and the pK_ii_ of histamine (7.7 ± 0.1).

**ADS-003**, given parenterally for 5 days, reduced the food intake of rats as well as changed rats’ brain amine concentrations in a manner and degree similar to those observed for the reference H_3_ antagonist **Ciproxifan**. These results indicate that the compound crosses the blood–brain barrier and acts also as an H_3_R antagonist in vivo in the rat brain.

## Figures and Tables

**Table 1:** H_3_ antagonistic potency of 4-hydroxypiperidine derivatives (**1a**–**f**) and their 3-(methyl-amino)propyloxyanalogues (**2a**–**f**) as tested in the in vitro test system in the guinea pig jejunum.

Cpd.	Code Cpd.	Structure	pA_2_ (sem) ^a^	N (caviae)
**1a** [31]	**ADS-003**	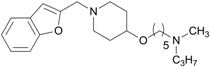	8.27 (0.05) (8.47) *	12 (4)
**1b**	**ADS-013**	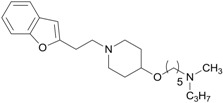	7.26 (0.25)	12 (4)
**1c**	**ADS-014**	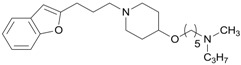	7.11 (0.12)	12 (4)
**1d** [30]	**ADS-009**	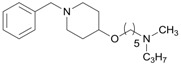	7.79 (0.06)	12 (4)
**1e**	**ADS-015**	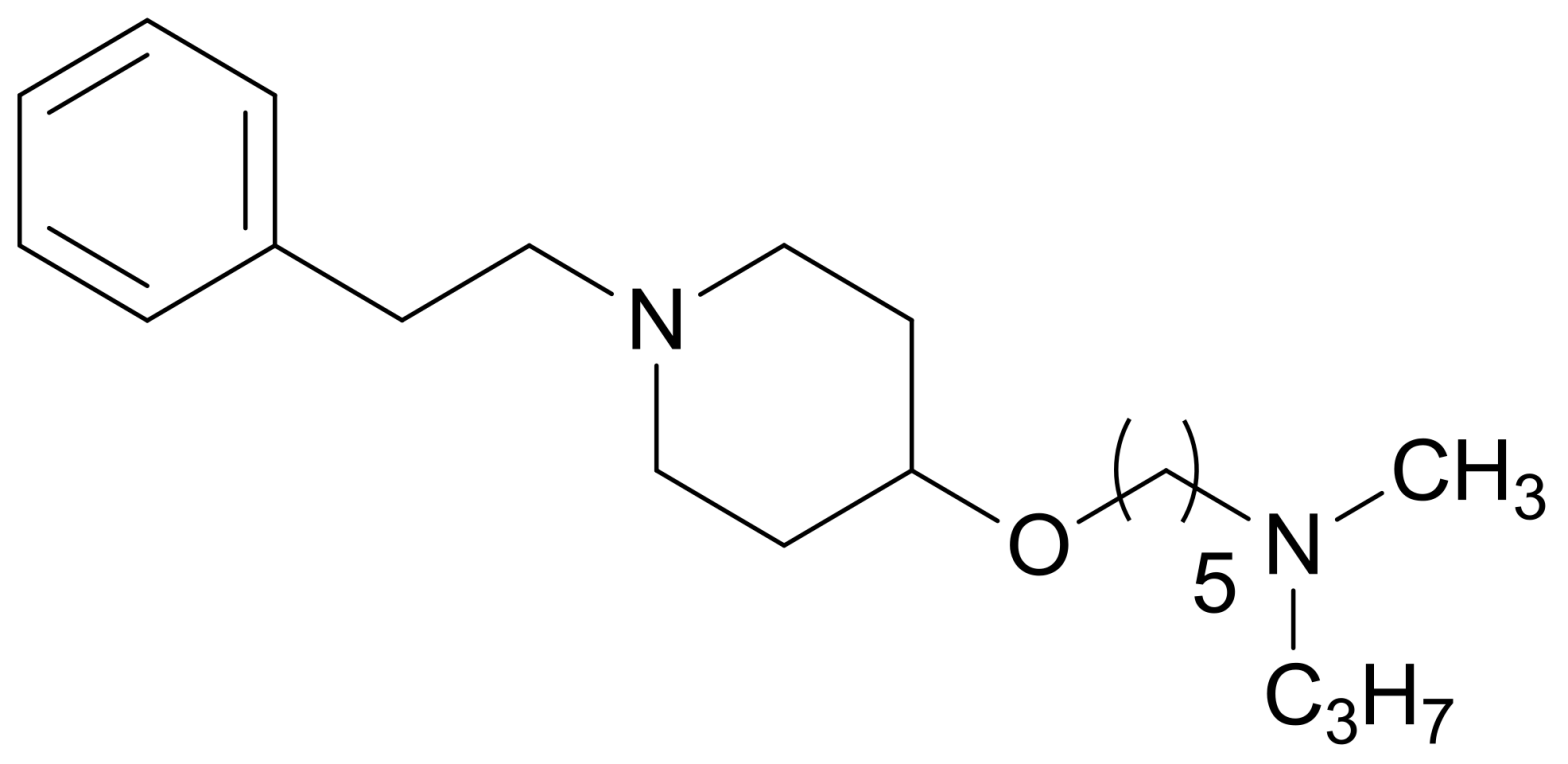	6.67 (0.12)	9 (4)
**1f**	**ADS-016**	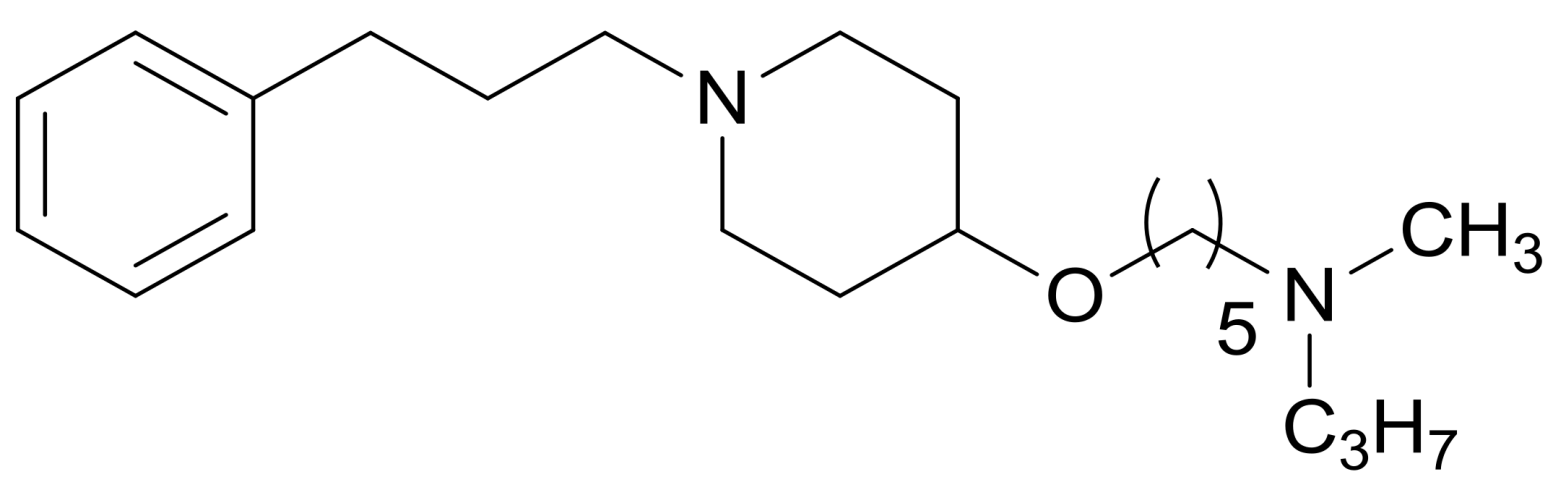	7.51 (0.03)	8 (4)
**2a** [31]	**ADS-012**	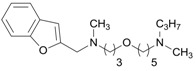	6.23 (0.12)	12 (4)
**2b**	**ADS-017**	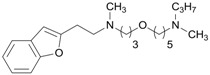	6.32 (0.52)	5 (4)
**2c**	**ADS-018**	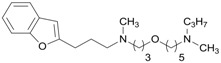	6.37 (0.01)	6 (4)
**2d** [31]	**ADS-011**	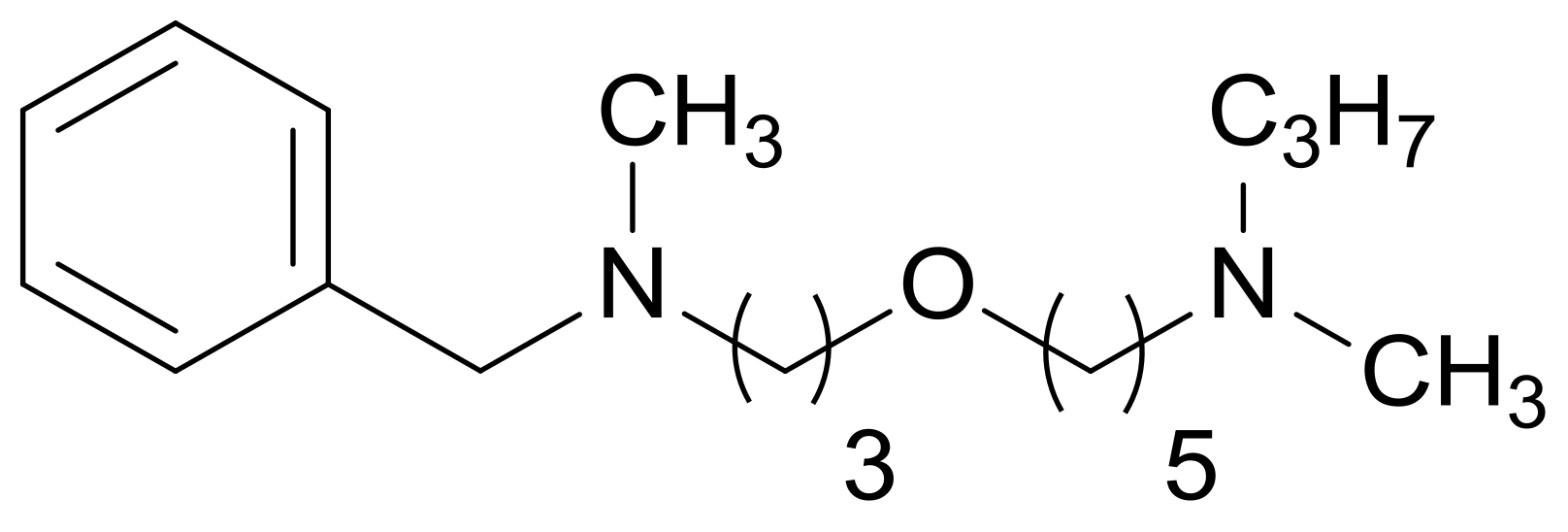	8.06 (0.05)	12 (4)
**2e**	**ADS-019**	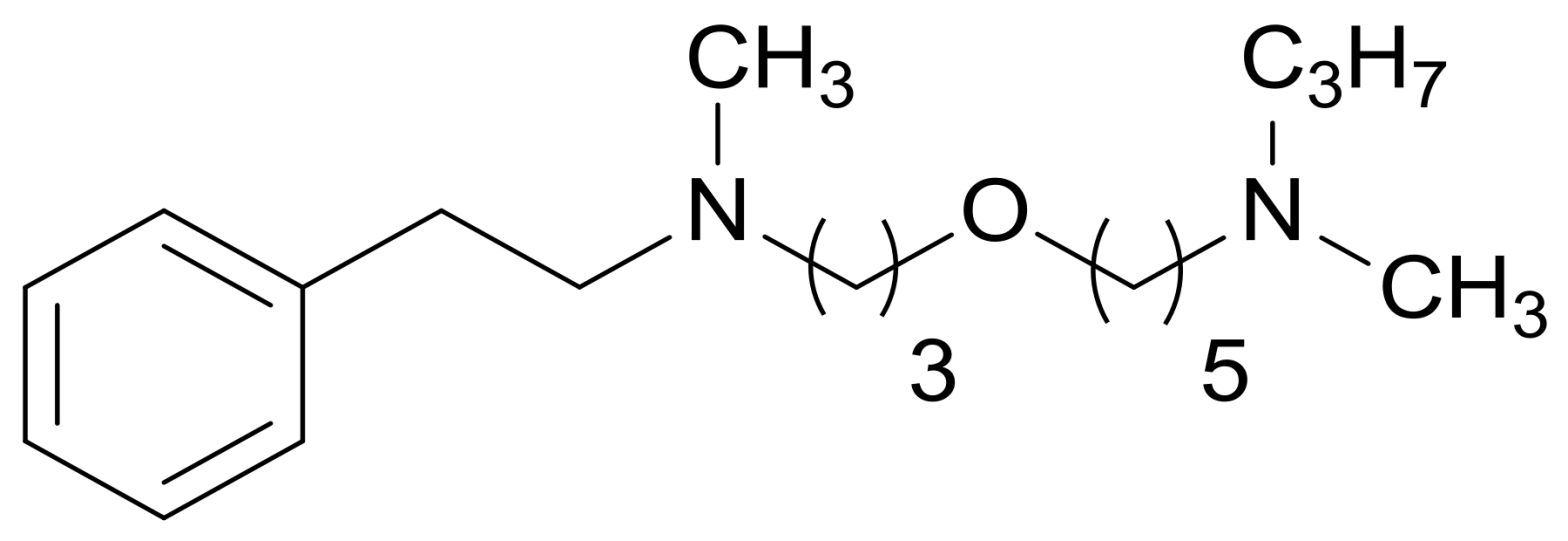	6.72 (0.06)	6 (4)
**2f**	**ADS-020**	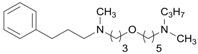	6.79 (0.01)	6 (4)
	Thioperamide		8.44 (0.26)	36 (12)

^a^ sem—standard error of the mean; N—number of different animal preparations; (caviae)—number of animals; * pA_2_ value obtained previously [31]; for Thioperamide *, pA_2_ H_3_ = 8.65, (sem; 0.07); N (caviae)—36 (12).

**Table 2:** The effect of subchronic administration of **ADS-003** (s.c. 3 mg/kg/daily for 5 days) or Ciproxifan (s.c. 3 mg/kg/daily for 5 days) on cerebral MAOs and HNMT activities.

Group	MAO-A	MAO-B	HMNT	
	pmol/min/mg Protein		pmol/min/mg Protein	
	CTX	CTX	CTX	HTH
ADS-003	1536 ± 50	1065 ± 48	44.41 ± 1.06	41.09 ± 0.80
Ciproxifan	1654 ± 31	1049 ± 56	46.90 ± 3.59	51.76 ± 5.67
Control	1506 ± 29	1054 ± 19	45.97 ± 1.28	39.81 ± 1.94

The values are given as means ± SEM for four–eight rats. The drugs were administered subcutaneously (s.c.) at a dose of 3 mg/kg of body mass for 5 consecutive days. MAO—monoamine oxidase, HNMT—histamine *N*-methyltransferase, CTX—cerebral cortex, HTH—hypothalamus. one-way ANOVA and Tukey’s multiple comparisons test showed no statistically significant differences.

## Supplementary Materials

Supplementary materials can be found at http://www.mdpi.com/1422-0067/19/4/1243/s1.
