# Supplementary material for: 4-Hydroxypiperidines and Their Flexible 3-(Amino)propyloxy Analogues as Non-Imidazole Histamine H3 Receptor Antagonist: Further Structure–Activity Relationship Exploration and In Vitro and In Vivo Pharmacological Evaluation
Source: Int J Mol Sci. 2018 Apr 19;19(4):1243. doi: 10.3390/ijms19041243 (PMC5979327; doi:10.3390/ijms19041243)
Supplement: Supplementary file 1 [file ijms-19-01243-s001.pdf]

# Supplementary Material A

**1. Chemical synthesis and analysis data**

*1.1. Compounds 8a,b and 4a,b*

**General procedure for the preparation of compounds 8a,b.**

To a well stirred mixture of 2-iodophenol (4.54 mmol), palladium acetate (0.22 mmol), CuI (0.22 mmol) and triphenylphosphine (0.22 mmol) in dry triethylamine (10.0 ml) 3-butyn-1-ol or 4-pentyn-1-ol (5.0 mmol) was added under argon atmosphere. The reaction mixture was then stirred overnight at room temperature. The reaction mixture was concentrated, diluted with EtOAc (30 mL), and the organic layer was washed with $H_2O$ (2 x 20 mL) and brine (30 mL). The organic layer was dried over $MgSO_4$ and filtered. The solvent was evaporated and the residue was purified by silica gel flash-column chromatography to yield a yellow oil:

**2-(1-benzofuran-2-yl)ethanol 8a**: [Yield: 87.0%][44] (570 mg, 78.0%): $R_f$=0.39 (chloroform/EtOAc 7:3); $^1$H NMR (600 MHz, $CDCl_3$): δ=3.04 (t, *J*=6.1 Hz, 2H, H-1), 3.99 (t, *J*=6.1 Hz, 2H, H-2), 6.51 (s, 1H, $CH^{furan}$), 7.17-7.23 (m, 2H, $C_6H_4$), 7.41 (d, *J*=7.8 Hz, 1H, $C_6H_4$), 7.49 ppm (d, *J*=7.8 Hz, 1H, $C_6H_4$); $^{13}$C NMR (150 MHz, $CDCl_3$): δ=32.33 ($\underline{C}H_2CH_2OH$), 61.00 ($CH_2\underline{C}H_2OH$), 103.91 ($C^{furan}$), 111.08, 120.65, 122.87, 123.77, 128.93, 155.09 ($C_6H_4$), 156.19 ppm ($C^{furan}$).

**3-(1-benzofuran-2-yl)propan-1-ol 8b**: (580 mg, 73.0%): $R_f$=0.40 (chloroform/EtOAc 7:3).; $^1$H NMR (600 MHz, $CDCl_3$): δ=1.97-2.03 (m, 2H, H-2), 2.87 (t, *J*=7.3 Hz, 2H, H-1), 3.72 (t, *J*=6.3 Hz, 2H, H-3), 6.40 (s, 1H, $CH^{furan}$), 7.15-7.21 (m, 2H, $C_6H_4$), 7.39 (d, *J*=7.4 Hz, 1H, $C_6H_4$), 7.46 ppm (d, *J*=7.2 Hz, 1H, $C_6H_4$); $^{13}$C NMR (150 MHz, $CDCl_3$): δ=25.03 ($CH_2\underline{C}H_2CH_2OH$), 30.90 ($\underline{C}H_2CH_2CH_2OH$), 62.21 ($CH_2CH_2\underline{C}H_2OH$), 102.44 ($C^{furan}$), 110.96, 120.47, 122.70, 123.45, 129.12, 154.95 ($C_6H_4$), 157.20 ppm ($C^{furan}$).

**General procedure for the preparation of compounds 4a,b.**

To a solution of the appropriate alcohol **8a,b** in pyridine (6.91 mmol) at 0 °C methane sulfonyl chloride (1.72 mmol) was added dropwise. The reaction mixture was stirred at room temperature overnight and then poured into ice-cold water. The reaction mixture was quenched with hydrochloric acid until slightly acidic pH was noted. The water layer was extracted with diethyl ether (3x20mL). The organic layers were combined, dried over $MgSO_4$, and filtered. The solvent was evaporated and the residue was purified by silica gel flash-column chromatography to give the desired compound as a sticky oil:

**2-(1-benzofuran-2-yl)ethyl methanesulfonate 4a**: (370 mg, 90.0%): $R_f$ =0.81 (chloroform/EtOAc 7:1); $^1H$ NMR (600 MHz, $CDCl_3$): $\delta$=2.94 (s, 3H, $CH_3$), 3.24 (t, *J*=6.6 Hz, 2H, $C\underline{H_2}CH_2OSO_2CH_3$), 4.57 (t, *J*=6.6 Hz, 2H, $CH_2C\underline{H_2}OSO_2CH_3$), 6.55 (s, 1H, $CH^{furan}$), 7.18-7.28 (m, 2H, $C_6H_4$), 7.40-7.42 (m, 1H,$C_6H_4$), 7.49-7.52 ppm (m, 1H, $C_6H_4$); $^{13}C$ NMR (150 MHz, $CDCl_3$): $\delta$=29.11 ($\underline{C}H_2CH_2OSO_2CH_2$), 37.73 ($CH_2\underline{C}H_2OSO_2CH_3$), 67.04 ($CH_3$), 104.67 ($C^{furan}$), 111.12, 120.91, 123.06, 124.18, 128.67, 153.36 ($C_6H_4$), 155.04 ppm ($C^{furan}$).

**3-(1-benzofuran-2-yl)propyl methanesulfonate 4b**: (390 mg, 89.0%): $R_f$ =0.79 (chloroform/EtOAc 7:1); $^1H$ NMR (600 MHz, $CDCl_3$): $\delta$=2.21 (m, 2H, $CH_2C\underline{H_2}CH_2OSO_2CH_3$), 2.94 (t, *J*=7.3 Hz, 2H, $C\underline{H_2}CH_2CH_2OSO_2CH_3$), 2.99 (s, 3H, $CH_3$), 4.30 (t, *J*=6.2 Hz, 2H, $CH_2CH_2C\underline{H_2}OSO_2CH_3$), 6.45 (s, 1H, $CH^{furan}$), 7.17-7.23 (m, 2H, $C_6H_4$), 7.39 (d, *J*=7.9 Hz, 1H, $C_6H_4$), 7.48 ppm (d, *J*=7.5 Hz, 1H, $C_6H_4$); $^{13}C$ NMR (150 MHz, $CDCl_3$): $\delta$=24.65 ($CH_2\underline{C}H_2CH_2OSO_2CH_3$), 27.57 ($\underline{C}H_2CH_2CH_2OSO_2CH_3$), 37.53 ($CH_3$), 68.93 ($CH_2CH_2\underline{C}H_2OSO_2CH_3$), 103.14 ($C^{furan}$), 110.96, 120.62, 122.82, 123.72, 128.86, 154.95 ($C_6H_4$), 157.20 ppm ($C^{furan}$).

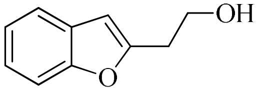


**1.2 $^{1}$H and $^{13}$C NMR spectral data of compounds 4a,b and 8a,b**

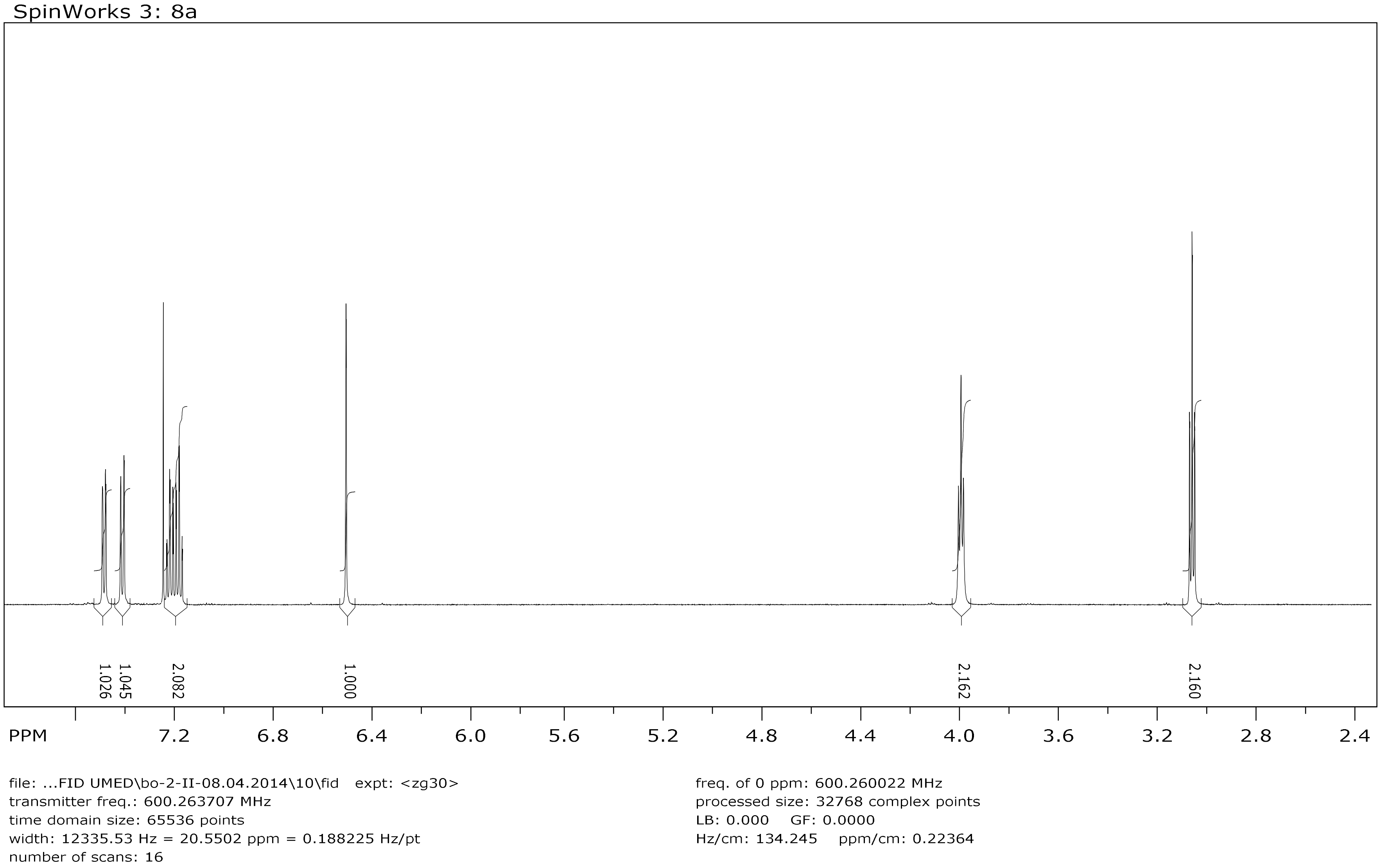

SpinWorks 3: 8a

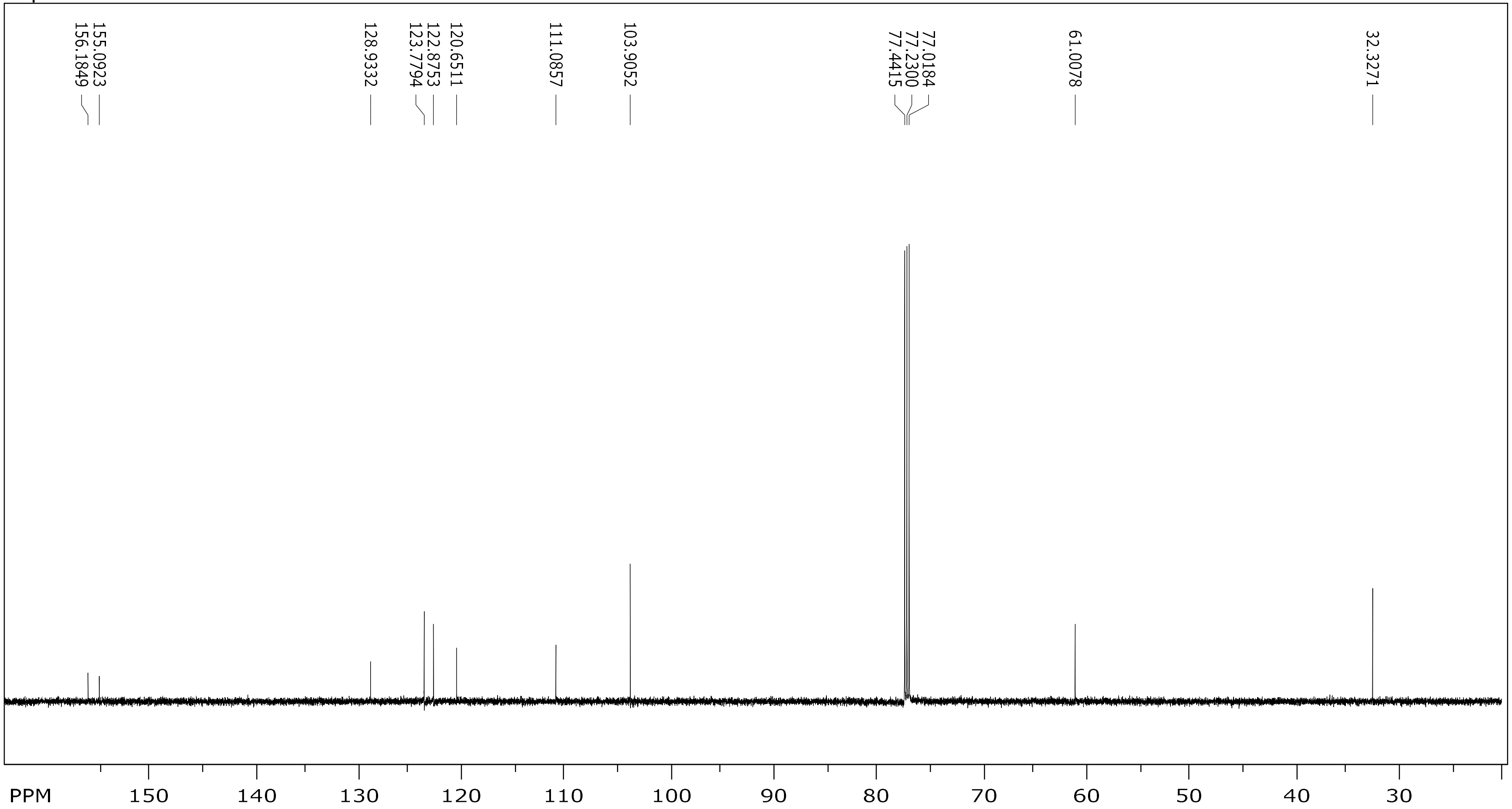


file: ...ataa\Desktop\FID UMED\bo-2-C\1\fid expt: <zgpg30>
transmitter freq.: 150.950591 MHz
time domain size: 65536 points
width: 36057.69 Hz = 238.8708 ppm = 0.550197 Hz/pt
number of scans: 1024

freq. of 0 ppm: 150.935462 MHz
processed size: 32768 complex points
LB: 0.000 GF: 0.0000
Hz/cm: 871.635 ppm/cm: 5.77431

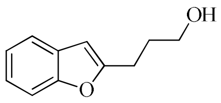


SpinWorks 3: 8b

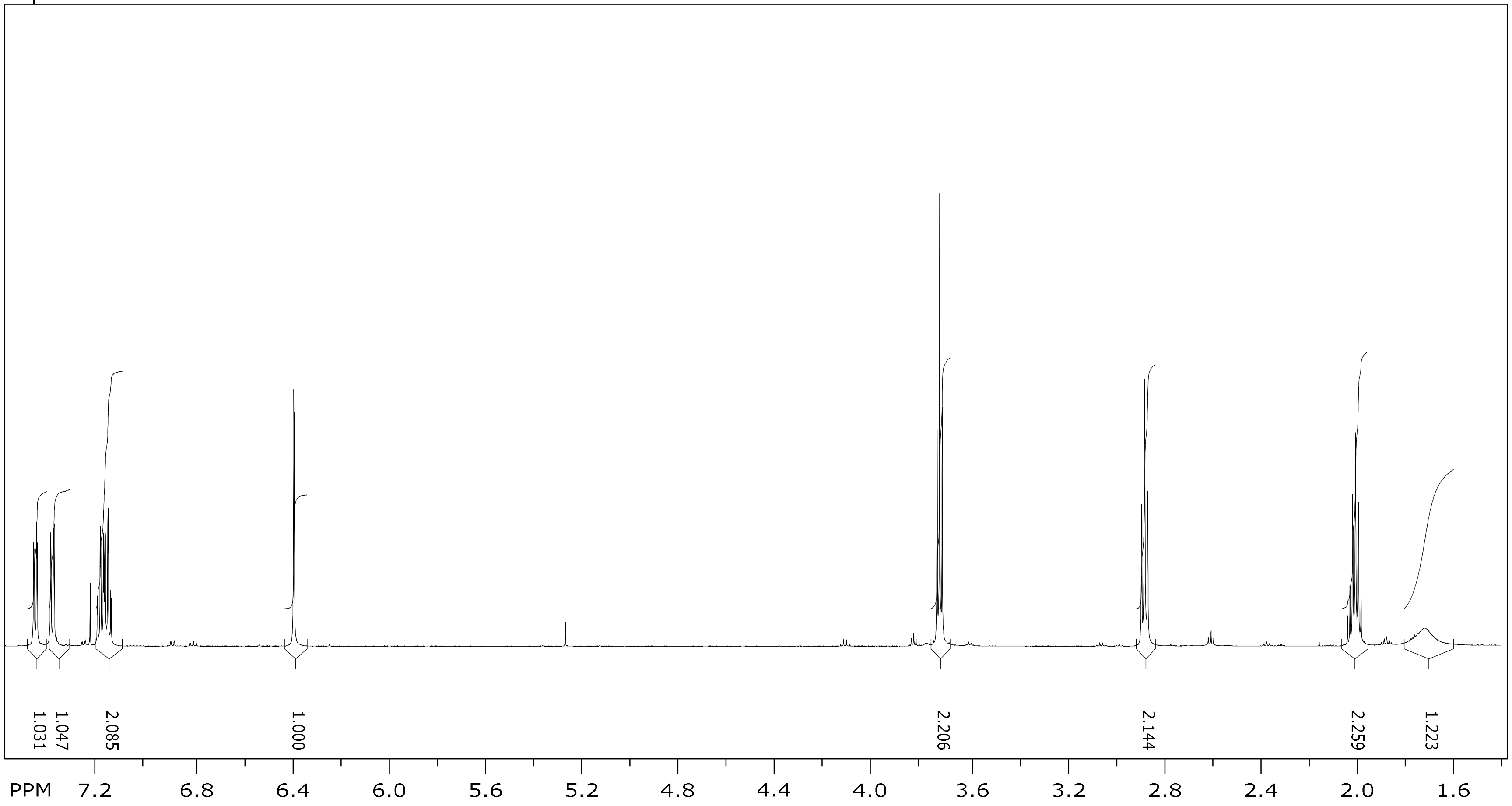


file: ...taa\Desktop\FID UMED\bo-3-I\10\fid   expt: <zg30>
transmitter freq.: 600.263707 MHz
time domain size: 65536 points
width: 12335.53 Hz = 20.5502 ppm = 0.188225 Hz/pt
number of scans: 16

freq. of 0 ppm: 600.260029 MHz
processed size: 32768 complex points
LB: 0.000    GF: 0.0000
Hz/cm: 148.898    ppm/cm: 0.24805

SpinWorks 3: 8b

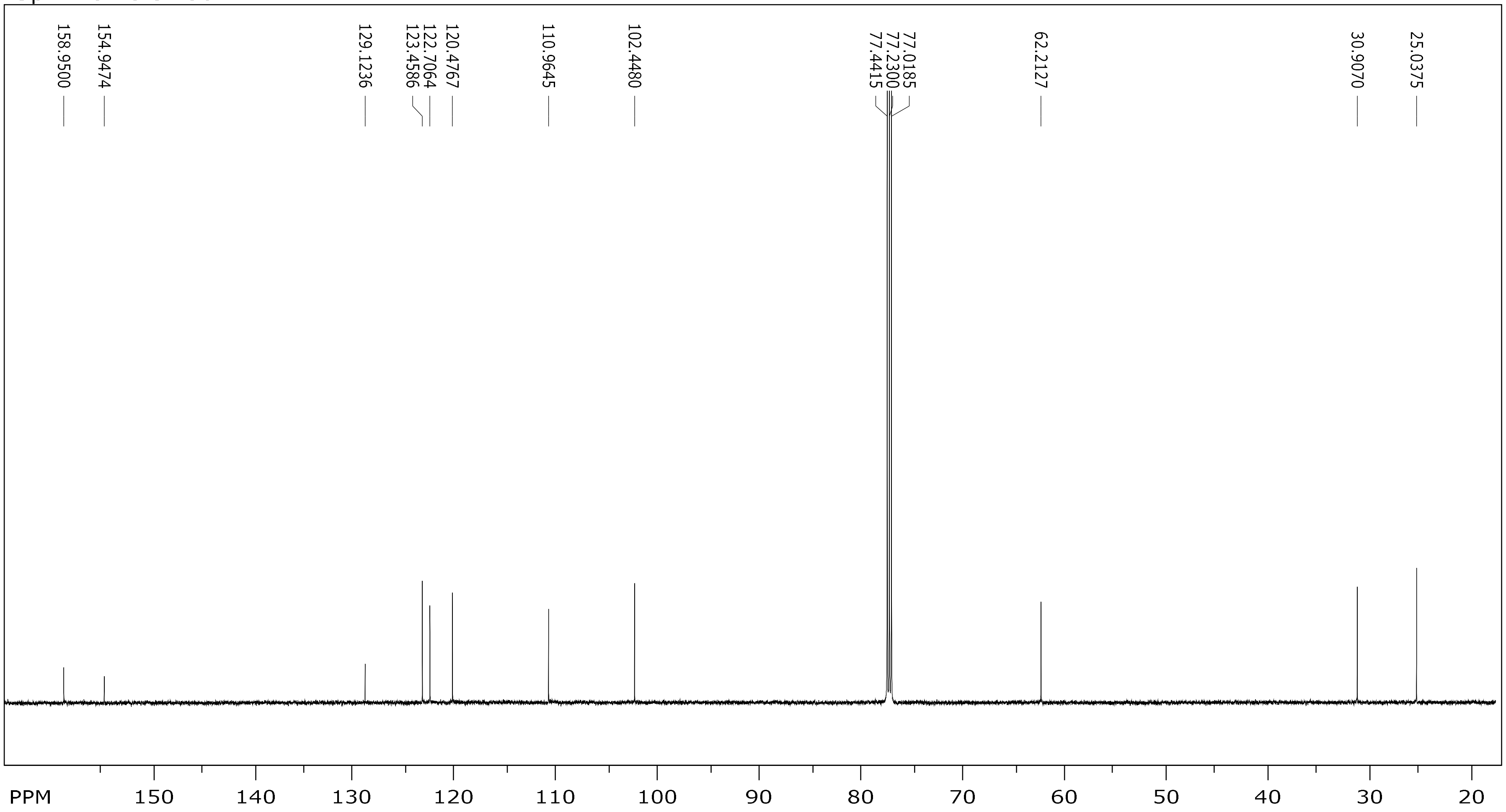


file: ...ataa\Desktop\FID UMED\bo3-c\10\fid expt: <zgpg30>
transmitter freq.: 150.950591 MHz
time domain size: 65536 points
width: 36057.69 Hz = 238.8708 ppm = 0.550197 Hz/pt
number of scans: 3000

freq. of 0 ppm: 150.935461 MHz
processed size: 32768 complex points
LB: 0.000 GF: 0.0000
Hz/cm: 891.314 ppm/cm: 5.90467

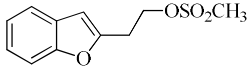


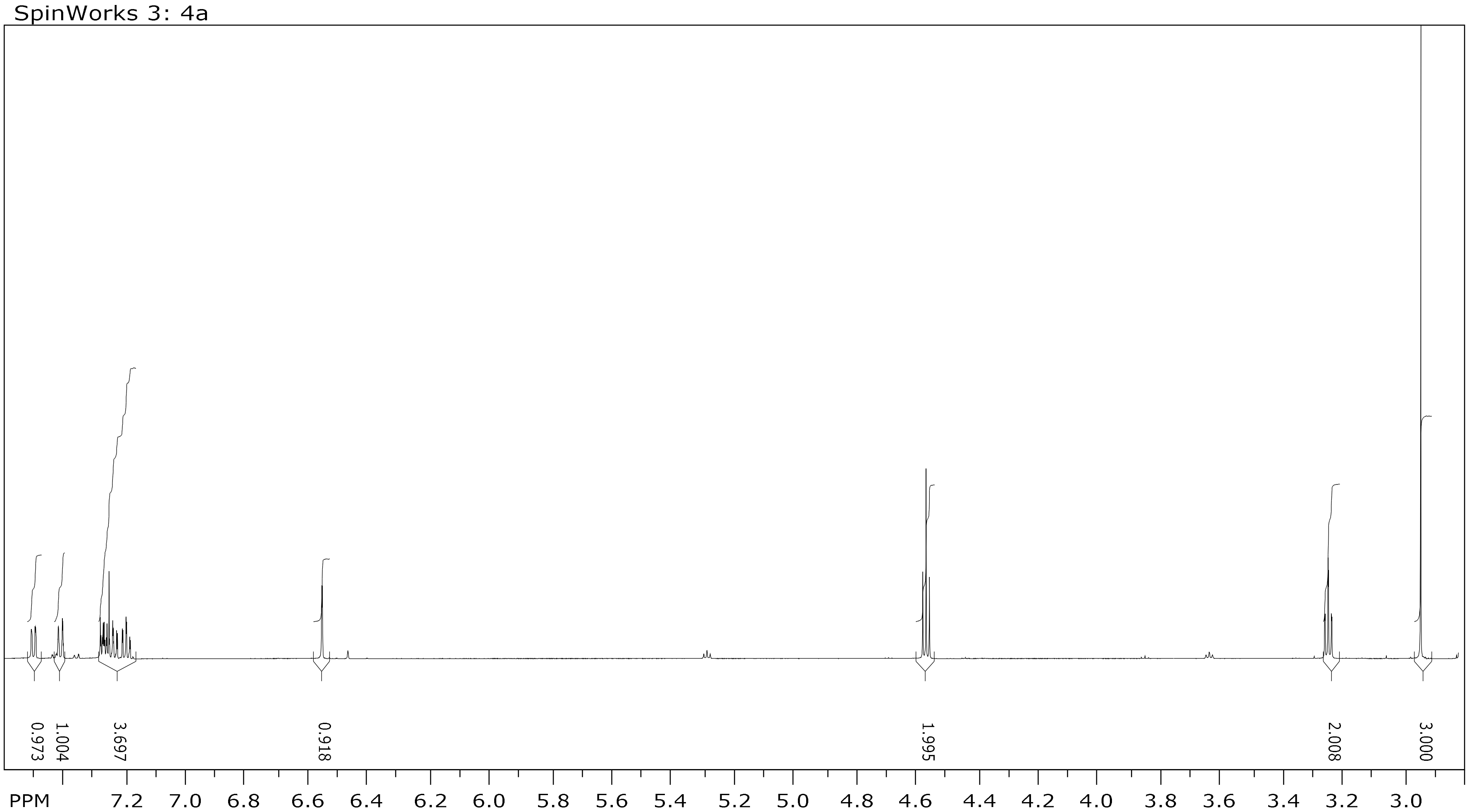


file: ...ktop\FID UMED\bo-32-04.05.15\1\fid expt: <zg30>
transmitter freq.: 600.263707 MHz
time domain size: 65536 points
width: 12335.53 Hz = 20.5502 ppm = 0.188225 Hz/pt
number of scans: 16

freq. of 0 ppm: 600.260019 MHz
processed size: 32768 complex points
LB: 0.000 GF: 0.0000
Hz/cm: 115.081 ppm/cm: 0.19172

SpinWorks 3: 4a

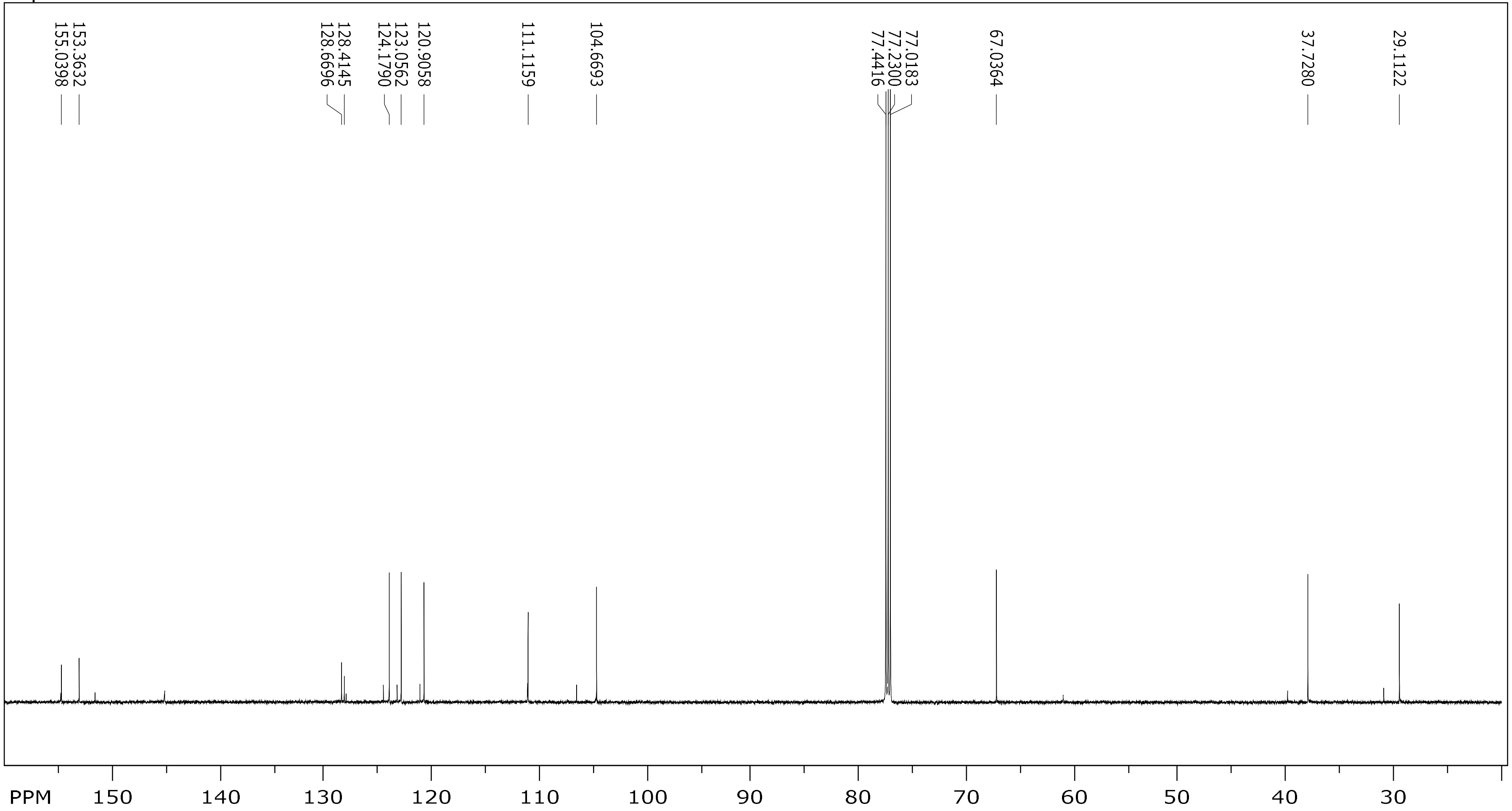


file: ...aa\Desktop\FID UMED\bo-32-C\10\fid expt: <zgpg30>
transmitter freq.: 150.950591 MHz
time domain size: 65536 points
width: 36057.69 Hz = 238.8708 ppm = 0.550197 Hz/pt
number of scans: 3072

freq. of 0 ppm: 150.935464 MHz
processed size: 32768 complex points
LB: 0.000 GF: 0.0000
Hz/cm: 850.799 ppm/cm: 5.63628

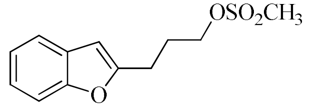

SpinWorks 3: 4b

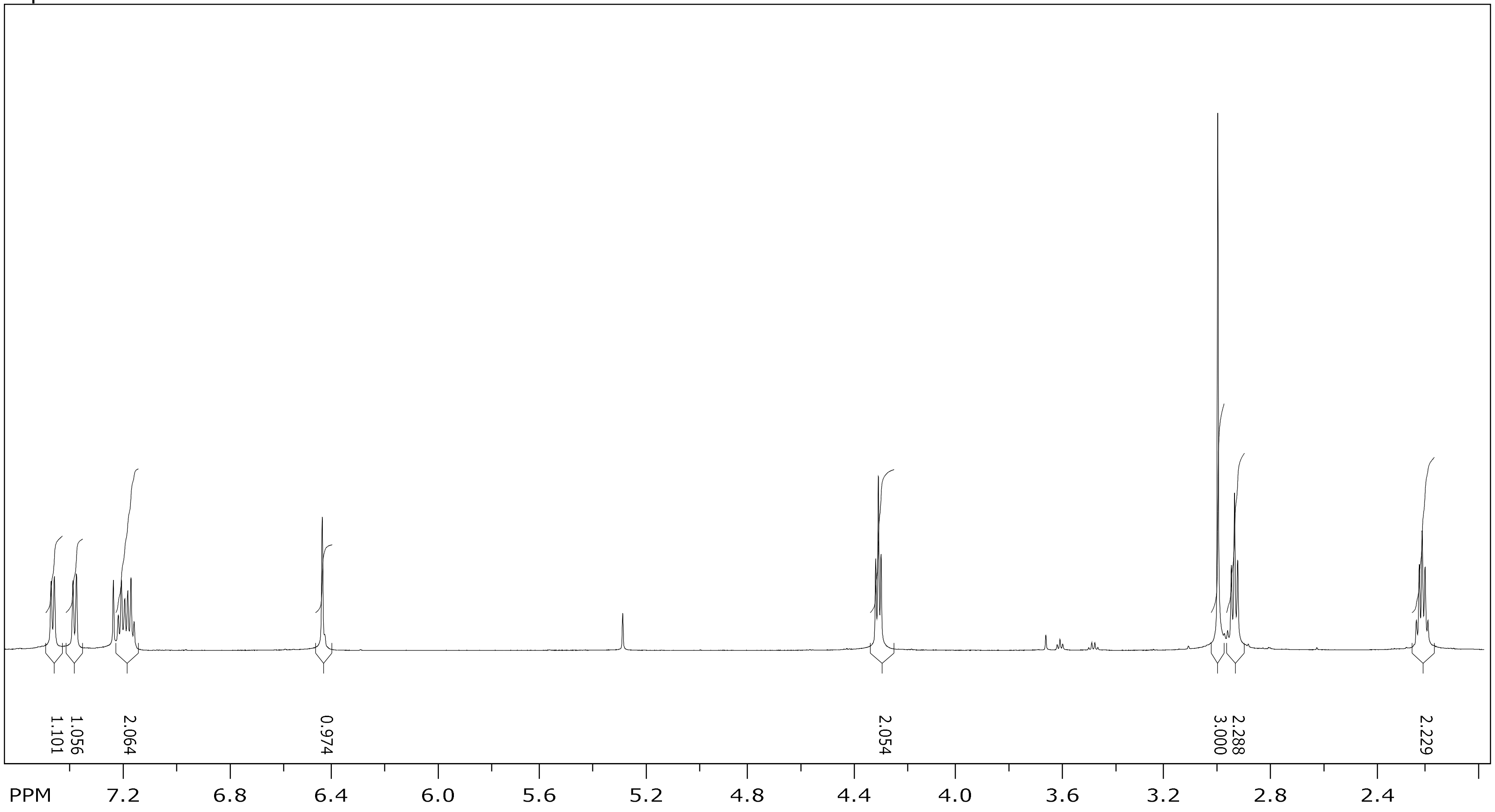


file: ...top\FID UMED\bo-33-10.02.15\10\fid expt: <zg30>
transmitter freq.: 600.263707 MHz
time domain size: 65536 points
width: 12335.53 Hz = 20.5502 ppm = 0.188225 Hz/pt
number of scans: 16

freq. of 0 ppm: 600.260021 MHz
processed size: 32768 complex points
LB: 0.000 GF: 0.0000
Hz/cm: 137.017 ppm/cm: 0.22826

SpinWorks 3: 4b

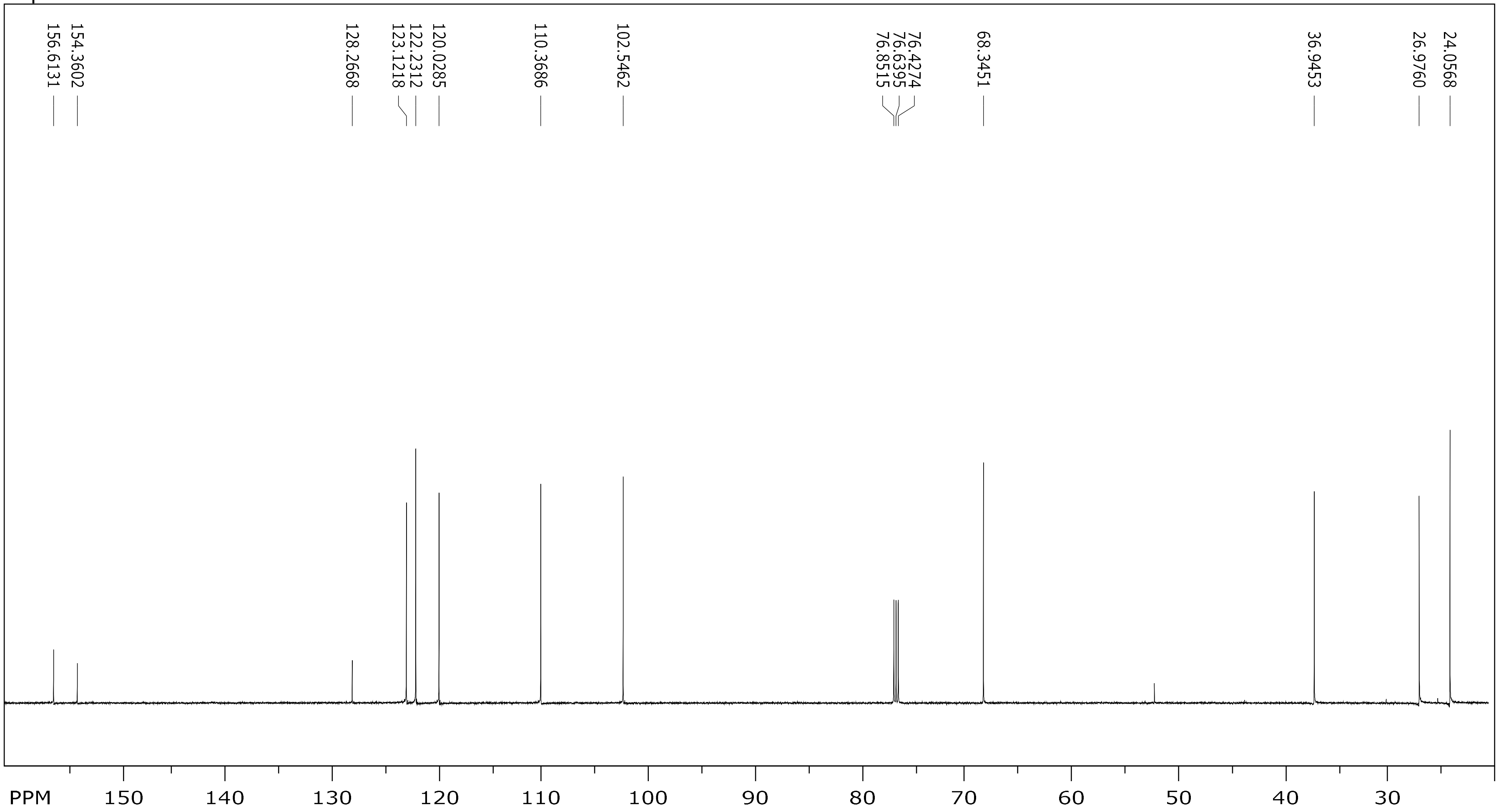


file: ...taa\Desktop\FID UMED\bo-33-C\1\fid expt: <zgpg>
transmitter freq.: 150.950591 MHz
time domain size: 65536 points
width: 36057.69 Hz = 238.8708 ppm = 0.550197 Hz/pt
number of scans: 512

freq. of 0 ppm: 150.935424 MHz
processed size: 32768 complex points
LB: 0.000 GF: 0.0000
Hz/cm: 850.799 ppm/cm: 5.63628

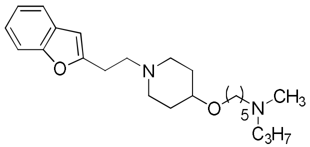


### 3. $^{1}$H and $^{13}$C NMR spectral data of final compounds 1b,c,e,f and 2b,c,e,f

SpinWorks 3: 1b

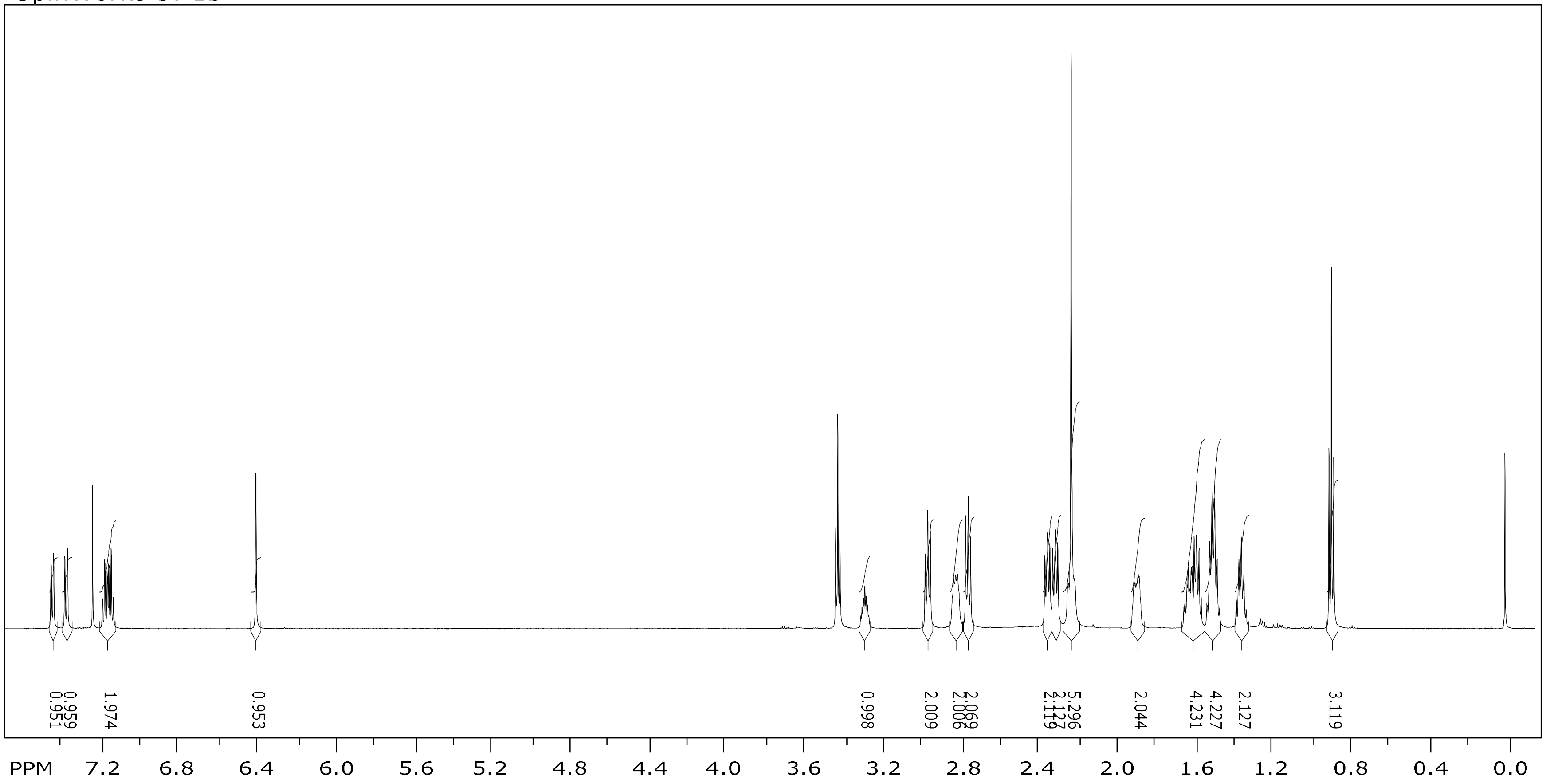


file: ...top\FID UMED\bo-54-19.05.15\10\fid expt: <zg30>
transmitter freq.: 600.263707 MHz
time domain size: 65536 points
width: 12335.53 Hz = 20.5502 ppm = 0.188225 Hz/pt
number of scans: 16

freq. of 0 ppm: 600.260018 MHz
processed size: 32768 complex points
LB: 0.000 GF: 0.0000
Hz/cm: 188.894 ppm/cm: 0.31468

SpinWorks 3: 1b

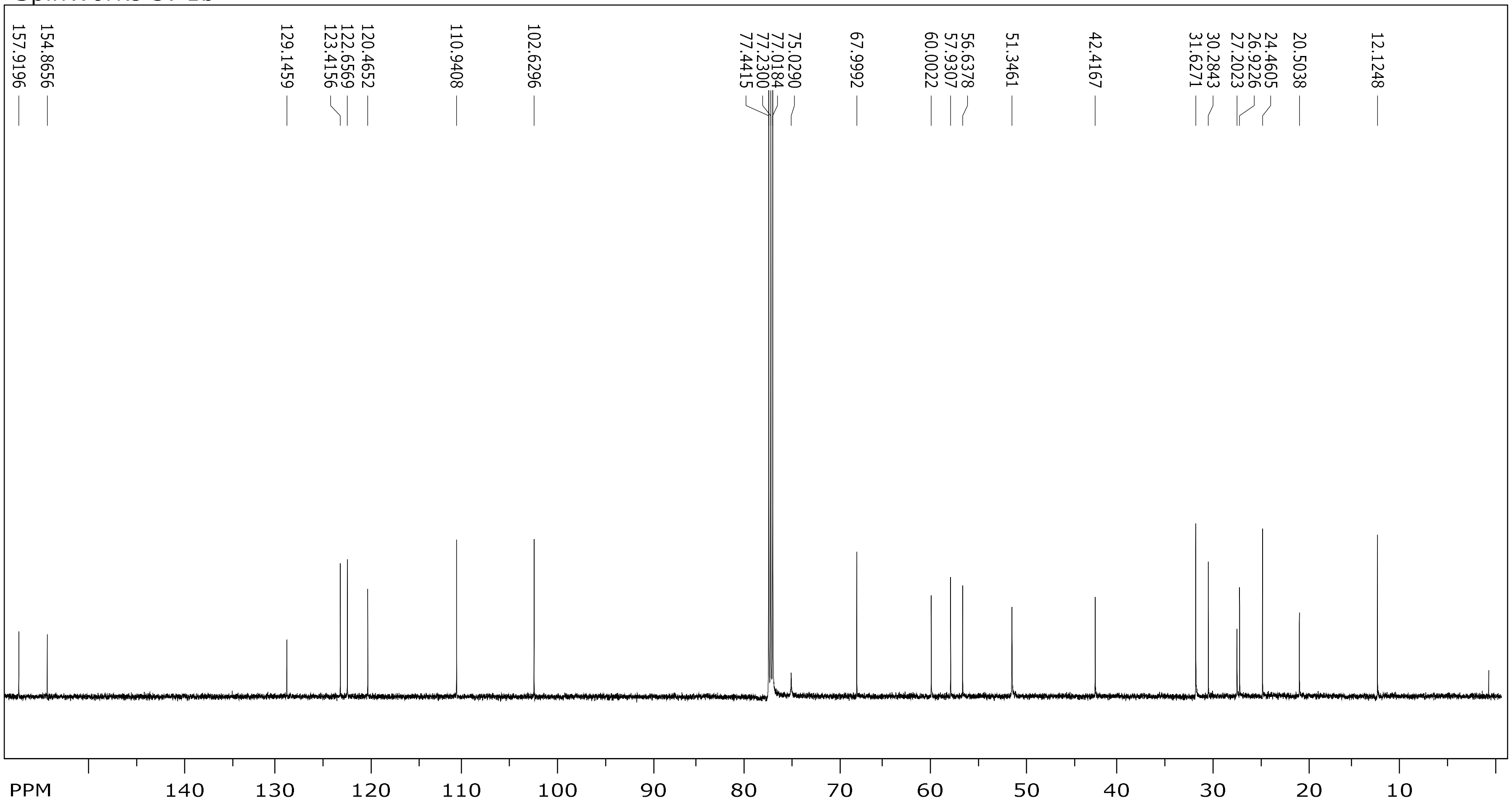


file: ...top\FID UMED\bo-54-19.05.15\11\fid expt: <zgpg30>
transmitter freq.: 150.950591 MHz
time domain size: 65536 points
width: 36057.69 Hz = 238.8708 ppm = 0.550197 Hz/pt
number of scans: 1024

freq. of 0 ppm: 150.935462 MHz
processed size: 32768 complex points
LB: 0.000 GF: 0.0000
Hz/cm: 970.027 ppm/cm: 6.42612

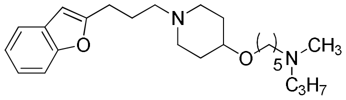


SpinWorks 3: 1c

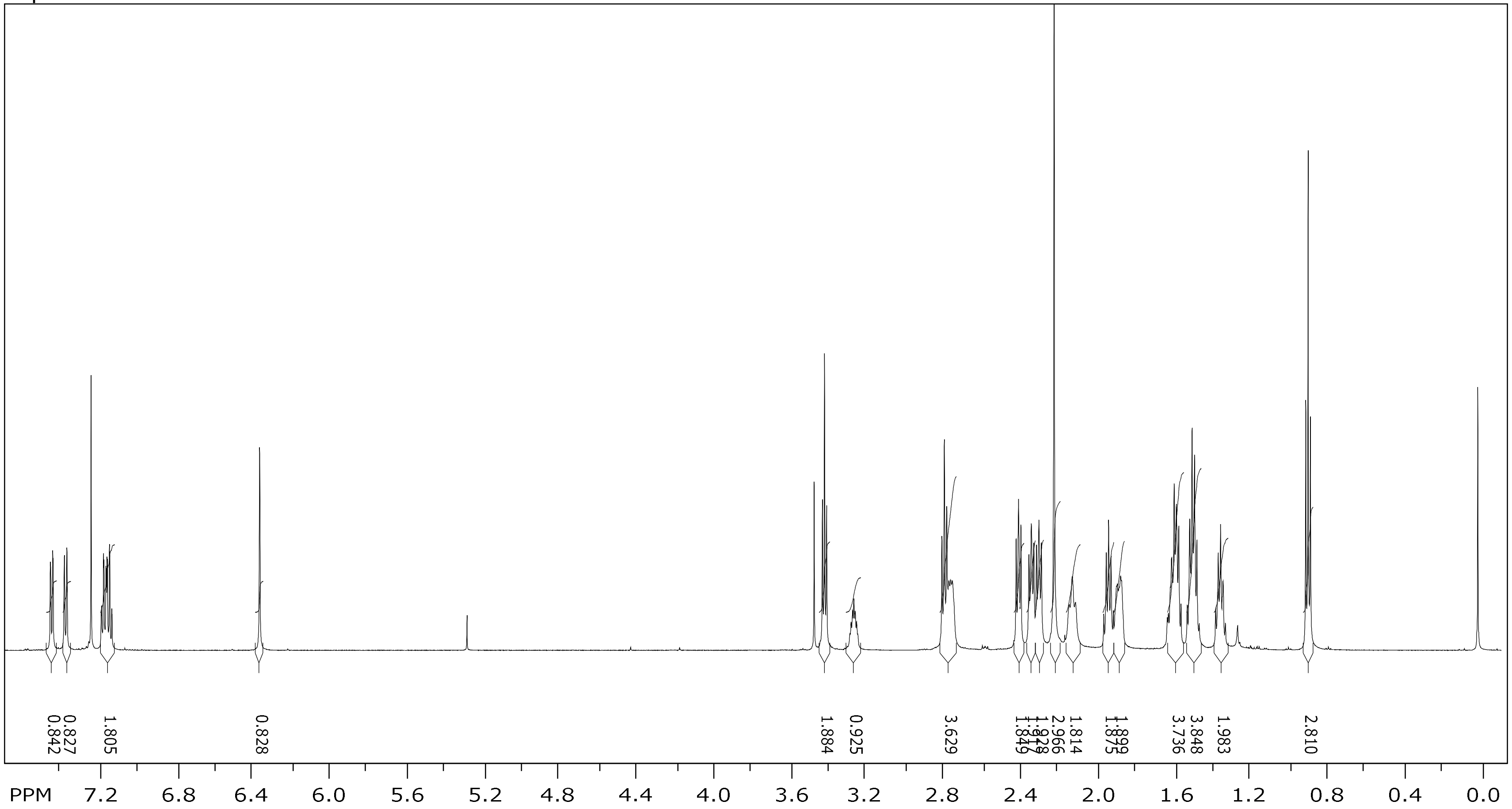


file: ...ataa\Desktop\FID UMED\bo-49\10\fid expt: <zg30>
transmitter freq.: 600.263707 MHz
time domain size: 65536 points
width: 12335.53 Hz = 20.5502 ppm = 0.188225 Hz/pt
number of scans: 16

freq. of 0 ppm: 600.260019 MHz
processed size: 32768 complex points
LB: 0.000 GF: 0.0000
Hz/cm: 188.102 ppm/cm: 0.31337

SpinWorks 3: 1c

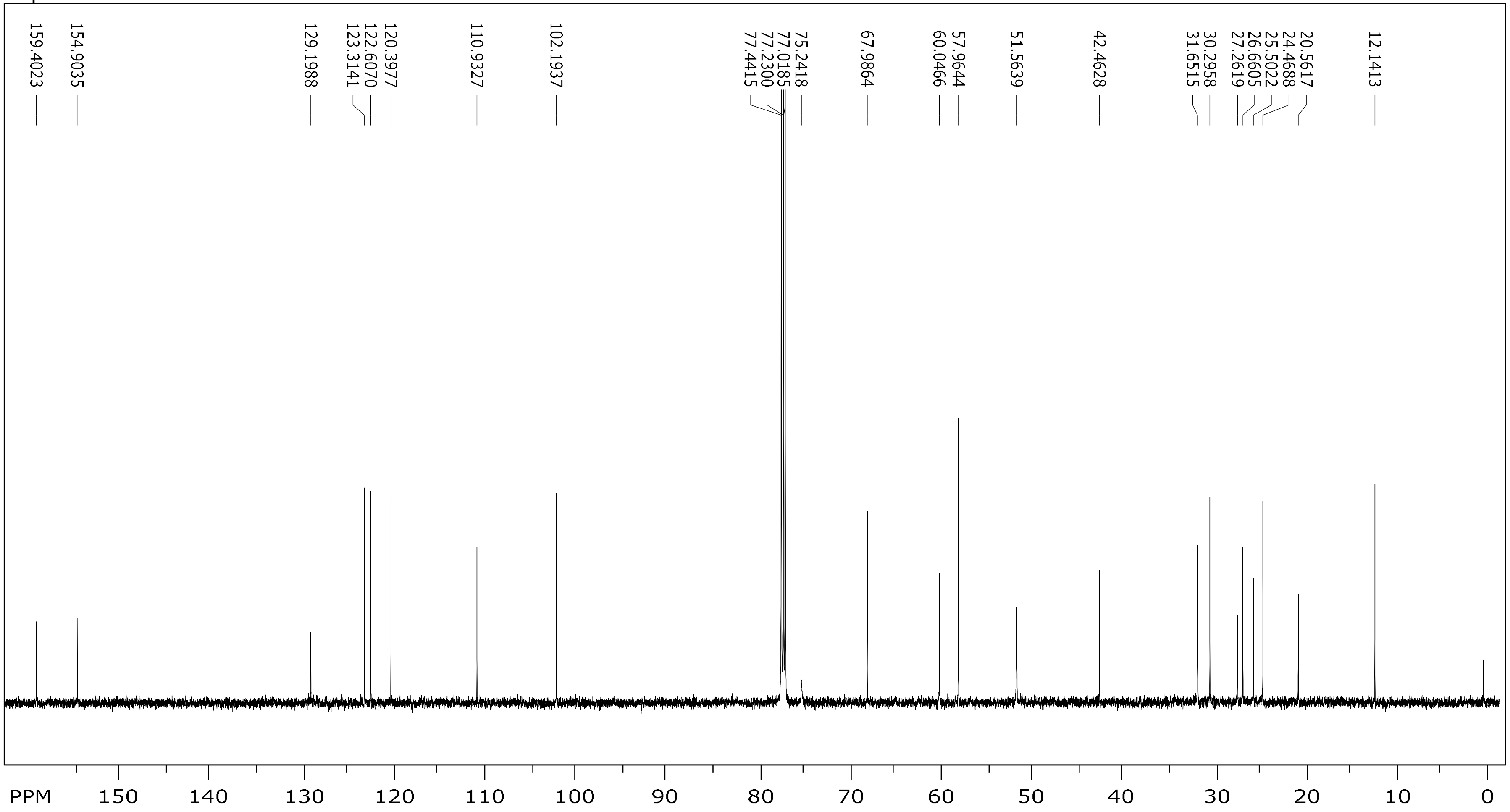


file: ...ataa\Desktop\FID UMED\bo-49\11\fid expt: <zgpg30>
transmitter freq.: 150.950591 MHz
time domain size: 65536 points
width: 36057.69 Hz = 238.8708 ppm = 0.550197 Hz/pt
number of scans: 1024

freq. of 0 ppm: 150.935461 MHz
processed size: 32768 complex points
LB: 0.000 GF: 0.0000
Hz/cm: 993.178 ppm/cm: 6.57949

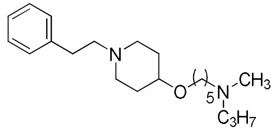


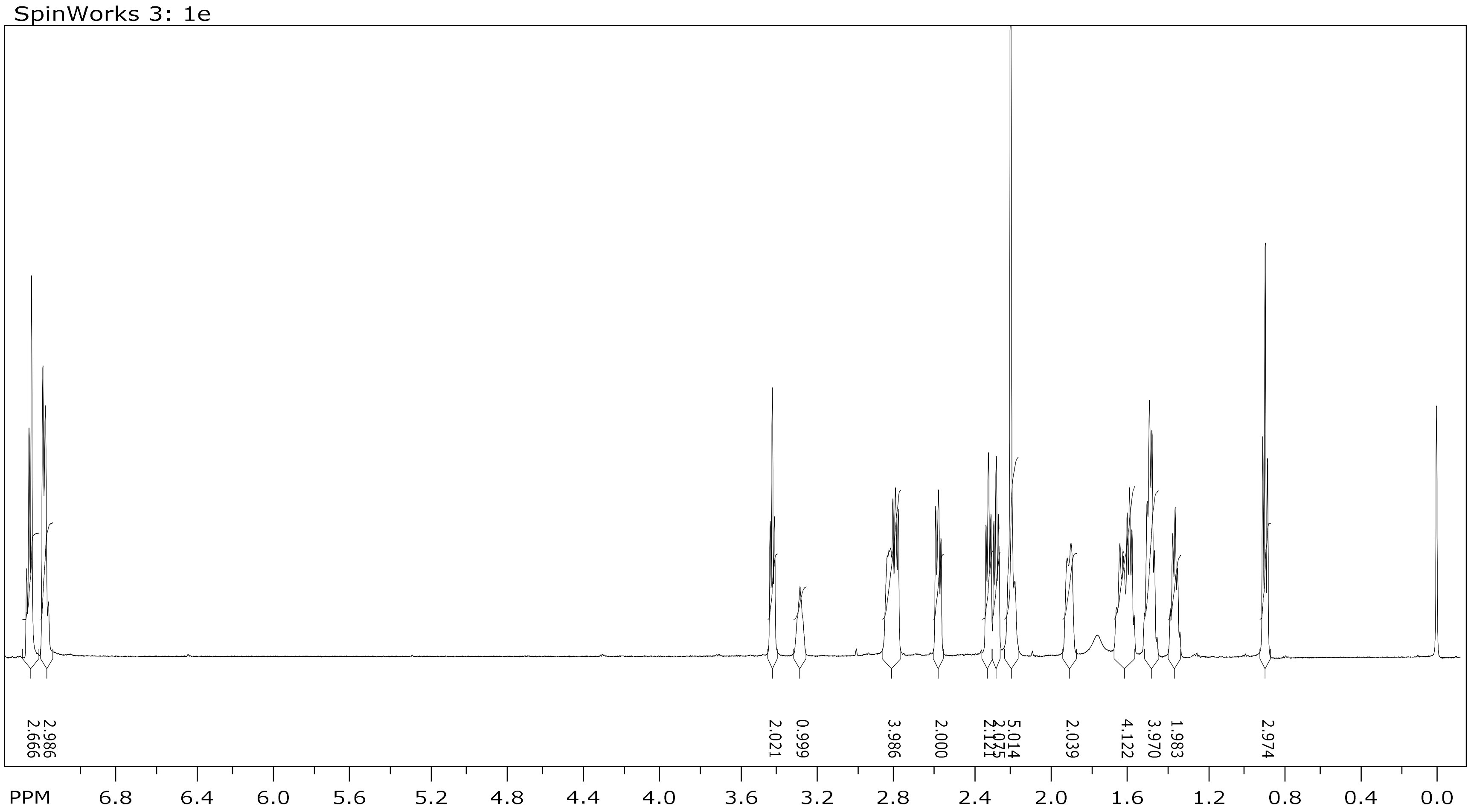


file: ...ataa\Desktop\FID UMED\bo-48\10\fid expt: <zg30>
transmitter freq.: 600.263707 MHz
time domain size: 65536 points
width: 12335.53 Hz = 20.5502 ppm = 0.188225 Hz/pt
number of scans: 16

freq. of 0 ppm: 600.260019 MHz
processed size: 32768 complex points
LB: 0.000 GF: 0.0000
Hz/cm: 180.578 ppm/cm: 0.30083

SpinWorks 3: 1e

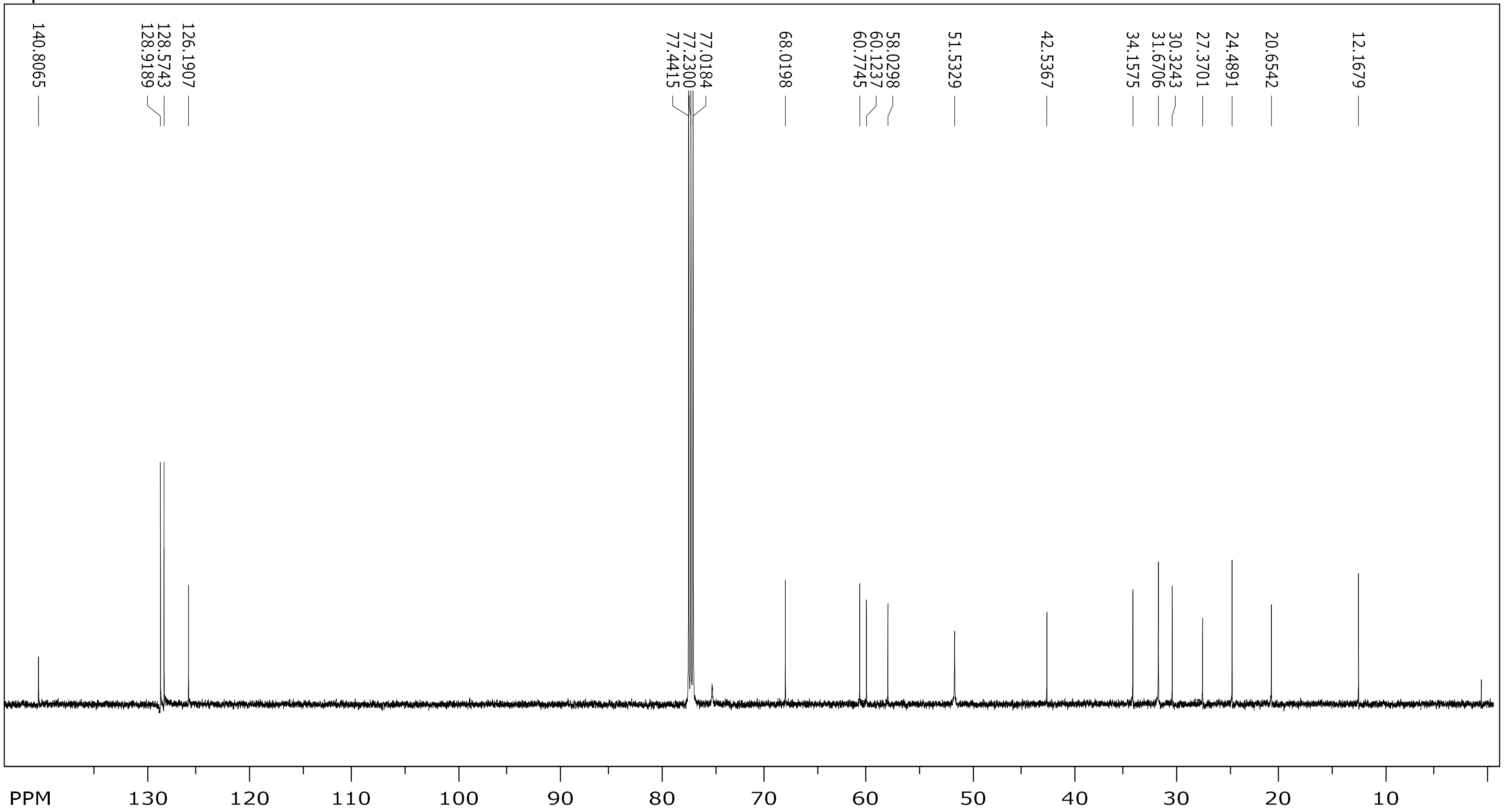


file: ...aa\Desktop\FID UMED\bo-48-C\11\fid expt: <zgpg30>
transmitter freq.: 150.950591 MHz
time domain size: 65536 points
width: 36057.69 Hz = 238.8708 ppm = 0.550197 Hz/pt
number of scans: 1024

freq. of 0 ppm: 150.935460 MHz
processed size: 32768 complex points
LB: 0.000 GF: 0.0000
Hz/cm: 876.266 ppm/cm: 5.80498

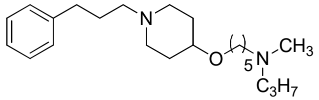


SpinWorks 3: 1f

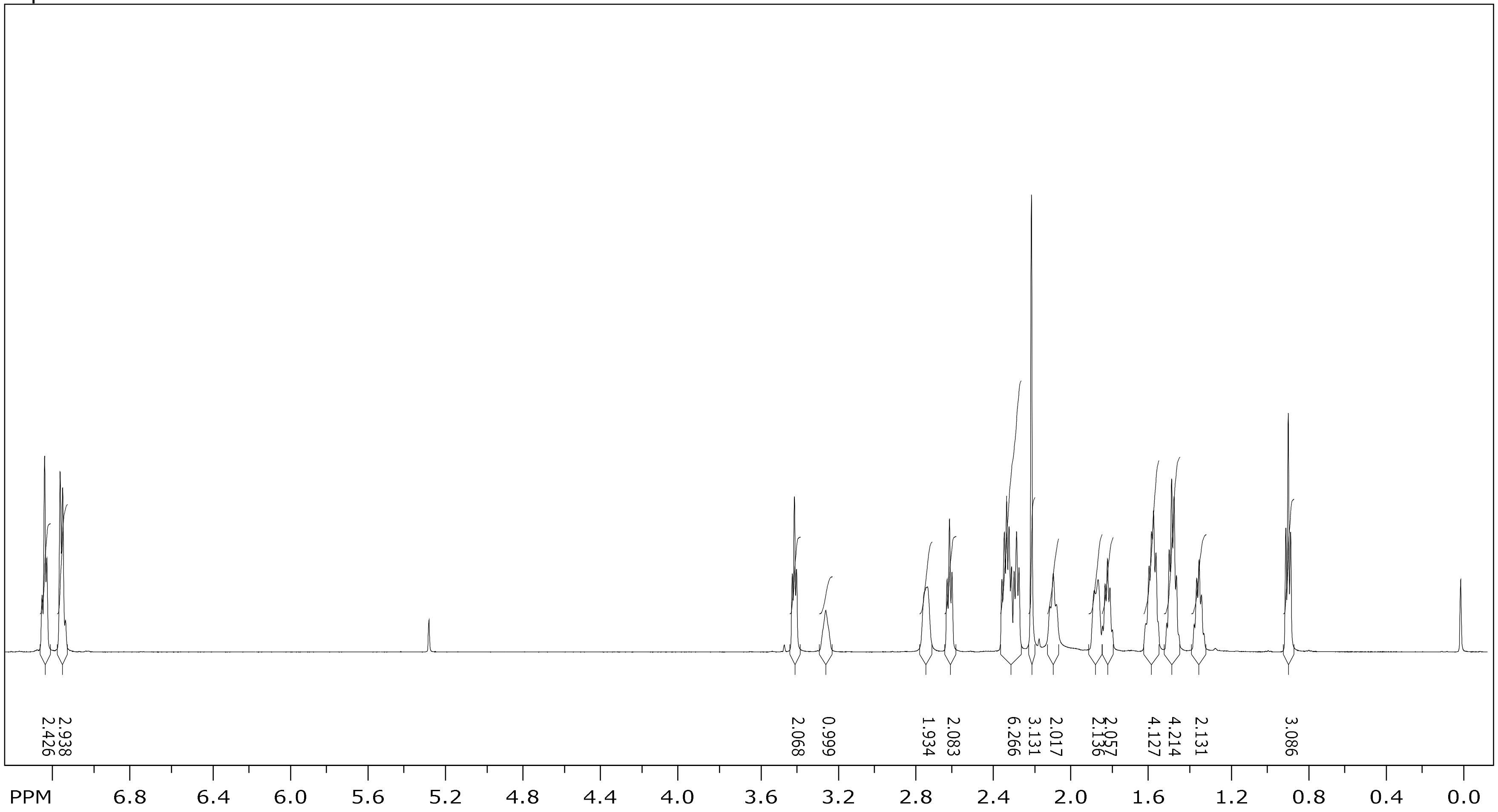


file: ...aa\Desktop\FID UMED\bo-51-C\10\fid expt: <zg30>
transmitter freq.: 600.263707 MHz
time domain size: 65536 points
width: 12335.53 Hz = 20.5502 ppm = 0.188225 Hz/pt
number of scans: 16

freq. of 0 ppm: 600.260018 MHz
processed size: 32768 complex points
LB: 0.000 GF: 0.0000
Hz/cm: 182.558 ppm/cm: 0.30413

SpinWorks 3: 1f

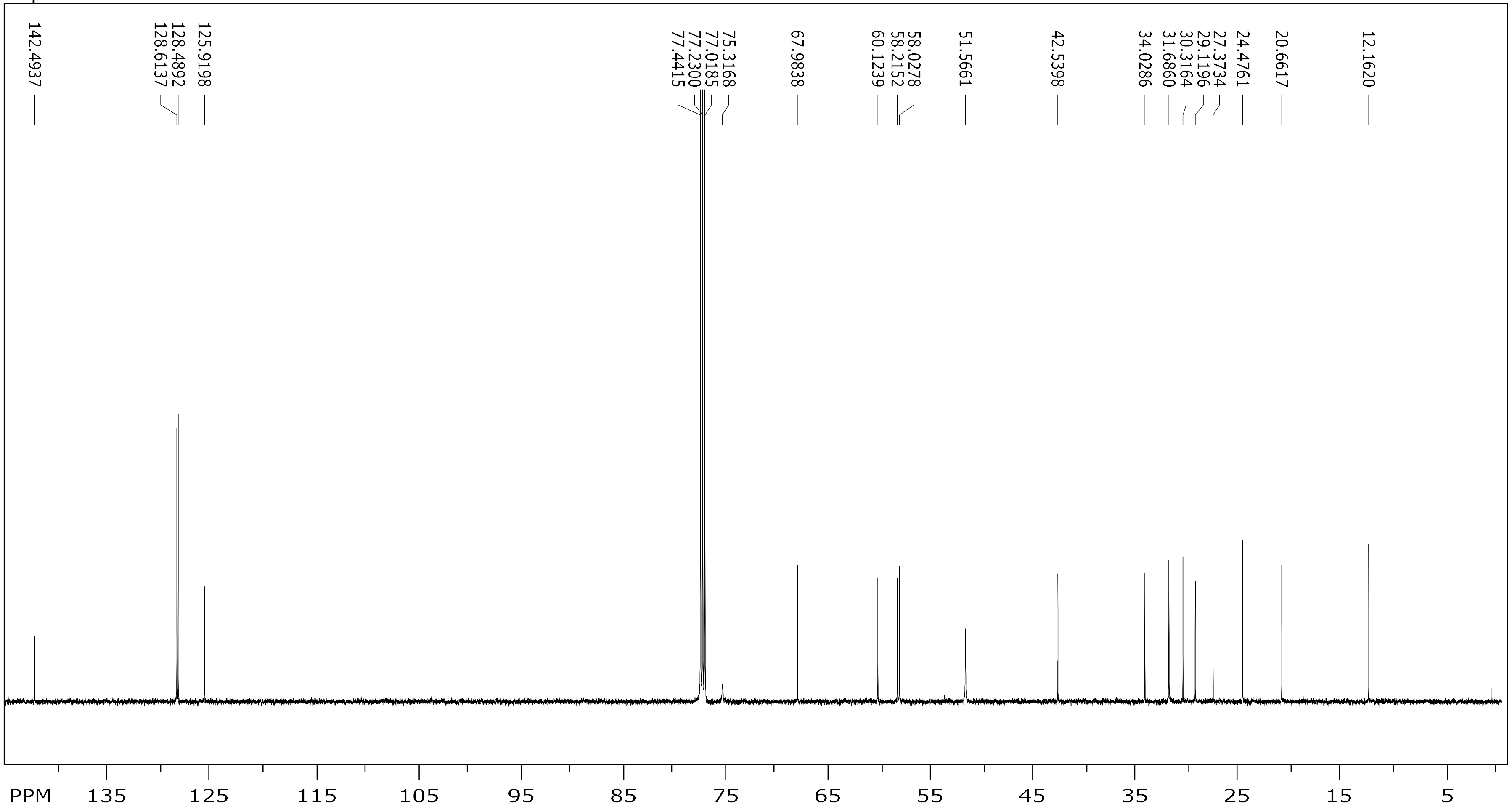


file: ...aa\Desktop\FID UMED\bo-51-C\11\fid expt: <zgpg30>
transmitter freq.: 150.950591 MHz
time domain size: 65536 points
width: 36057.69 Hz = 238.8708 ppm = 0.550197 Hz/pt
number of scans: 1024

freq. of 0 ppm: 150.935461 MHz
processed size: 32768 complex points
LB: 0.000 GF: 0.0000
Hz/cm: 883.211 ppm/cm: 5.85099

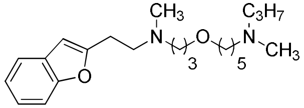

SpinWorks 3: 2b

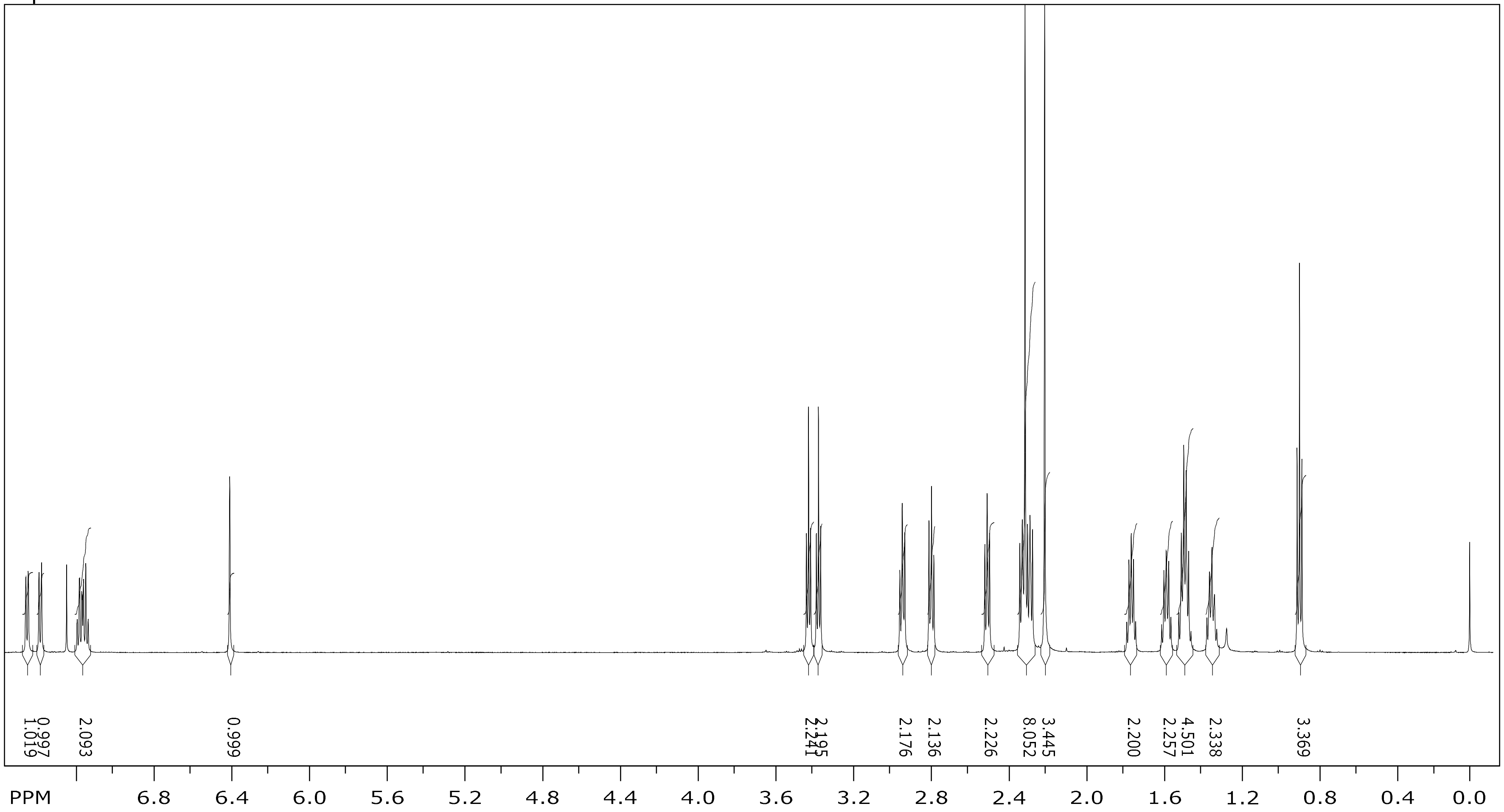


file: ...top\FID UMED\bo-55-19.05.15\11\fid expt: <zg30>
transmitter freq.: 600.263707 MHz
time domain size: 65536 points
width: 12335.53 Hz = 20.5502 ppm = 0.188225 Hz/pt
number of scans: 16

freq. of 0 ppm: 600.260019 MHz
processed size: 32768 complex points
LB: 0.000 GF: 0.0000
Hz/cm: 184.934 ppm/cm: 0.30809

SpinWorks 3: 2b

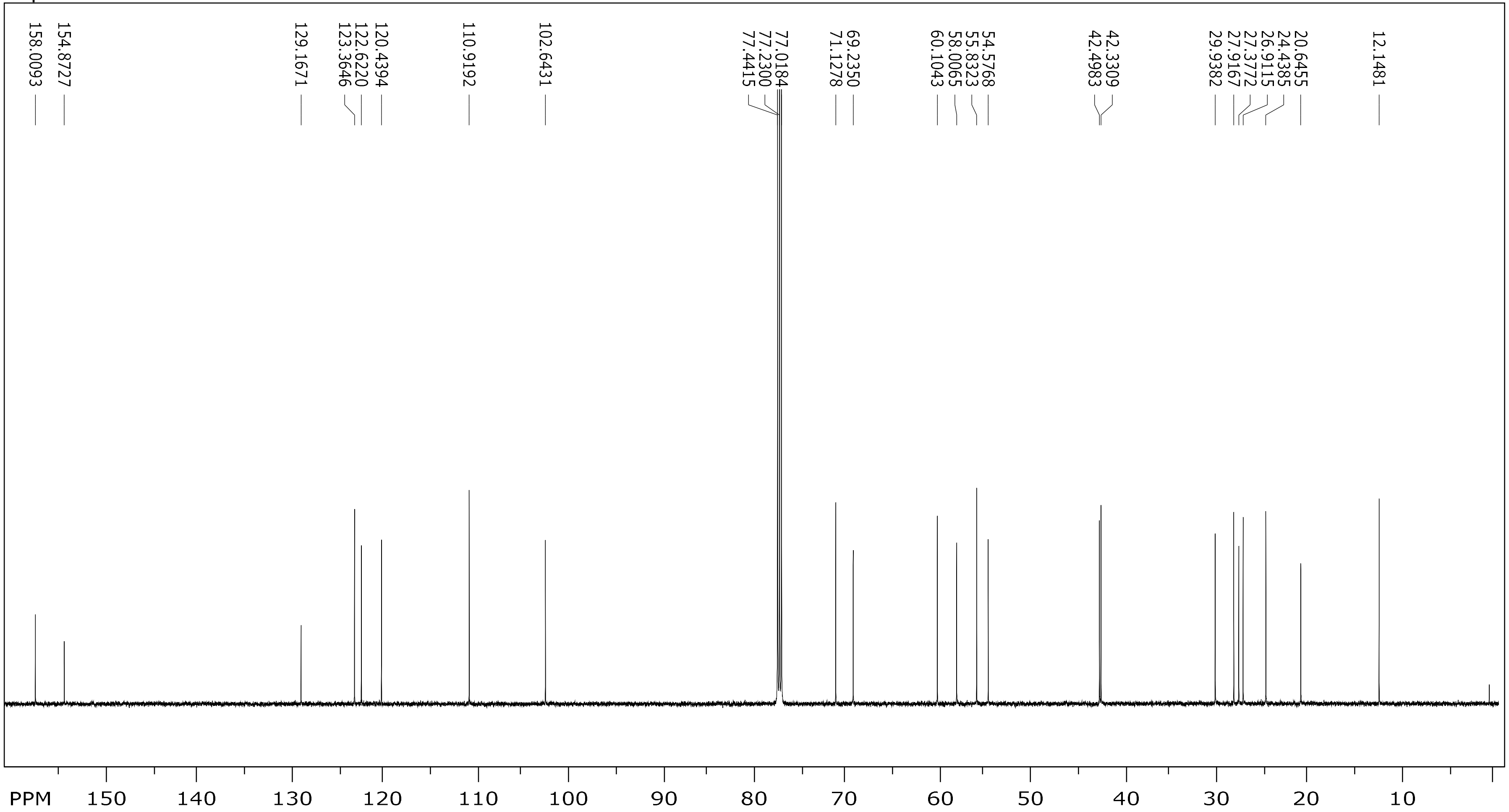


file: ...top\FID UMED\bo-55-19.05.15\10\fid expt: <zgpg30>
transmitter freq.: 150.950591 MHz
time domain size: 65536 points
width: 36057.69 Hz = 238.8708 ppm = 0.550197 Hz/pt
number of scans: 1024

freq. of 0 ppm: 150.935463 MHz
processed size: 32768 complex points
LB: 0.000 GF: 0.0000
Hz/cm: 979.288 ppm/cm: 6.48747

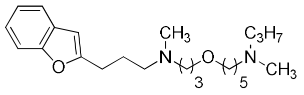


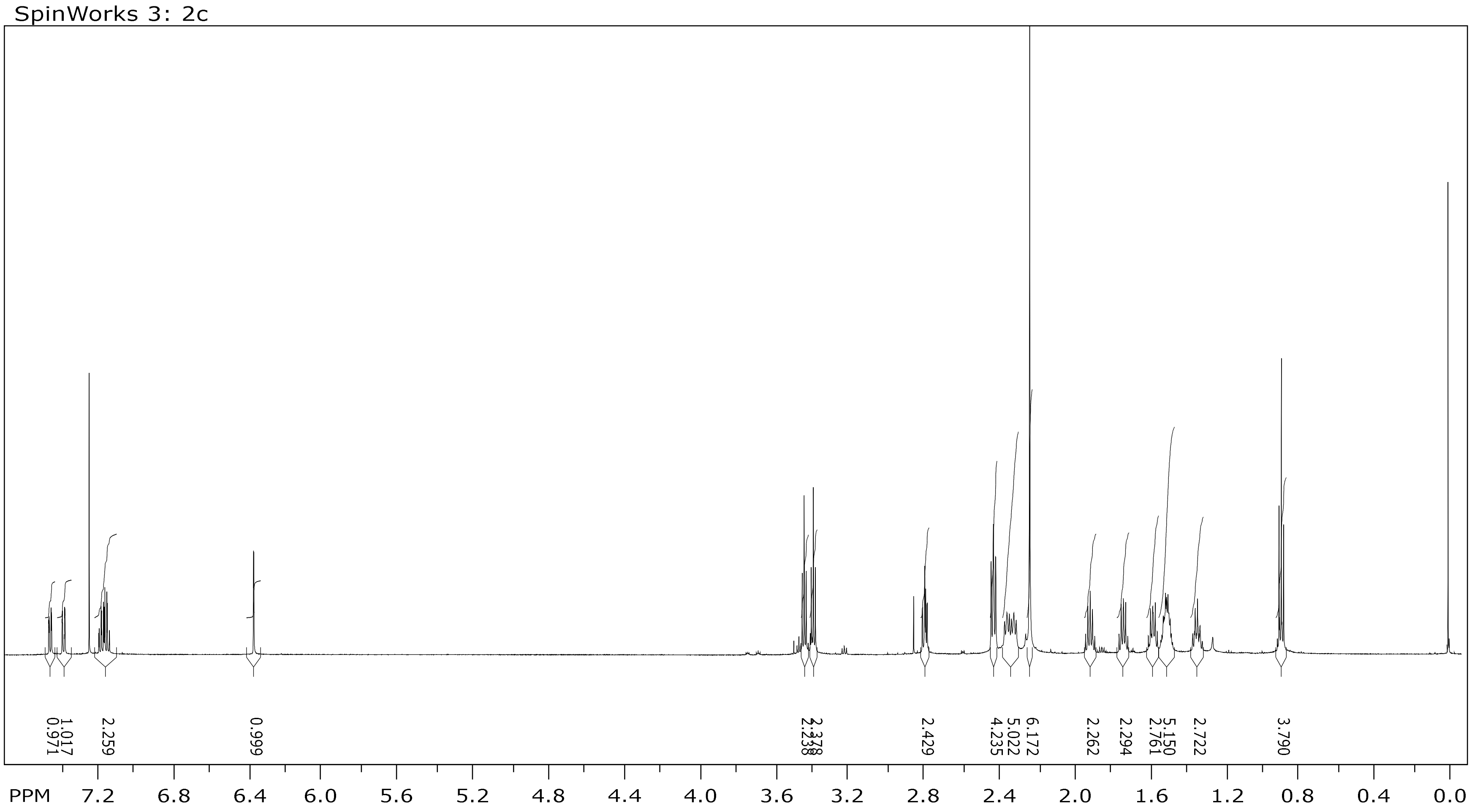


file: ...op\FID UMED\bo-46-06.10.2015\1\fid expt: <zg30>
transmitter freq.: 600.263707 MHz
time domain size: 65536 points
width: 12335.53 Hz = 20.5502 ppm = 0.188225 Hz/pt
number of scans: 16

freq. of 0 ppm: 600.260018 MHz
processed size: 32768 complex points
LB: 0.000 GF: 0.0000
Hz/cm: 186.914 ppm/cm: 0.31139

SpinWorks 3: 2c

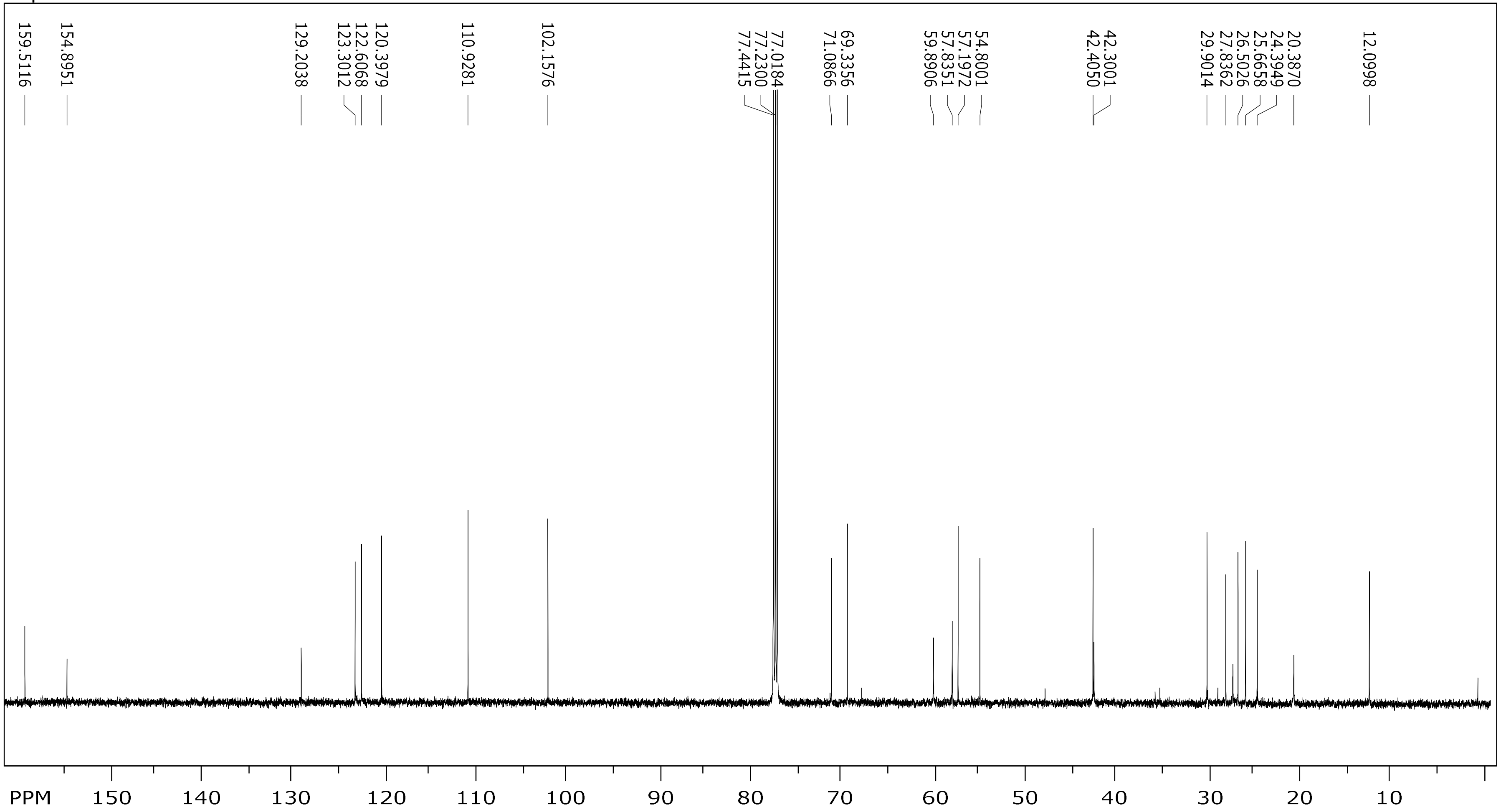


file: ...aa\Desktop\FID UMED\bo-46-C\10\fid expt: <zgpg30>
transmitter freq.: 150.950591 MHz
time domain size: 65536 points
width: 36057.69 Hz = 238.8708 ppm = 0.550197 Hz/pt
number of scans: 3072

freq. of 0 ppm: 150.935462 MHz
processed size: 32768 complex points
LB: 0.000 GF: 0.0000
Hz/cm: 983.918 ppm/cm: 6.51814

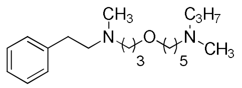


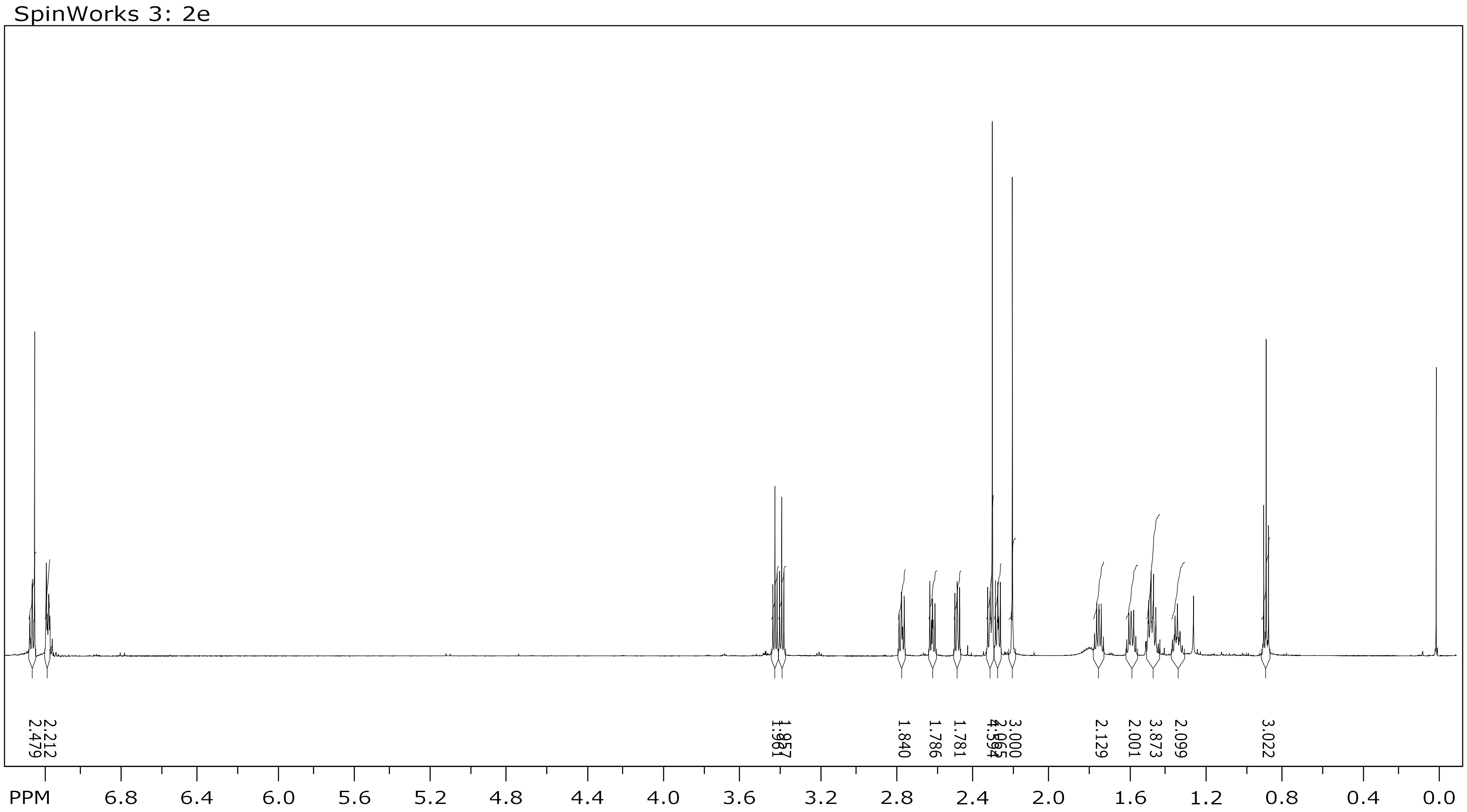


file: ...ktop\FID UMED\bo-45-24.03.15\1\fid   expt: <zg30>
transmitter freq.: 600.263707 MHz
time domain size: 65536 points
width: 12335.53 Hz = 20.5502 ppm = 0.188225 Hz/pt
number of scans: 16

freq. of 0 ppm: 600.260019 MHz
processed size: 32768 complex points
LB: 0.000    GF: 0.0000
Hz/cm: 180.578    ppm/cm: 0.30083

SpinWorks 3: 2e

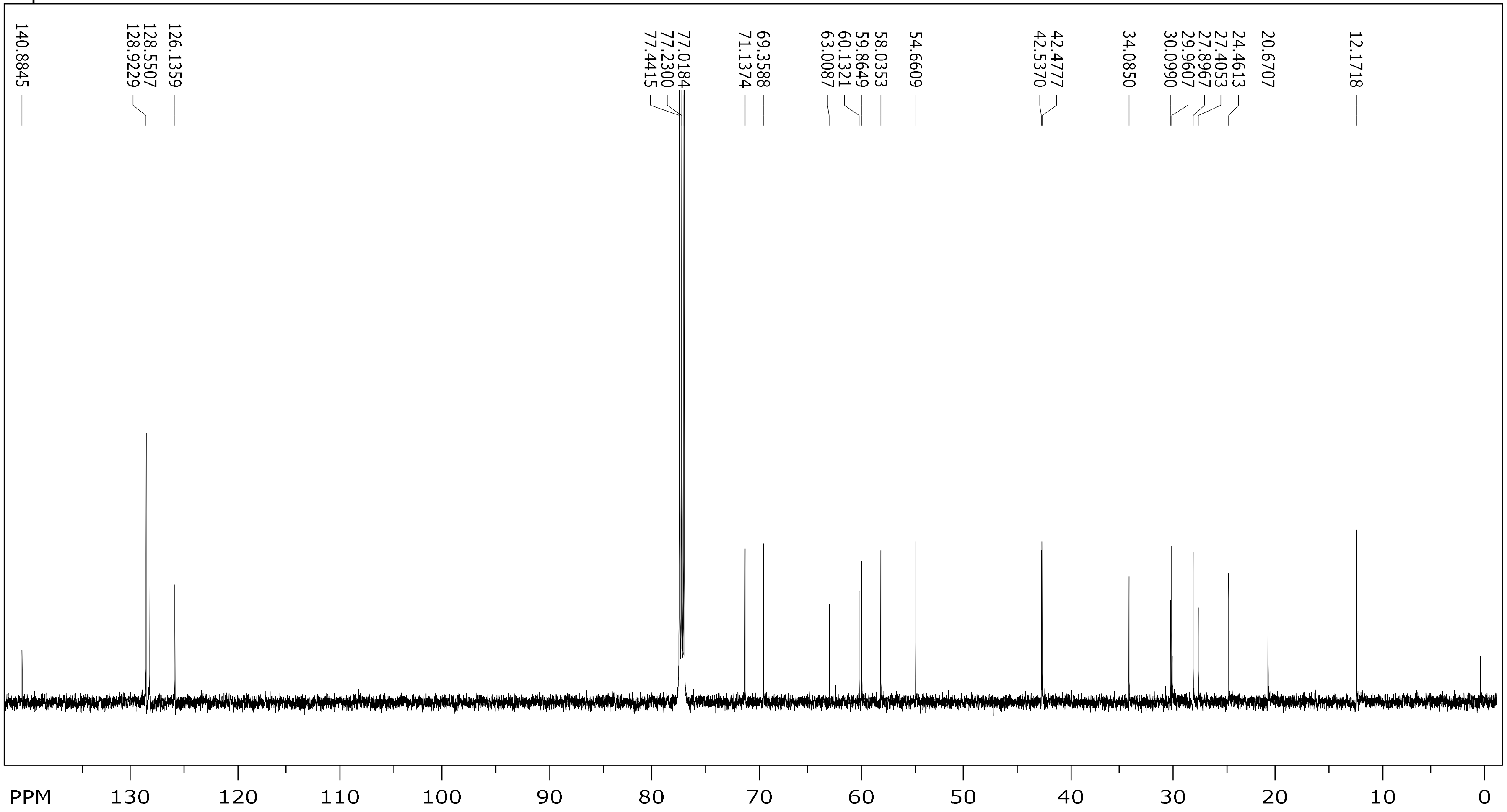


file: ...p\FID UMED\bo-45-05.02.2015\12\fid expt: <zgpg30>
transmitter freq.: 150.950591 MHz
time domain size: 65536 points
width: 36057.69 Hz = 238.8708 ppm = 0.550197 Hz/pt
number of scans: 1024

freq. of 0 ppm: 150.935461 MHz
processed size: 32768 complex points
LB: 0.000 GF: 0.0000
Hz/cm: 869.320 ppm/cm: 5.75897

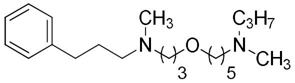


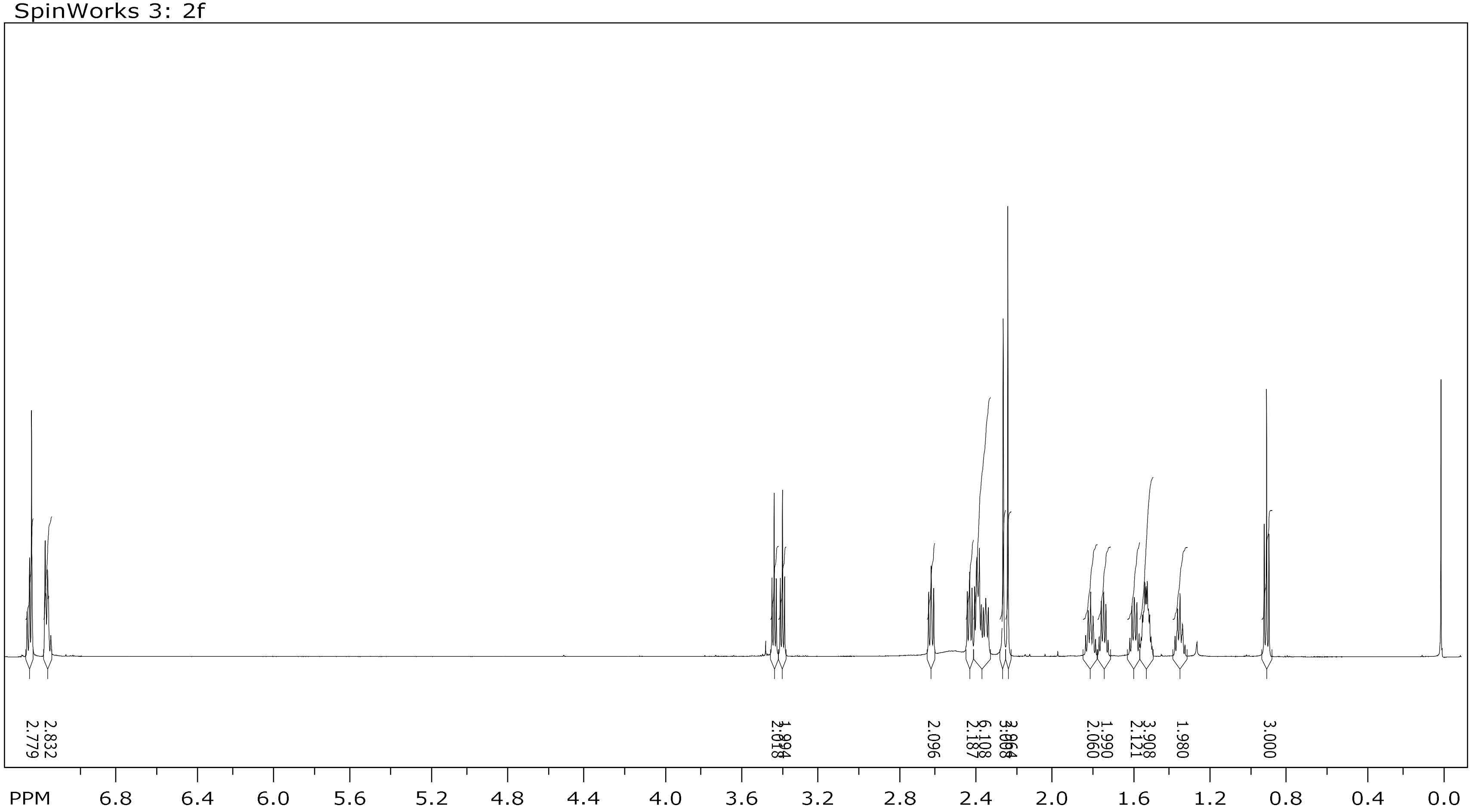


file: ...p\FID UMED\bo-53-21.07.2016\10\fid expt: <zg30>
transmitter freq.: 600.263707 MHz
time domain size: 65536 points
width: 12335.53 Hz = 20.5502 ppm = 0.188225 Hz/pt
number of scans: 16

freq. of 0 ppm: 600.260018 MHz
processed size: 32768 complex points
LB: 0.000 GF: 0.0000
Hz/cm: 180.182 ppm/cm: 0.30017

SpinWorks 3: 2f

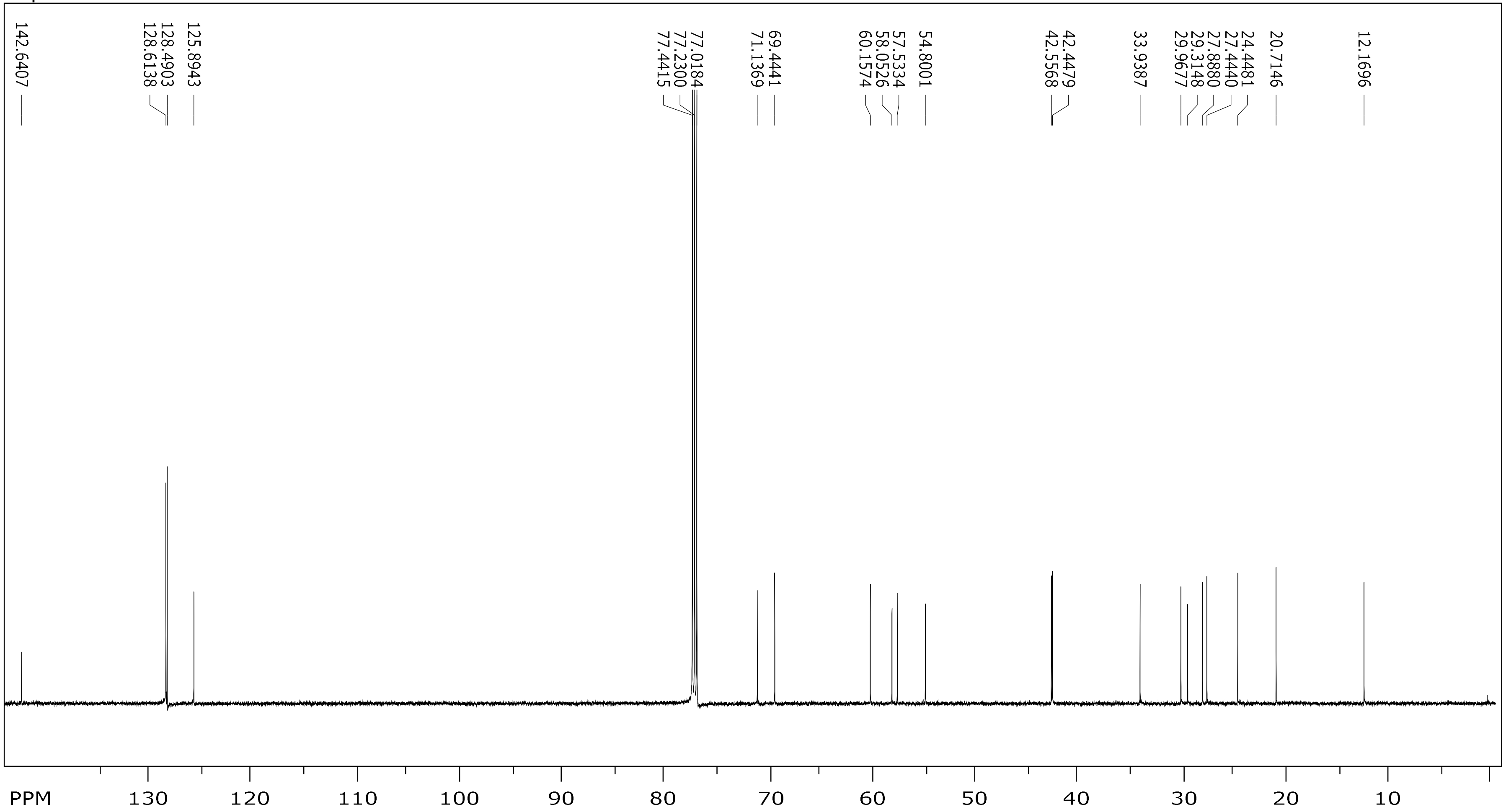


file: ...FID UMED\bo-53-2-08.05.2015\11\fid expt: <zgpg30>
transmitter freq.: 150.950591 MHz
time domain size: 65536 points
width: 36057.69 Hz = 238.8708 ppm = 0.550197 Hz/pt
number of scans: 1024

freq. of 0 ppm: 150.935461 MHz
processed size: 32768 complex points
LB: 0.000 GF: 0.0000
Hz/cm: 875.108 ppm/cm: 5.79731

# Supplementary Material B

*2.2. Pharmacology*

2.2.1. Antagonism by ADS-003 (1a) and thioperamide of the inhibitory effect of R-(-)- α-methylhistamine (R-α-MH) on the electrically induced contraction of guinea-pig ileum strips

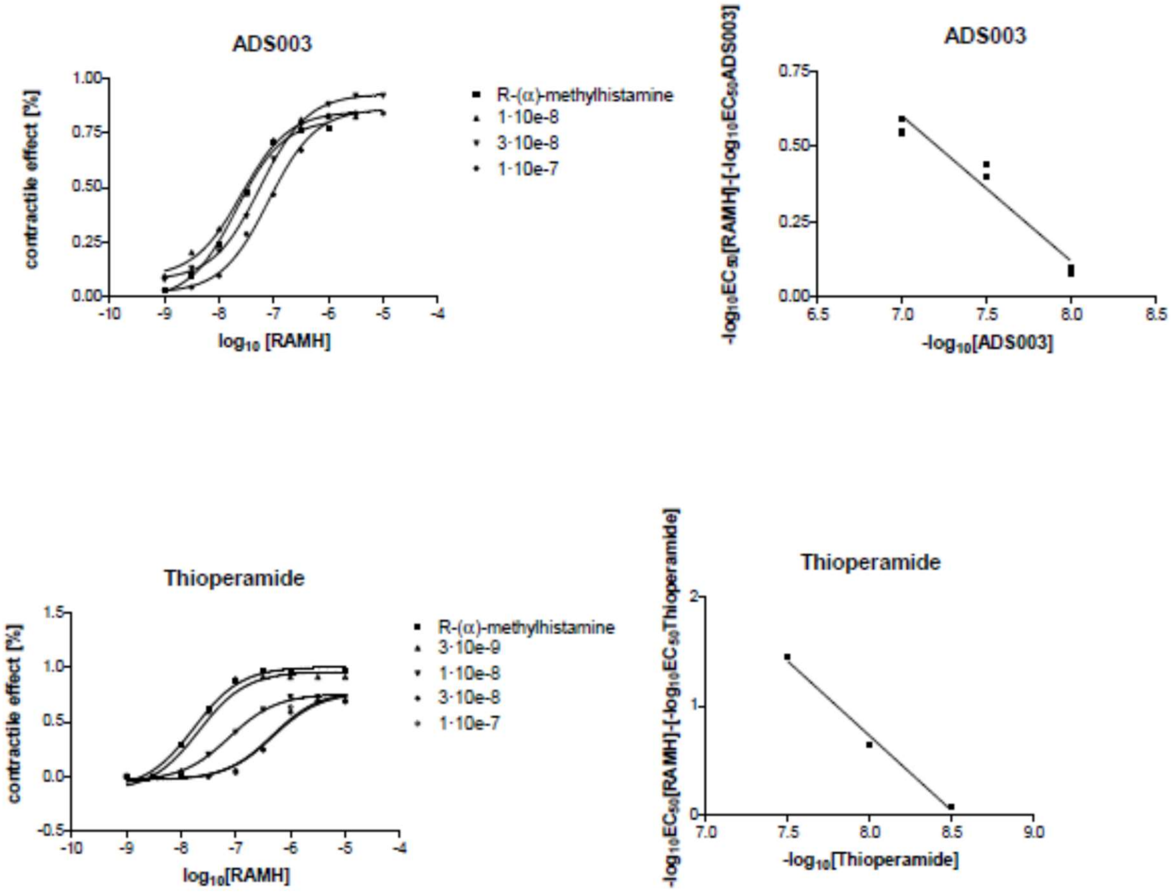


Figure 2 Antagonism by ADS-003 (1a) and thioperamide of the inhibitory effect of R-(-)- α-methylhistamine (R-α-MH) on the electrically induced contraction of guinea-pig ileum strips

*2.2.2 Histamine $H_3$ receptor affinity*

*Saturation of rat and human $H_3$ receptors*

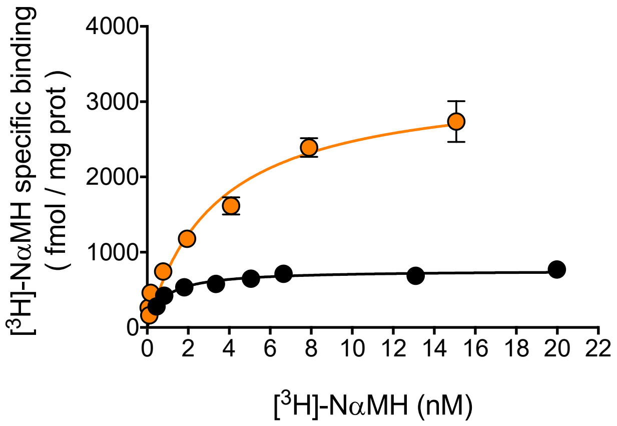


Figure 3. Saturation of rat and human $H_3R$.

*Competition binding of $H_3$ receptor ligands*

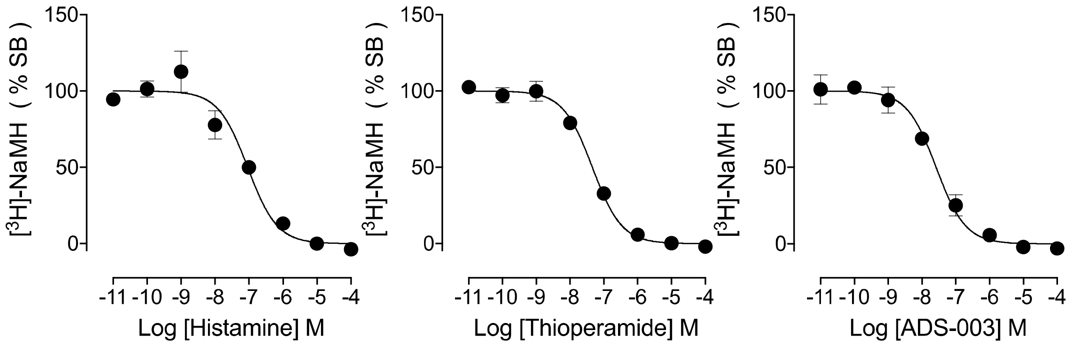


Figure 4 Competition binding of $H_3R$ ligands on rat $H_3$ receptor.

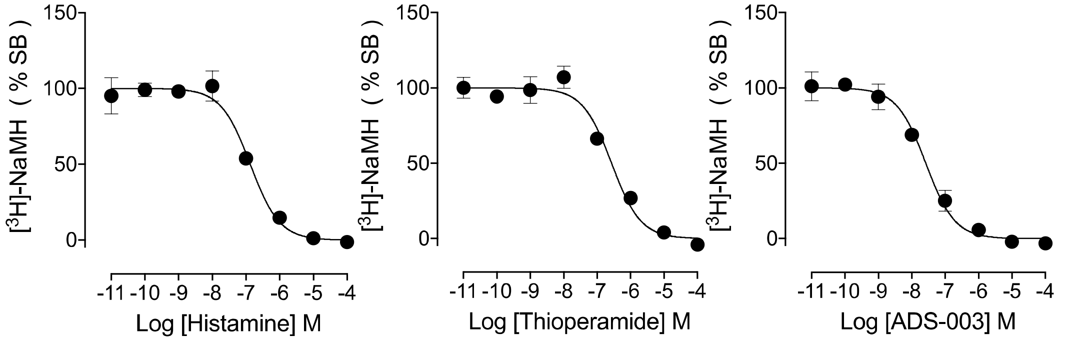


Figure 5: Competition binding of $H_3R$ ligands on human $H_3$ receptor.
